# Supplementary material for: [20(22)E]-Lanostane Triterpenes from the Fungus Ganoderma australe
Source: J Fungi (Basel). 2022 May 12;8(5):503. doi: 10.3390/jof8050503 (PMC9145439; doi:10.3390/jof8050503)
Supplement: Supplementary file 1 [file jof-08-00503-s001.zip › jof-1683631-supplementary.pdf]

Supplementary Materials for

[20(22)*E*]-Lanostane Triterpenes from the Fungus *Ganoderma australe*

Lin Zhou [1,†], Li-Li Guo [1,†], Masahiko Isaka [2], Zheng-Hui Li [1,*] and He-Ping Chen [1,*]

[1] School of Pharmaceutical Sciences, South-Central Minzu University, Wuhan 430074, China; 2019110443@mail.scuec.edu.cn (L.Z.); g15513998615@163.com (L.-L.G.)
[2] National Center for Genetic Engineering and Biotechnology (BIOTEC), 113 Thailand Science Park, Phaholyothin Road, Klong Luang, Pathumthani 12120, Thailand; isaka@biotec.or.th
* Correspondence: lizhenghui@mail.scuec.edu.cn (Z.-H.L.); chenhp@mail.scuec.edu.cn (H.-P.C.)
† These authors contributed equally to this work.

Contents

1. Supplementary Figures 1
Figure S1. ^{1}H NMR spectrum of **1** (600 MHz, CD_3OD). 1
Figure S2. ^{13}C and DEPT NMR spectra of **1** (150 MHz, CD_3OD). 1
Figure S3. HSQC spectrum of **1**. 2
Figure S4. HMBC spectrum of **1**. 2
Figure S5. ^{1}H-^{1}H COSY spectrum of **1**. 3
Figure S6. ROESY spectrum of **1**. 3
Figure S7. HRESIMS of **1**. 4
Figure S8. ^{1}H NMR spectrum of **2** (600 MHz, $CDCl_3$). 5
Figure S9. ^{13}C and DEPT NMR spectra of **2** (150 MHz, $CDCl_3$). 5
Figure S10. HSQC spectrum of **2**. 6
Figure S11. HMBC spectrum of **2**. 6
Figure S12. ^{1}H-^{1}H COSY spectrum of **2**. 7
Figure S13. ROESY spectrum of **2**. 7
Figure S14. HRESIMS of **2**. 8
Figure S15. ^{1}H NMR spectrum of **3** (500 MHz, $CDCl_3$). 9
Figure S16. ^{13}C and DEPT NMR spectra of **3** (125 MHz, $CDCl_3$). 9
Figure S17. HSQC spectrum of **3**. 10
Figure S18. HMBC spectrum of **3**. 10
Figure S19. ^{1}H-^{1}H COSY spectrum of **3**. 11
Figure S20. ROESY spectrum of **3**. 11
Figure S21. HRESIMS of **3**. 12
Figure S22. ^{1}H NMR spectrum of **4** (600 MHz, CD_3COCD_3). 13
Figure S23. ^{13}C and DEPT NMR spectra of **4** (150 MHz, CD_3COCD_3). 13
Figure S24. HSQC spectrum of **4**. 14
Figure S25. HMBC spectrum of **4**. 14
Figure S26. ^{1}H-^{1}H COSY spectrum of **4**. 15
Figure S27. ROESY spectrum of **4**. 15
Figure S28. HRESIMS of **4**. 16
Figure S29. ^{1}H NMR spectrum of **5** (600 MHz, $CDCl_3$). 17
Figure S30. ^{13}C and DEPT NMR spectra of **5** (150 MHz, $CDCl_3$). 17
Figure S31. HSQC spectrum of **5**. 18
Figure S32. HMBC spectrum of **5**. 18
Figure S33. ^{1}H-^{1}H COSY spectrum of **5**. 19
Figure S34. ROESY spectrum of **5**. 19
Figure S35. HRESIMS of **5**. 20
Figure S36. ^{1}H NMR spectrum of **6** (600 MHz, $CDCl_3$). 21
Figure S37. ^{13}C and DEPT NMR spectra of **6** (150 MHz, $CDCl_3$). 21
Figure S38. HSQC spectrum of **6**. 22
Figure S39. HMBC spectrum of **6**. 22
Figure S40. ^{1}H-^{1}H COSY spectrum of **6**. 23
Figure S41. ROESY spectrum of **6**. 23
Figure S42. HRESIMS of **6**. 24
Figure S43. ^{1}H NMR spectrum of **7** (800 MHz, $CDCl_3$). 25
Figure S44. ^{13}C NMR spectra of **7** (200 MHz, $CDCl_3$). 25
Figure S45. HSQC spectrum of **7**. 26
Figure S46. HMBC spectrum of **7**. 26

Figure S47. ^{1}H-^{1}H COSY spectrum of **7**. 27
Figure S48. ROESY spectrum of **7**. 27
Figure S49. HRESIMS of **7**. 28
Figure S50. ^{1}H NMR spectrum of **8** (600 MHz, $CDCl_3$). 29
Figure S51. ^{13}C NMR spectra of **8** (150 MHz, $CDCl_3$). 29
Figure S52. HSQC spectrum of **8**. 30
Figure S53. HMBC spectrum of **8**. 30
Figure S54. ^{1}H-^{1}H COSY spectrum of **8**. 31
Figure S55. ROESY spectrum of **8**. 31
Figure S56. HRESIMS of **8**. 32
Figure S57. ^{1}H NMR spectrum of **9** (600 MHz, $CDCl_3$). 33
Figure S58. ^{13}C and DEPT NMR spectra of **9** (150 MHz, $CDCl_3$). 33
Figure S59. HSQC spectrum of **9**. 34
Figure S60. HMBC spectrum of **9**. 34
Figure S61. ^{1}H-^{1}H COSY spectrum of **9**. 35
Figure S62. ROESY spectrum of **9**. 35
Figure S63. HRESIMS of **9**. 36
Figure S64. ^{1}H NMR spectrum of **10** (600 MHz, $CDCl_3$). 37
Figure S65. ^{13}C and DEPT NMR spectra of **10** (150 MHz, $CDCl_3$). 37
Figure S66. HSQC spectrum of **10**. 38
Figure S67. HMBC spectrum of **10**. 38
Figure S68. ^{1}H-^{1}H COSY spectrum of **10**. 39
Figure S69. ROESY spectrum of **10**. 39
Figure S70. HRESIMS of **10**. 40
Figure S71. ^{1}H NMR spectrum of **11** (600 MHz, $CDCl_3$). 41
Figure S72. ^{13}C and DEPT NMR spectra of **11** (150 MHz, $CDCl_3$). 41
Figure S73. HSQC spectrum of **11**. 42
Figure S74. HMBC spectrum of **11**. 42
Figure S75. ^{1}H-^{1}H COSY spectrum of **11**. 43
Figure S76. ROESY spectrum of **11**. 43
Figure S77. HRESIMS of **11**. 44
Figure S78. ^{1}H NMR spectrum of **12** (600 MHz, $CDCl_3$). 45
Figure S79. ^{13}C and DEPT NMR spectra of **12** (150 MHz, $CDCl_3$). 45
Figure S80. HSQC spectrum of **12**. 46
Figure S81. HMBC spectrum of **12**. 46
Figure S82. ^{1}H-^{1}H COSY spectrum of **12**. 47
Figure S83. ROESY spectrum of **12**. 47
Figure S84. HRESIMS of **12**. 48
Figure S85. ^{1}H NMR spectrum of **13** (600 MHz, $CDCl_3$). 49
Figure S86. ^{13}C and DEPT NMR spectra of **13** (150 MHz, $CDCl_3$). 49
Figure S87. HSQC spectrum of **13**. 50
Figure S88. HMBC spectrum of **13**. 50
Figure S89. ^{1}H-^{1}H COSY spectrum of **13**. 51
Figure S90. ROESY spectrum of **13**. 51
Figure S91. HRESIMS of **13**. 52
Figure S92. ^{1}H NMR spectrum of **1a** (600 MHz, $CDCl_3$). 53
Figure S93. ^{1}H-^{1}H COSY spectrum of **1a**. 53
Figure S94. ^{1}H NMR spectrum of **1b** (600 MHz, $CDCl_3$). 54
Figure S95. ^{1}H-^{1}H COSY spectrum of **1b**. 54

Figure S96. HRESIMS of **1a**. 55
Figure S97. HRESIMS of **1b**. 56
Figure S98. ^{1}H NMR spectrum of **2a** (600 MHz, $CDCl_3$). 57
Figure S99. ^{1}H-^{1}H COSY spectrum of **2a**. 57
Figure S100. ^{1}H NMR spectrum of **2b** (600 MHz, $CDCl_3$). 58
Figure S101. ^{1}H-^{1}H COSY spectrum of **2b**. 58
Figure S102. HRESIMS of **2a**. 59
Figure S103. HRESIMS of **2b**. 60
Figure S104. ^{1}H NMR spectrum of **3a** (600 MHz, $CDCl_3$). 61
Figure S105. ^{1}H-^{1}H COSY spectrum of **3a**. 61
Figure S106. ^{1}H NMR spectrum of **3b** (600 MHz, $CDCl_3$). 62
Figure S107. ^{1}H-^{1}H COSY spectrum of **3b**. 62
Figure S108. HRESIMS of **3a**. 63
Figure S109. HRESIMS of **3b**. 64
Figure S110. ^{1}H NMR spectrum of **6H** (600 MHz, $CDCl_3$). 65
Figure S111. ^{13}C NMR spectrum of **6H** (600 MHz, $CDCl_3$). 65
Figure S112. ^{1}H NMR spectrum of **6Ha** (600 MHz, $CDCl_3$). 66
Figure S113. ^{1}H-^{1}H COSY spectrum of **6Ha**. 66
Figure S114. ^{1}H NMR spectrum of **6Hb** (600 MHz, $CDCl_3$). 67
Figure S115. ^{1}H-^{1}H COSY spectrum of **6Hb**. 67
Figure S116. HRESIMS of **6Ha**. 68
Figure S117. HRESIMS of **6Hb**. 69
2. Spectroscopic data of compound 6. 70
Figure S118 The key HMBC and ^{1}H-^{1}H COSY correlations of **6**. 70
3. Biological assays 71
3.1 Cytotoxicity assays 71
3.2 α-Glucosidase inhibition assays 71
3.3 Protein tyrosine phosphate 1 β (PTP1B) inhibition assays 71
3.2 Dipeptidyl peptidase 4 (DDP4) inhibition assays 71
3.3 Angiotensin converting enzyme 2 (ACE2) inhibition assays 71
References: **Error! Bookmark not defined.**

1. Supplementary Figures

Figure S1. ^{1}H NMR spectrum of **1** (600 MHz, CD_3OD).

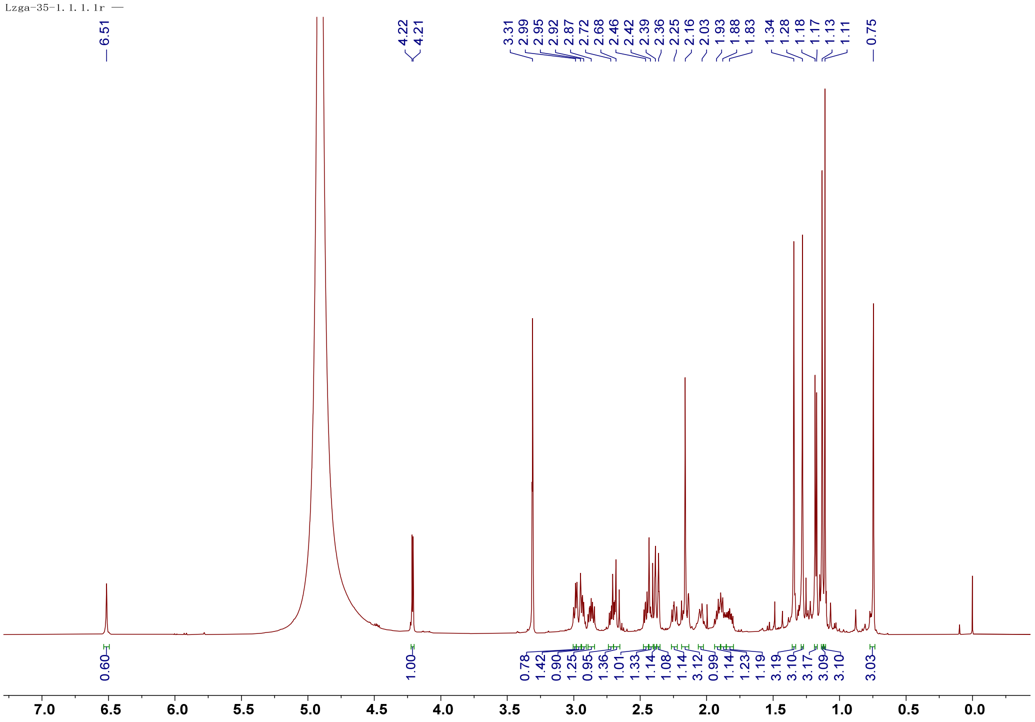

Figure S2. ^{13}C and DEPT NMR spectra of **1** (150 MHz, CD_3OD).

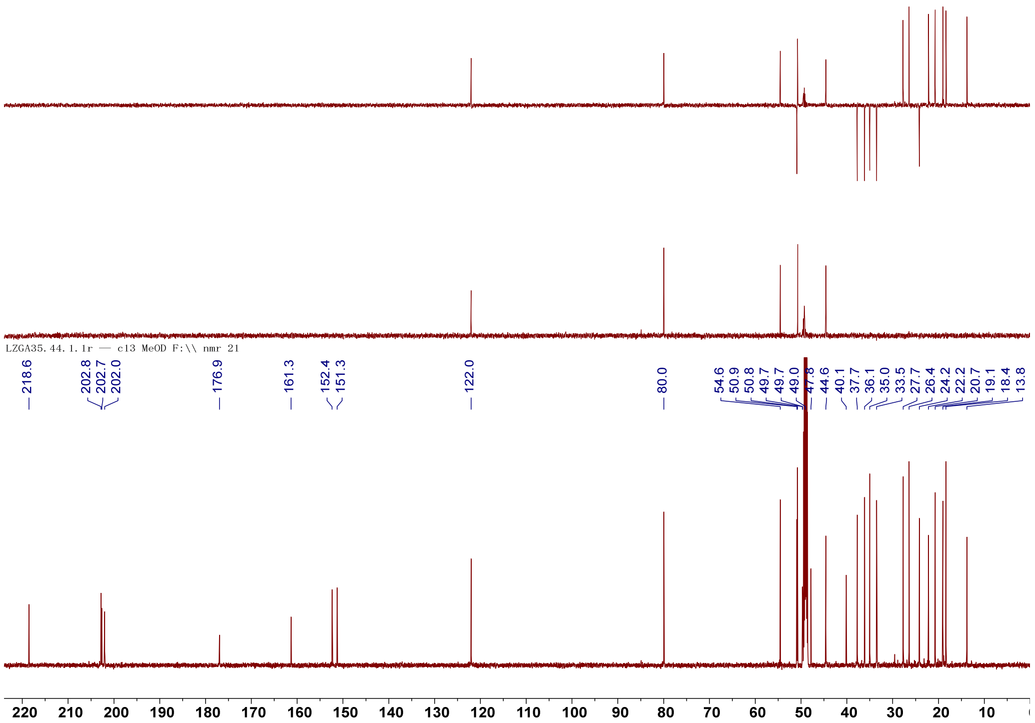

Figure S3. HSQC spectrum of **1**.

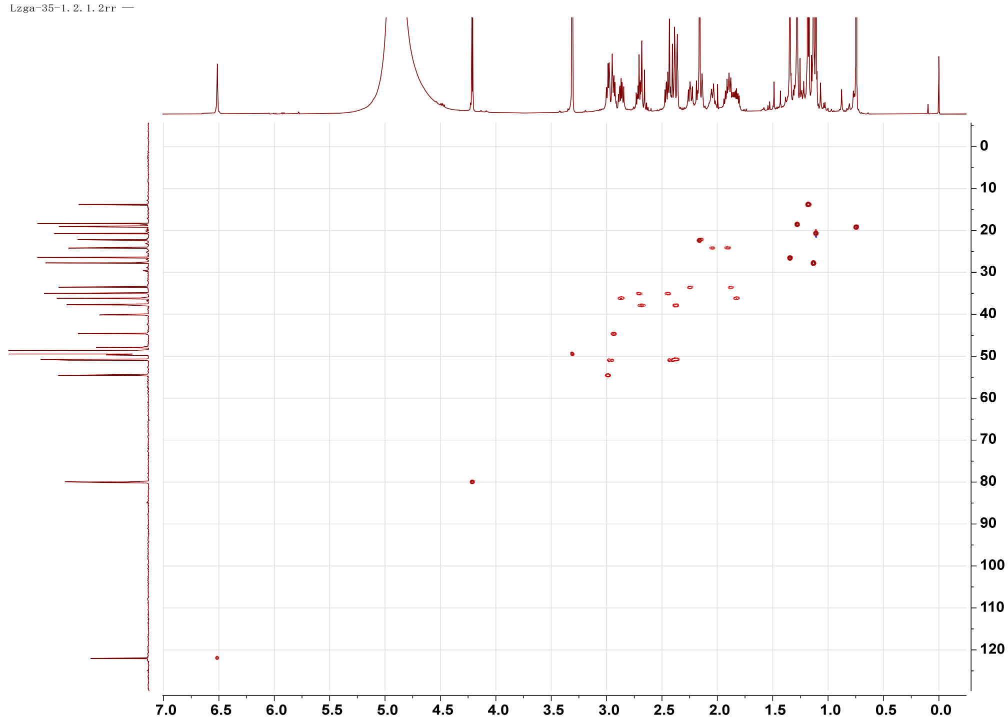

Figure S4. HMBC spectrum of **1**.

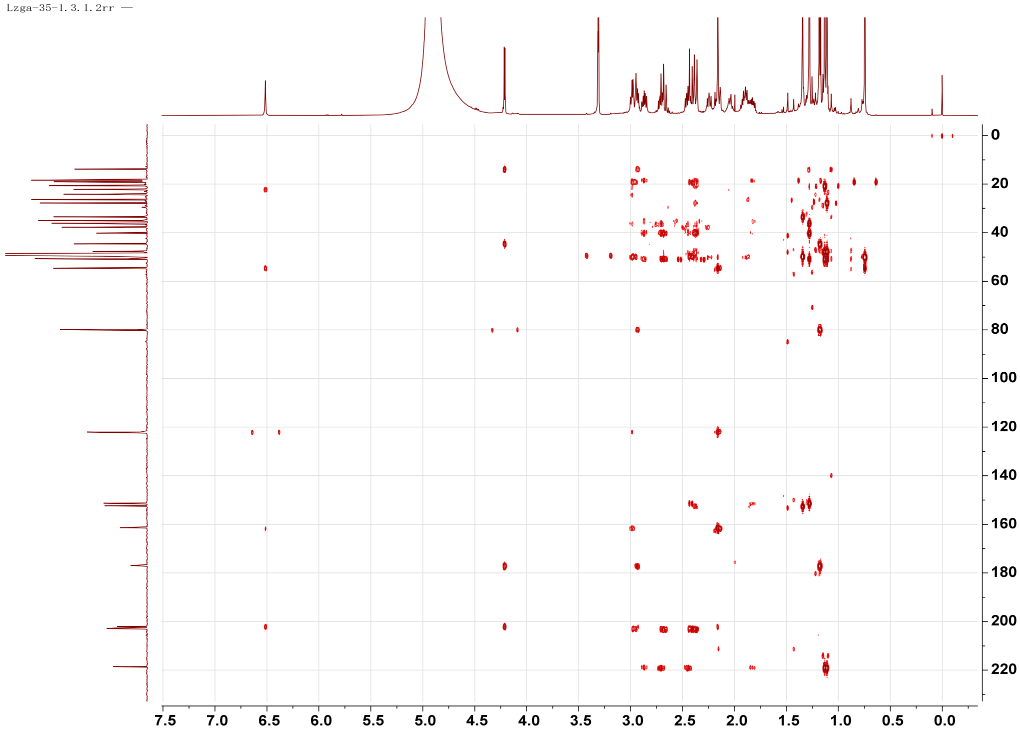

Figure S5. ^{1}H-^{1}H COSY spectrum of **1**.

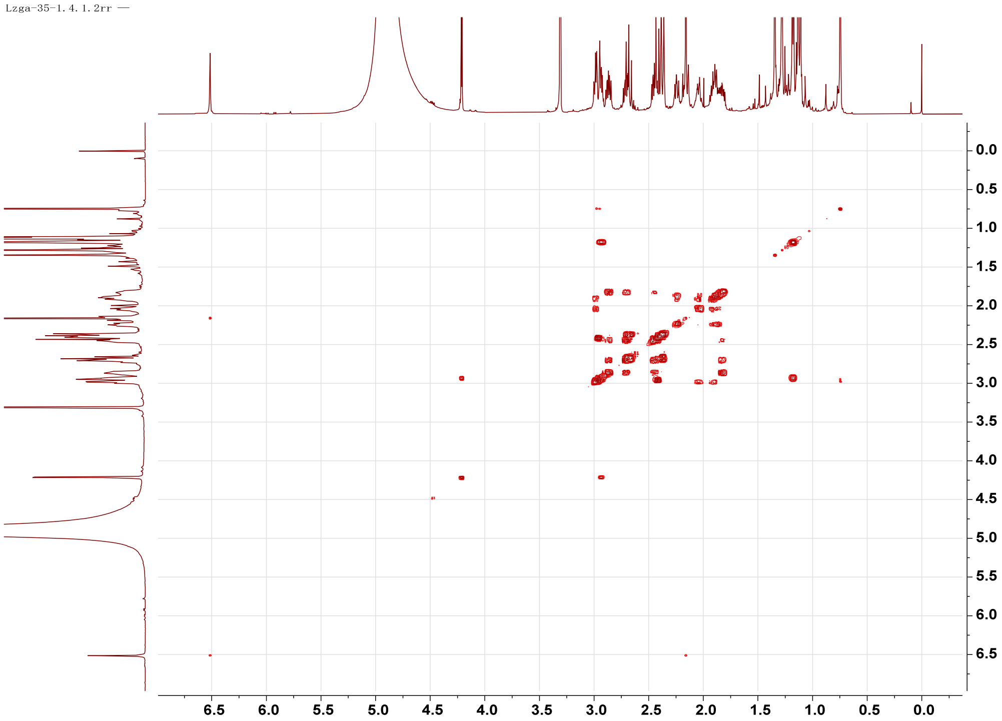

Figure S6. ROESY spectrum of **1**.

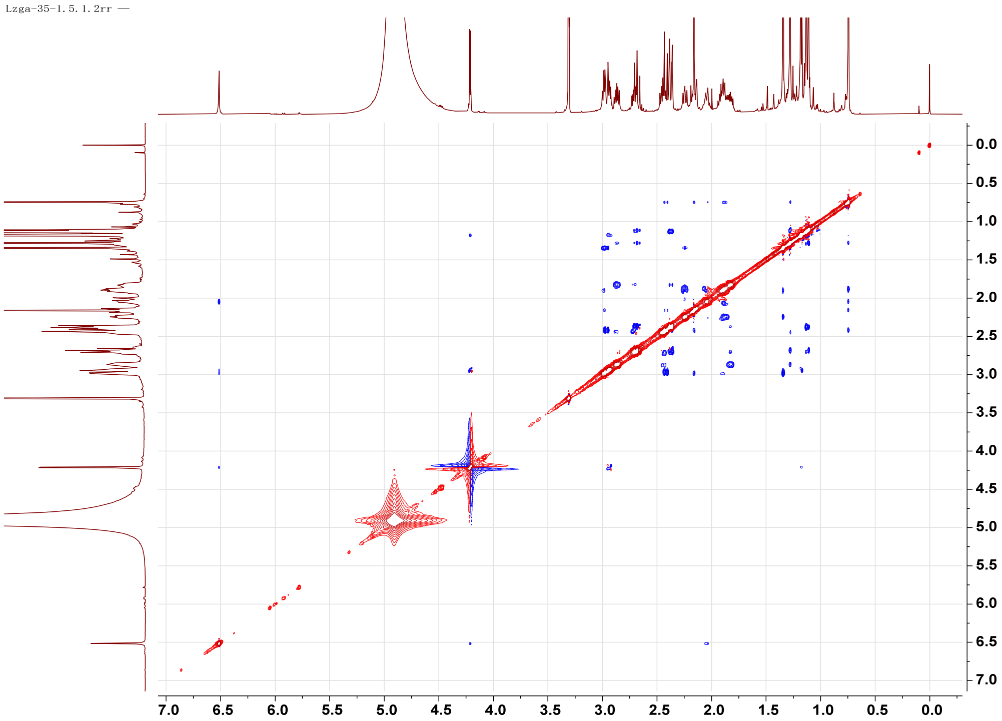

Figure S7. HRESIMS of **1**.

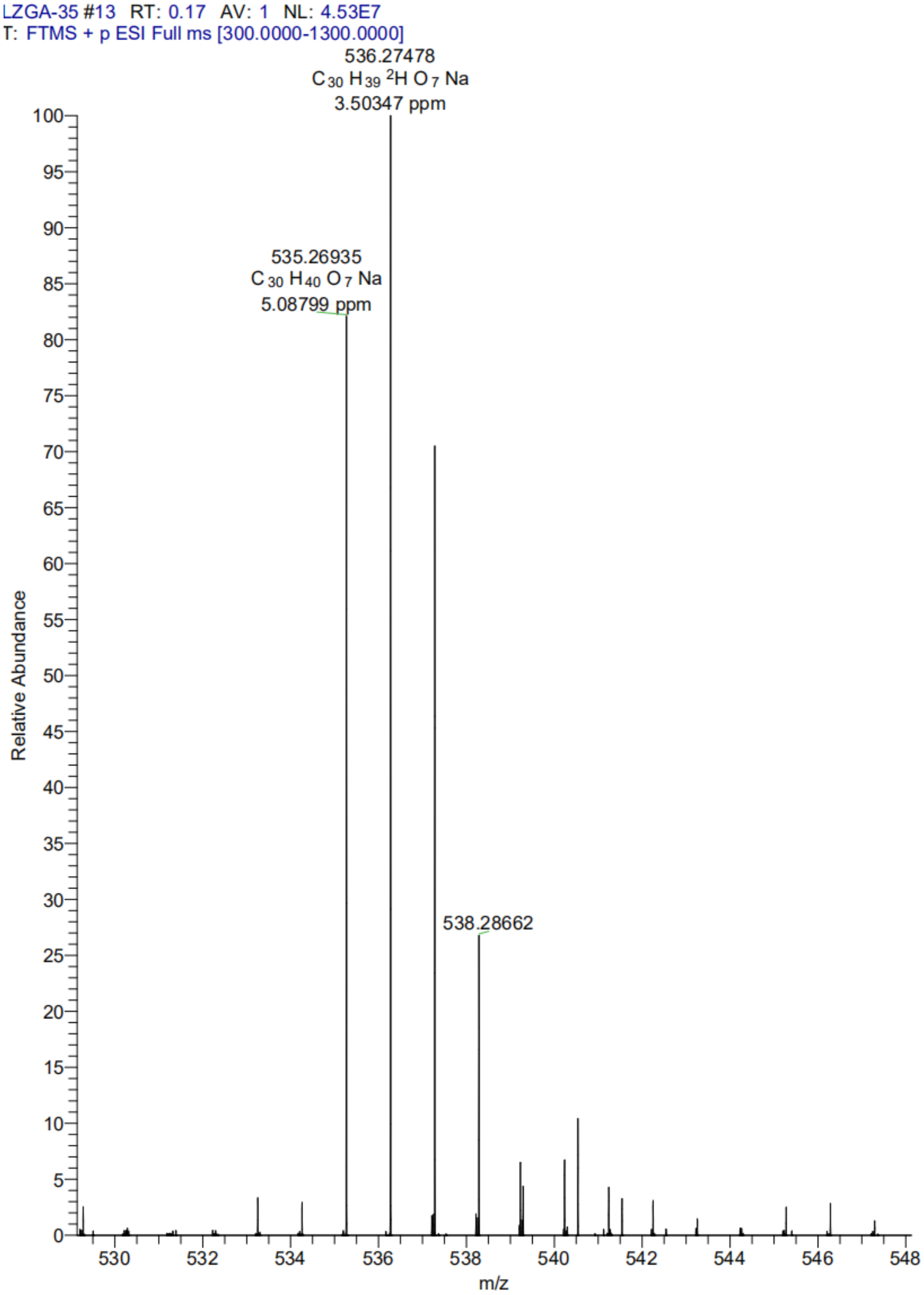

Figure S8. ^{1}H NMR spectrum of **2** (600 MHz, $CDCl_3$).

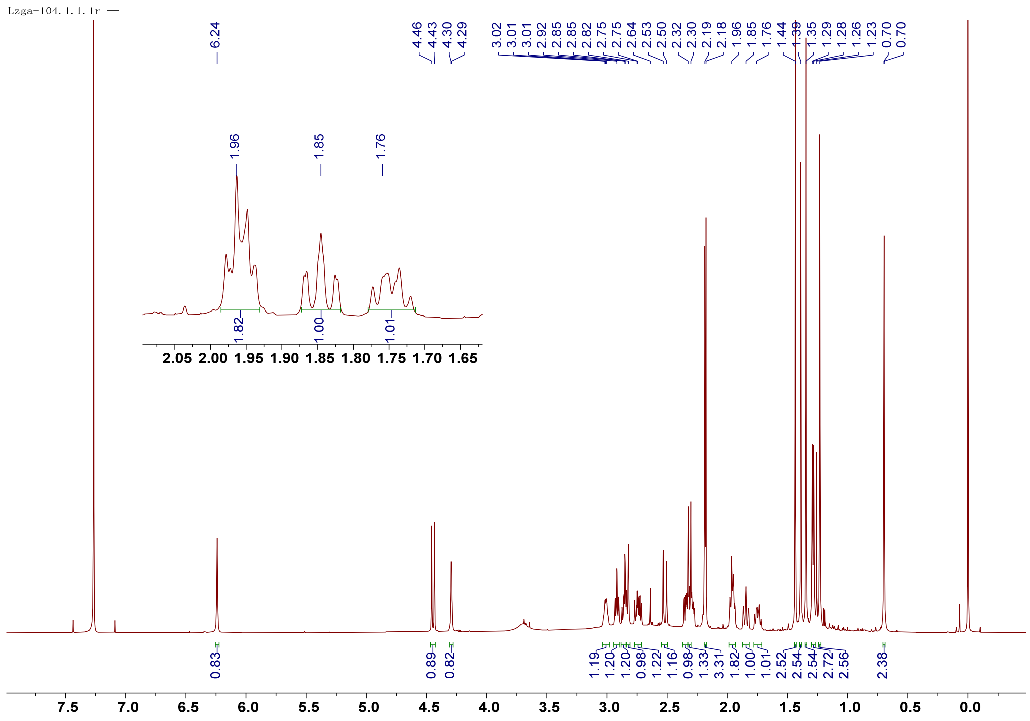

Figure S9. ^{13}C and DEPT NMR spectra of **2** (150 MHz, $CDCl_3$).

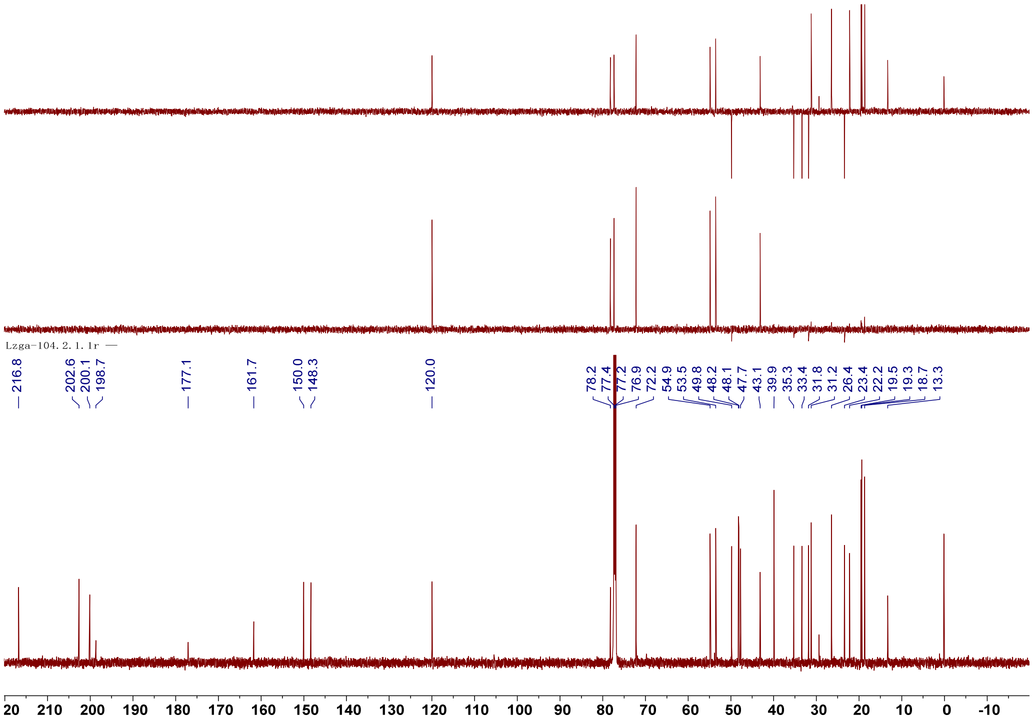

Figure S10. HSQC spectrum of **2**.

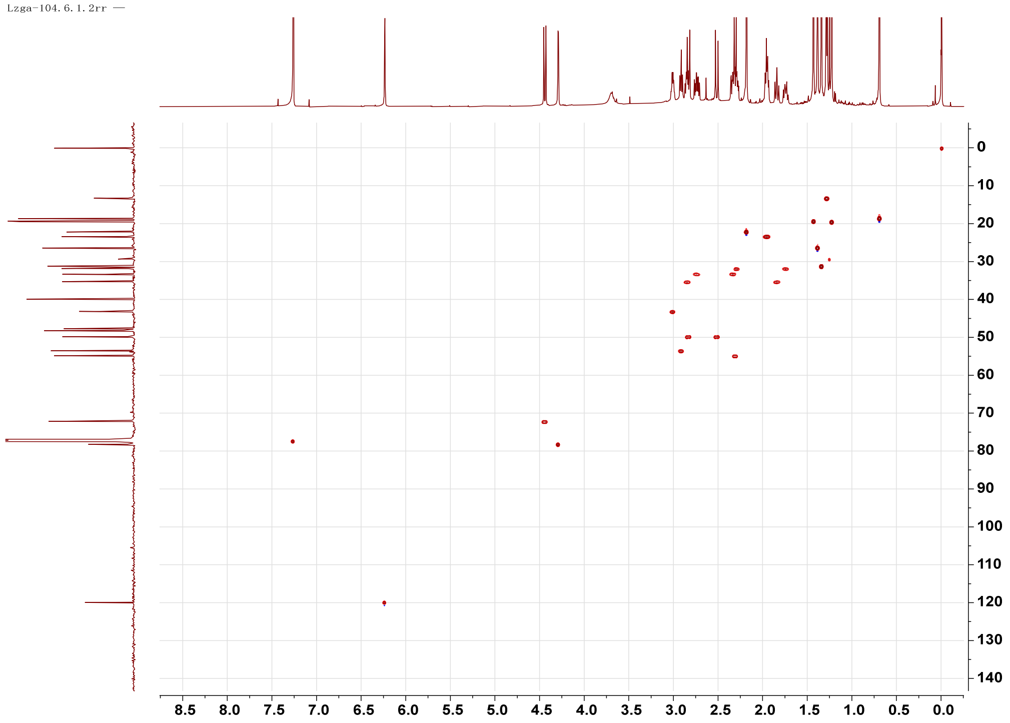

Figure S11. HMBC spectrum of **2**.

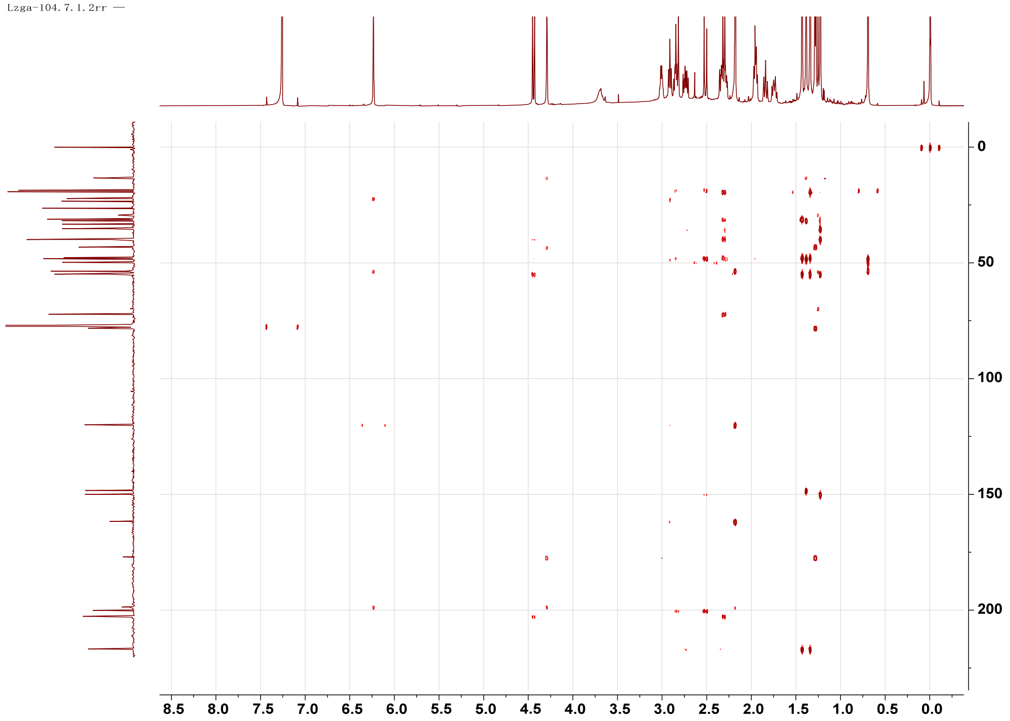

Figure S12. ^{1}H-^{1}H COSY spectrum of **2**.

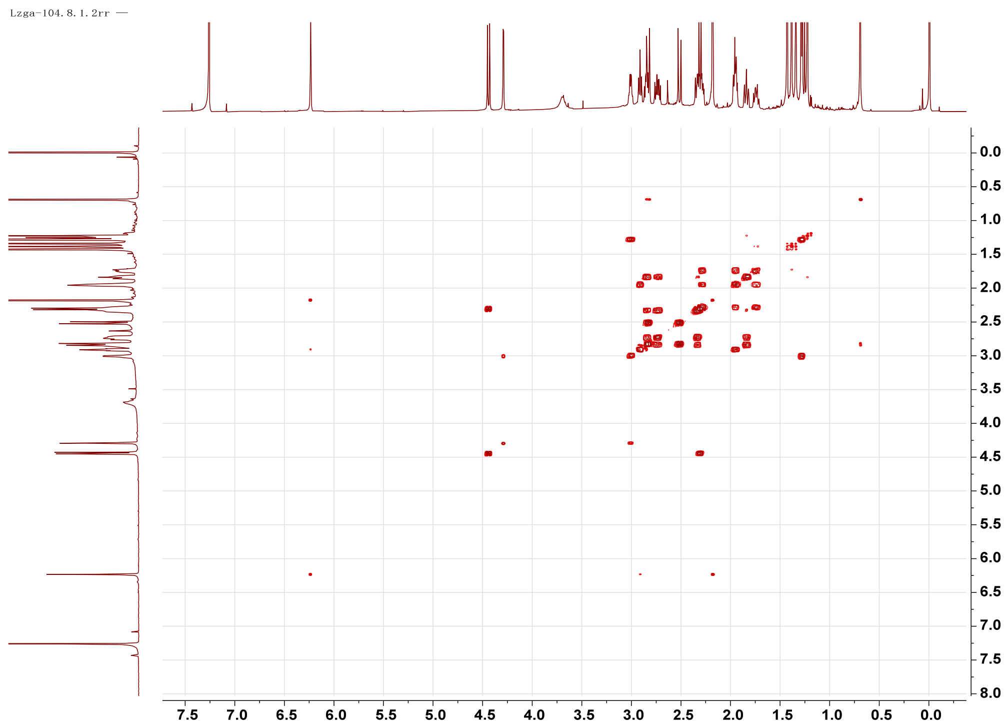

Figure S13. ROESY spectrum of **2**.

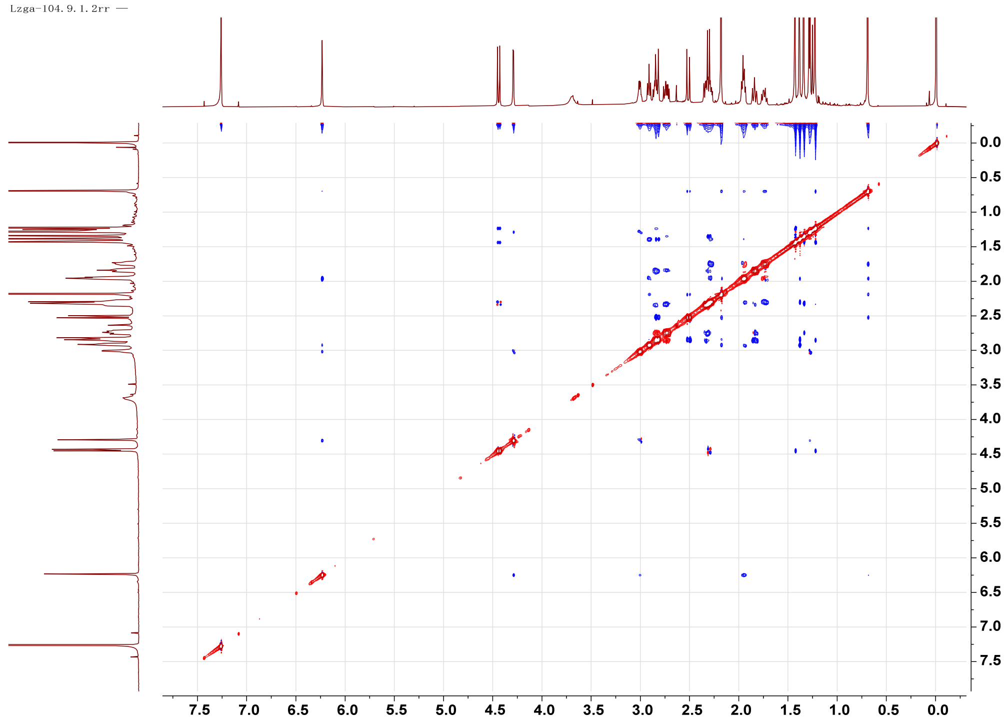

Figure S14. HRESIMS of **2**.

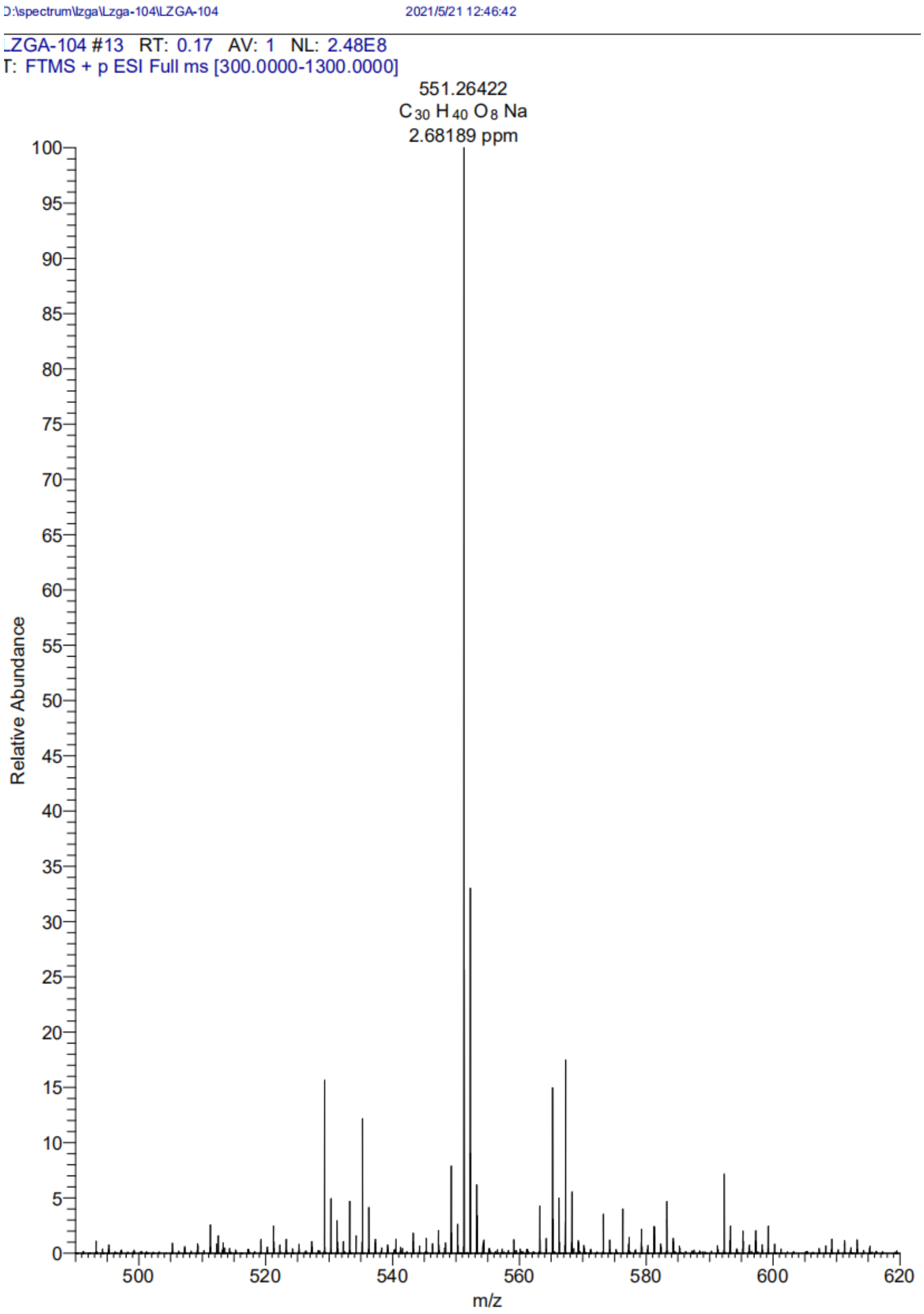

Figure S15. ^{1}H NMR spectrum of **3** (500 MHz, $CDCl_3$).

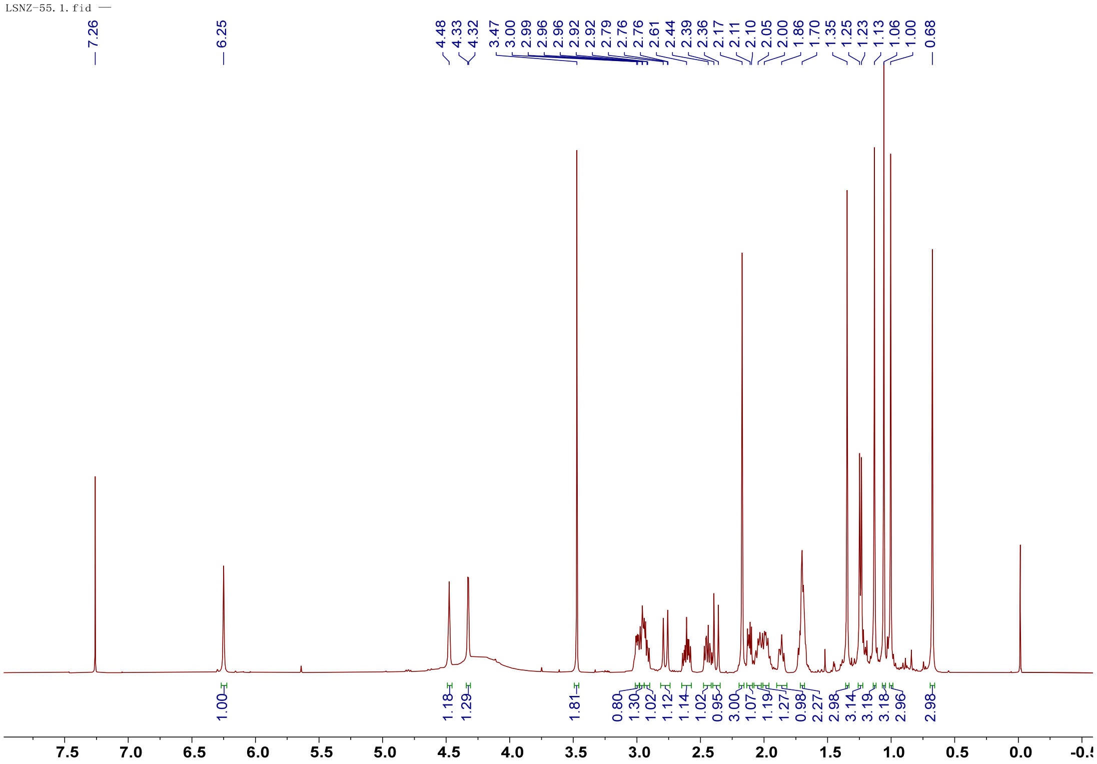

Figure S16. ^{13}C and DEPT NMR spectra of **3** (125 MHz, $CDCl_3$).

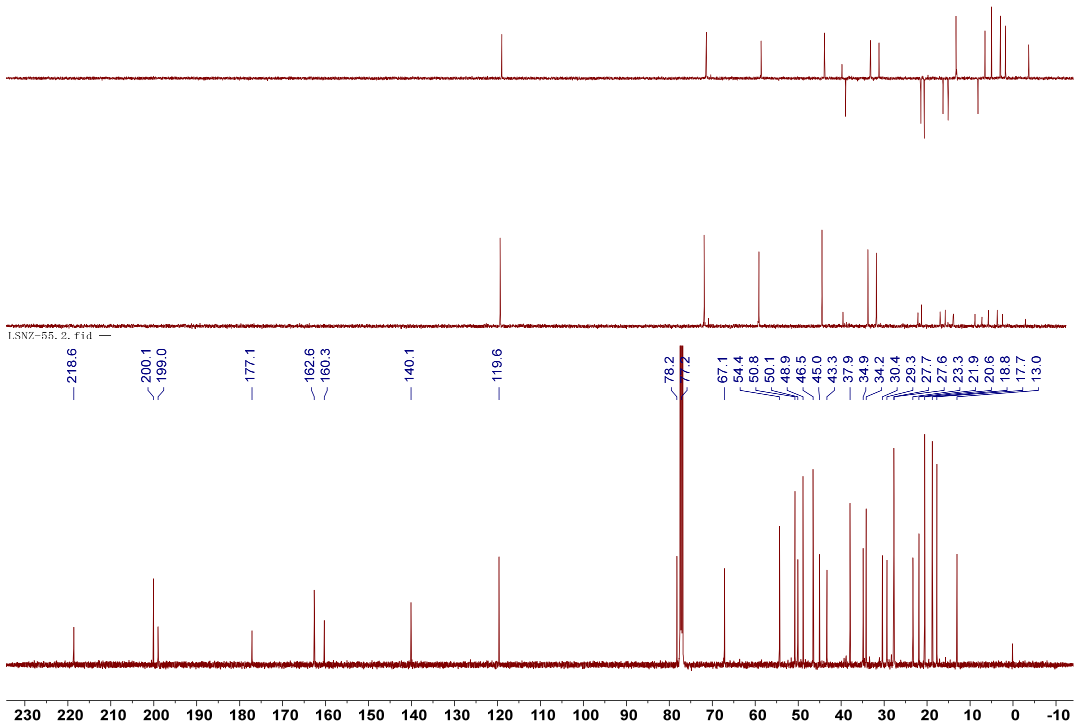

Figure S17. HSQC spectrum of **3**.

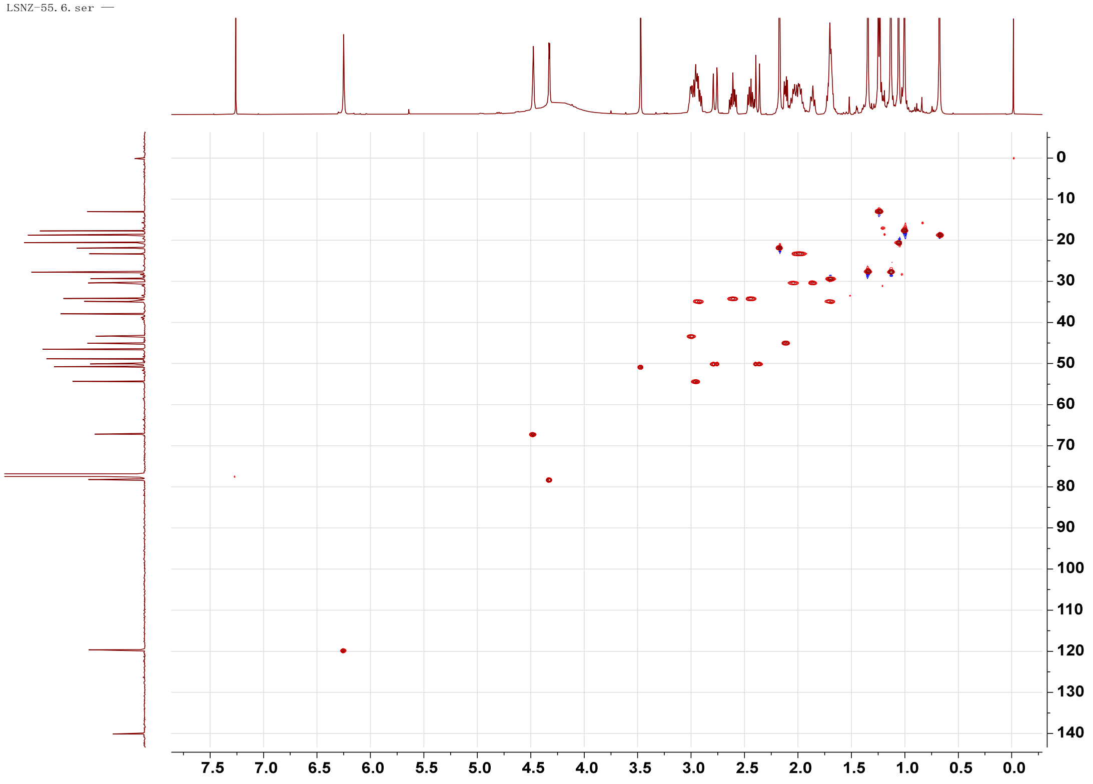

Figure S18. HMBC spectrum of **3**.

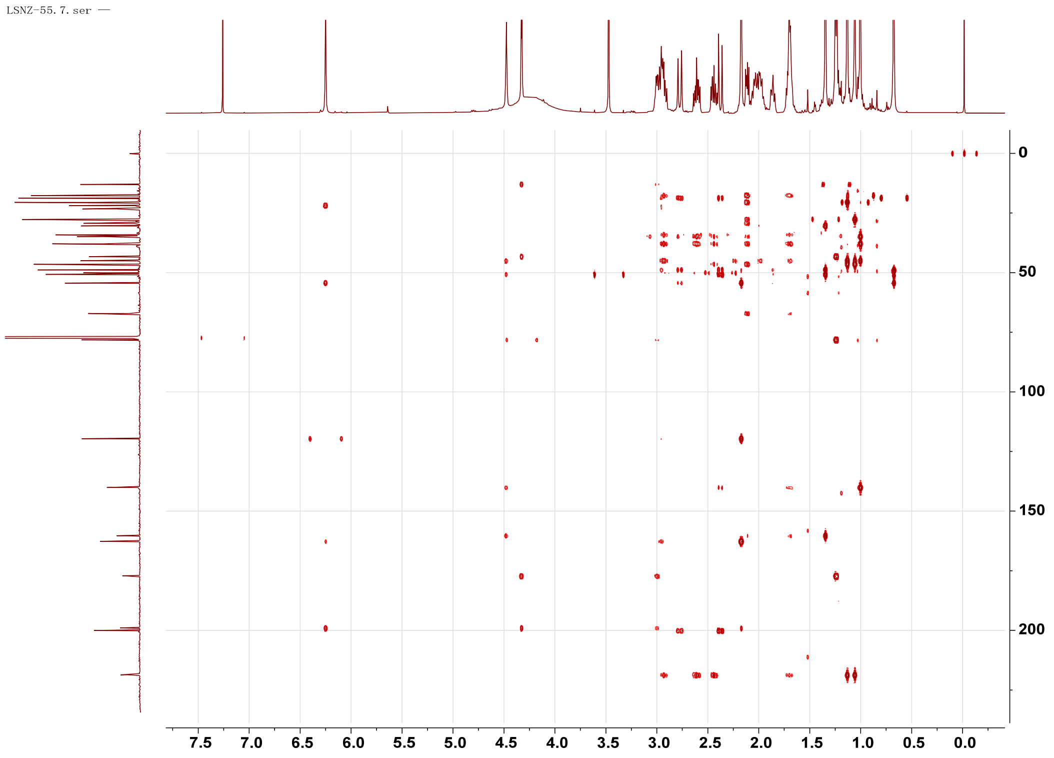

Figure S19. ^{1}H-^{1}H COSY spectrum of **3**.

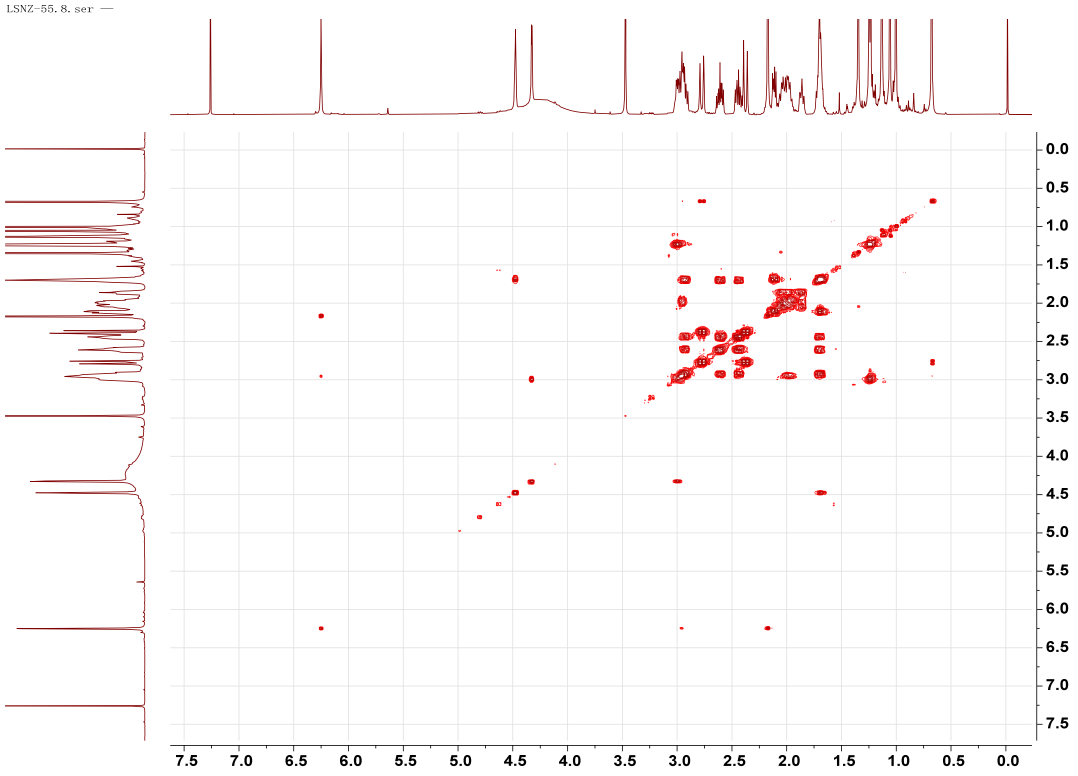

Figure S20. ROESY spectrum of **3**.

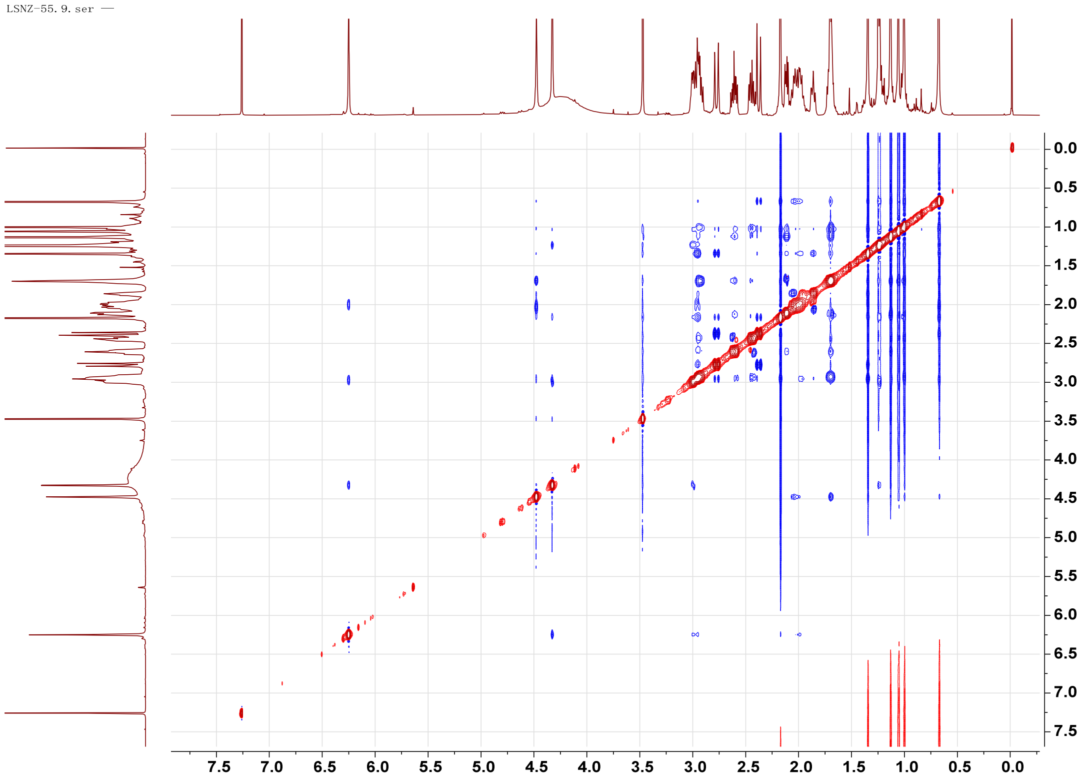

Figure S21. HRESIMS of **3**.

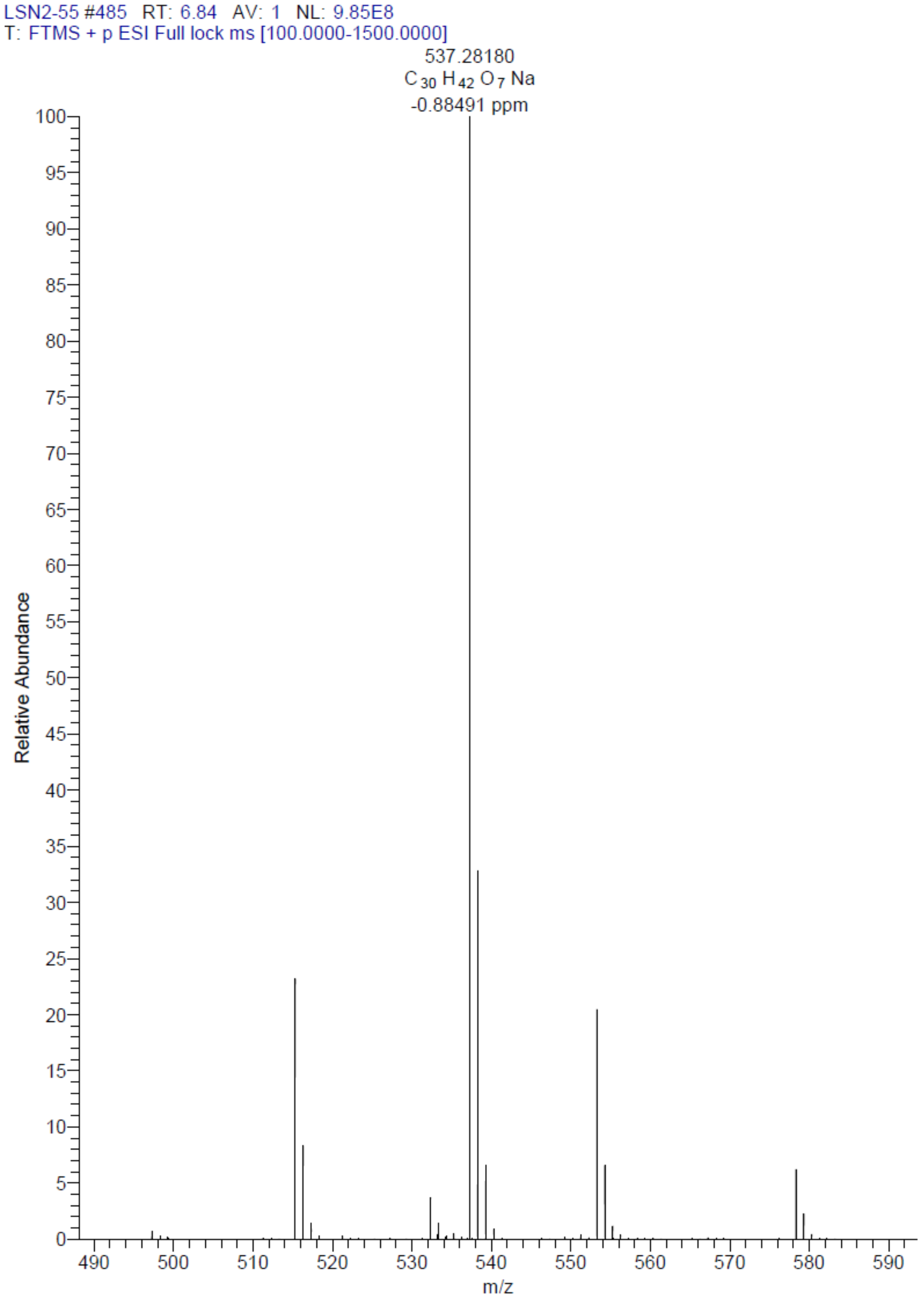

Figure S22. ^{1}H NMR spectrum of **4** (600 MHz, CD_3COCD_3).

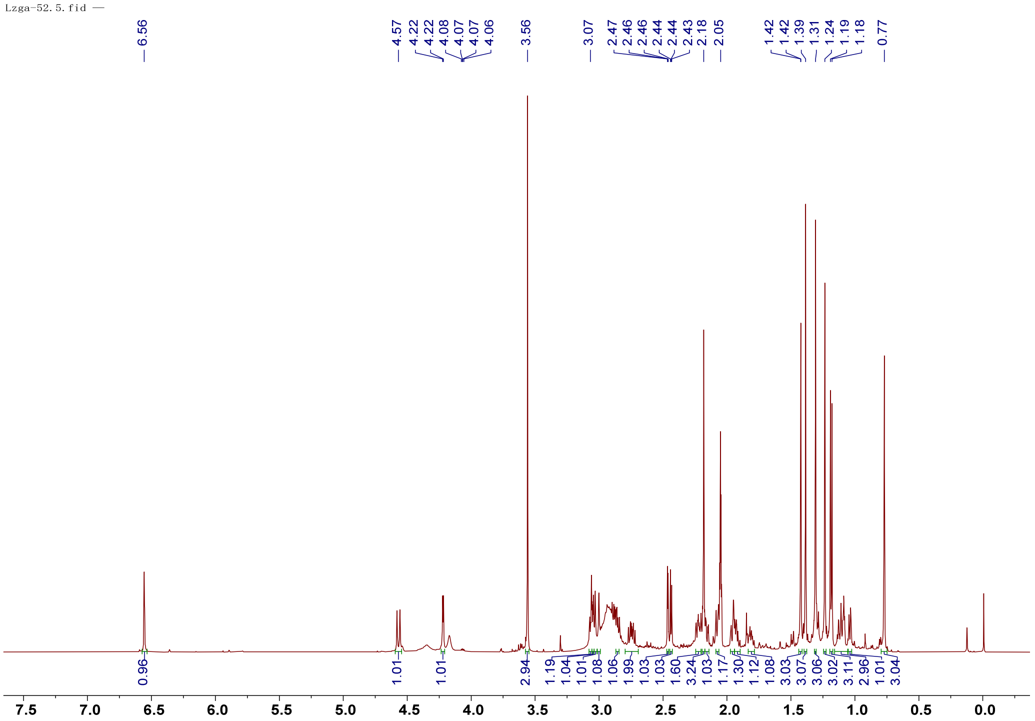

Figure S23. ^{13}C and DEPT NMR spectra of **4** (150 MHz, CD_3COCD_3).

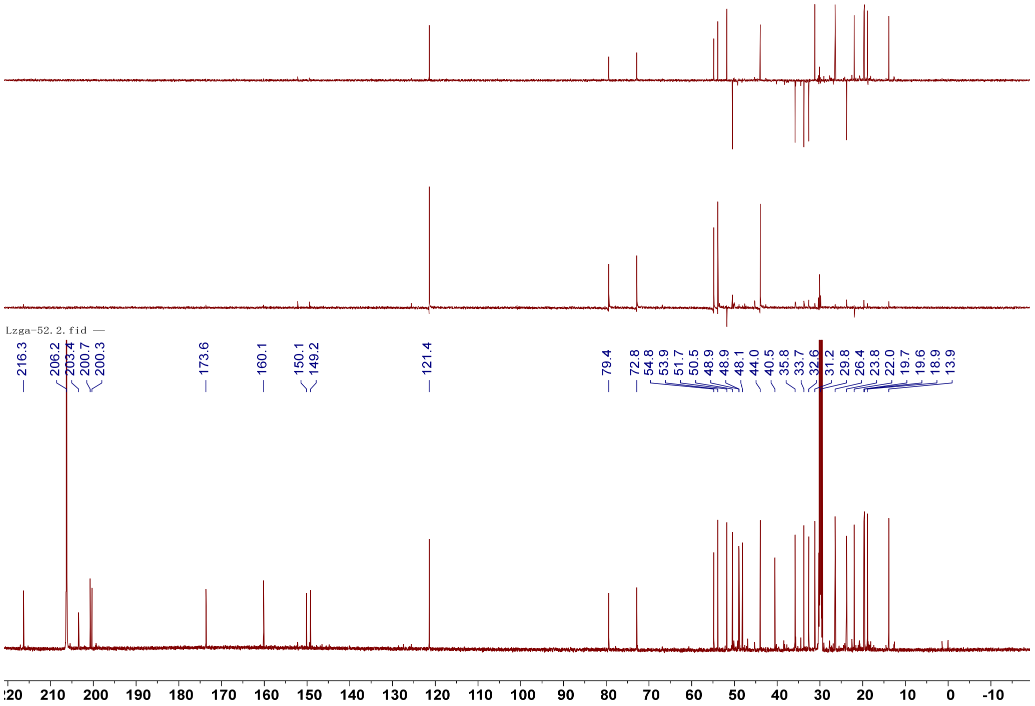

Figure S24. HSQC spectrum of **4**.

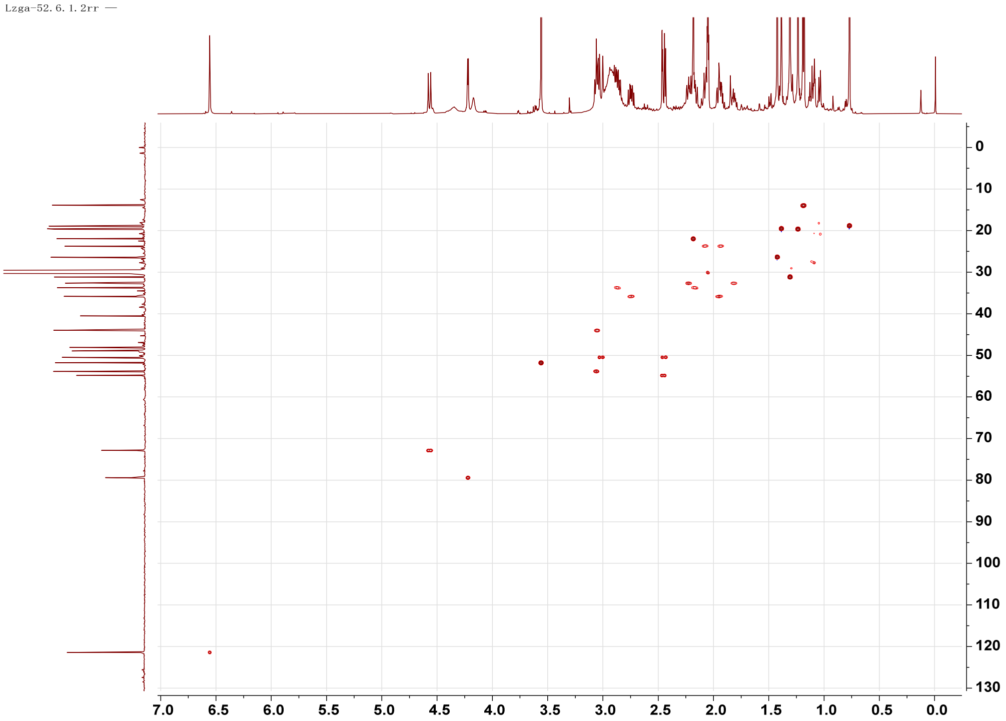

Figure S25. HMBC spectrum of **4**.

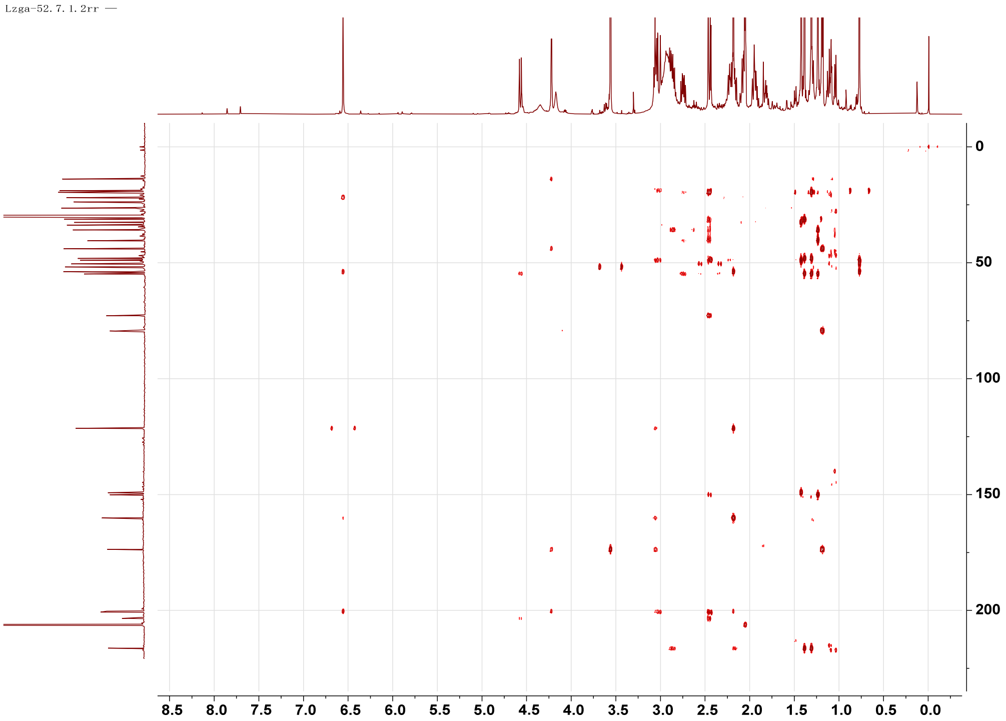

Figure S26. ^{1}H-^{1}H COSY spectrum of **4**.

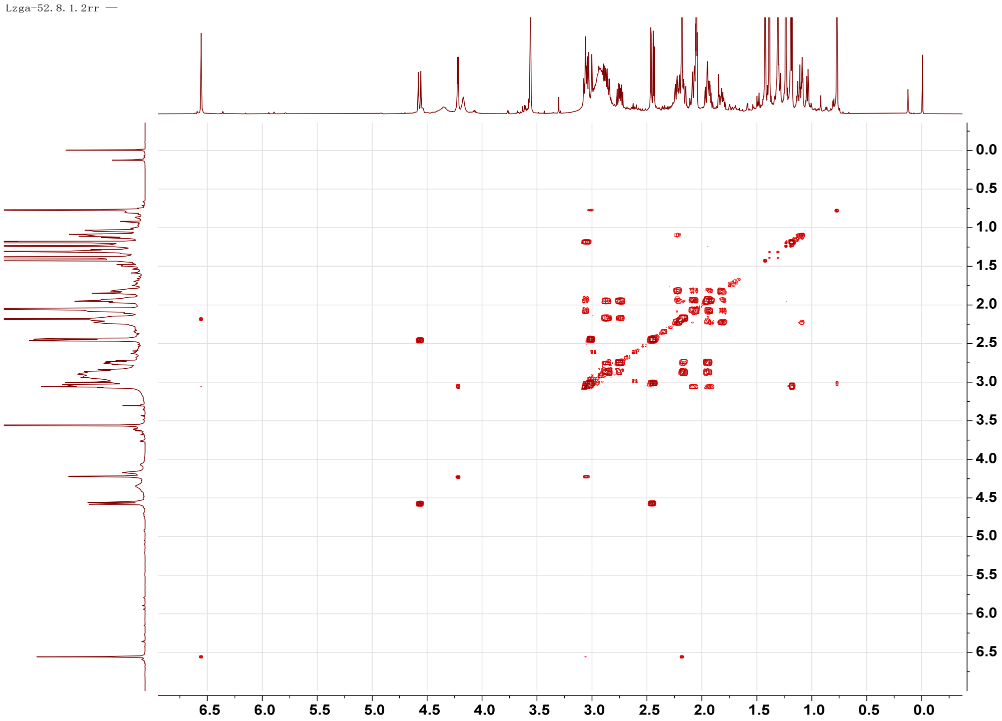

Figure S27. ROESY spectrum of **4**.

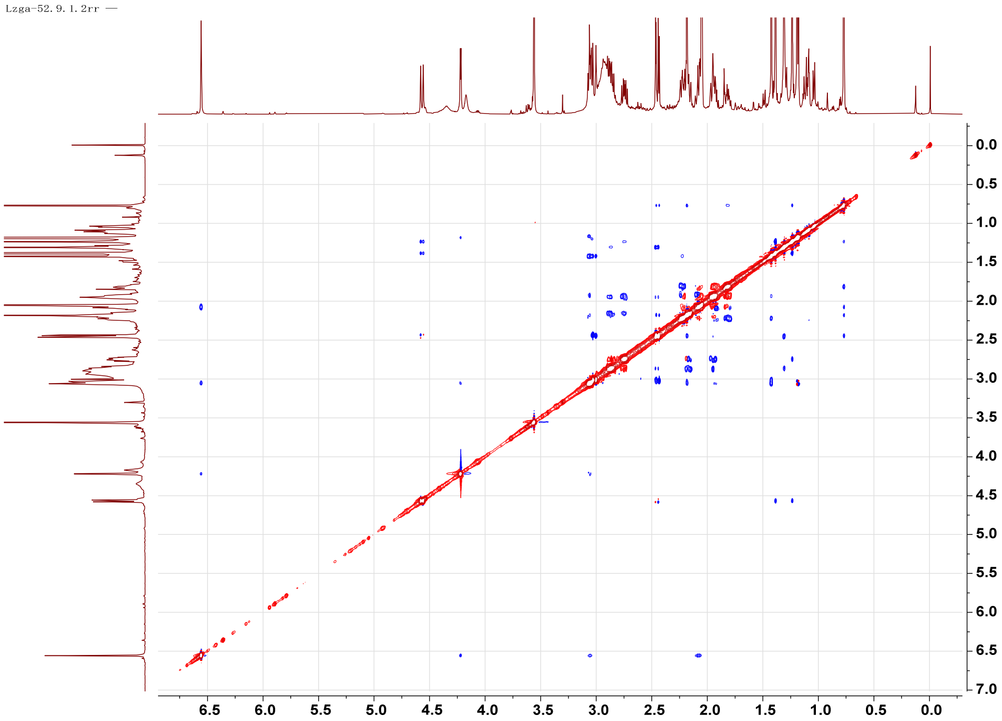

Figure S28. HRESIMS of **4**.

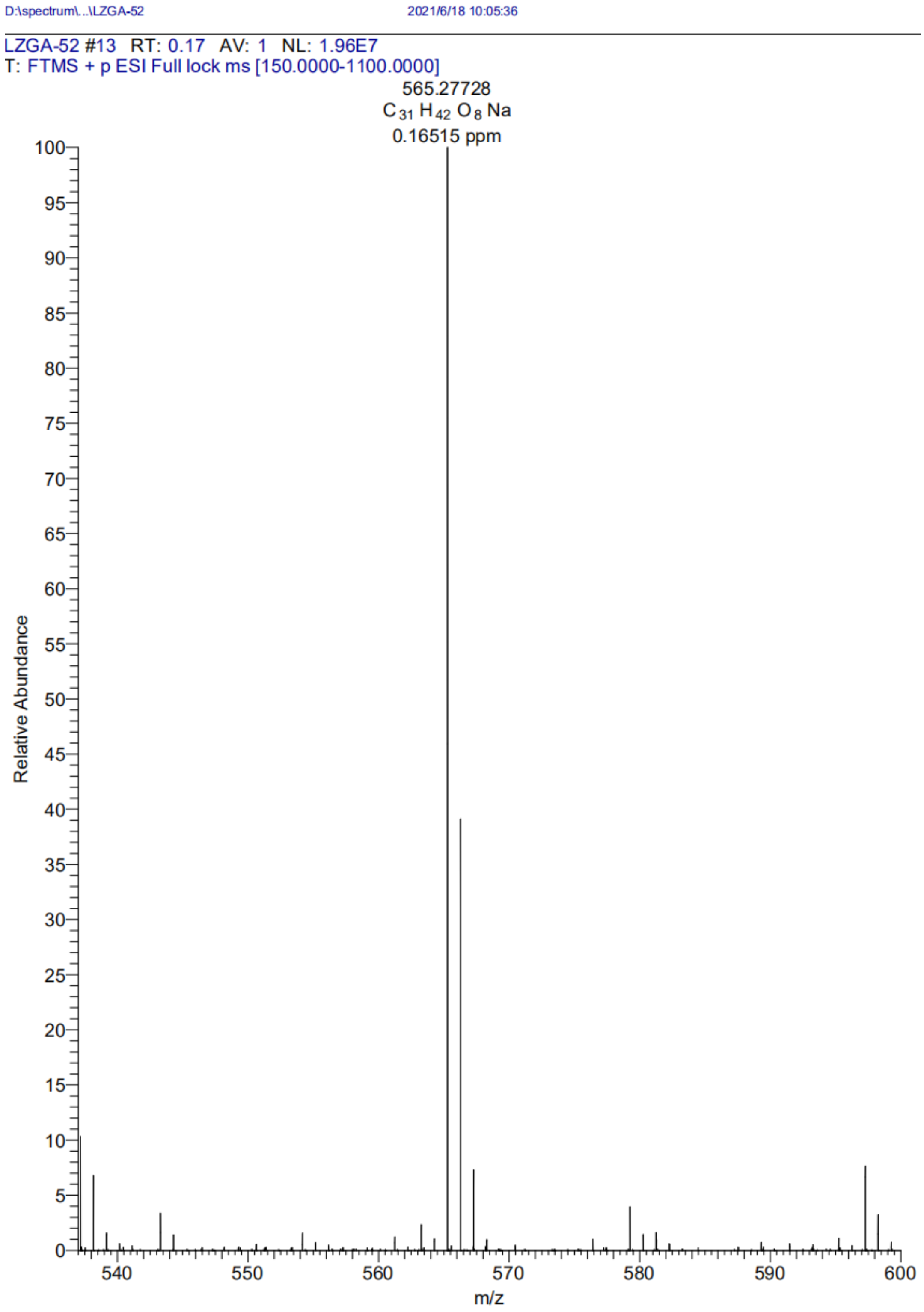

Figure S29. ^{1}H NMR spectrum of **5** (600 MHz, $CDCl_3$).

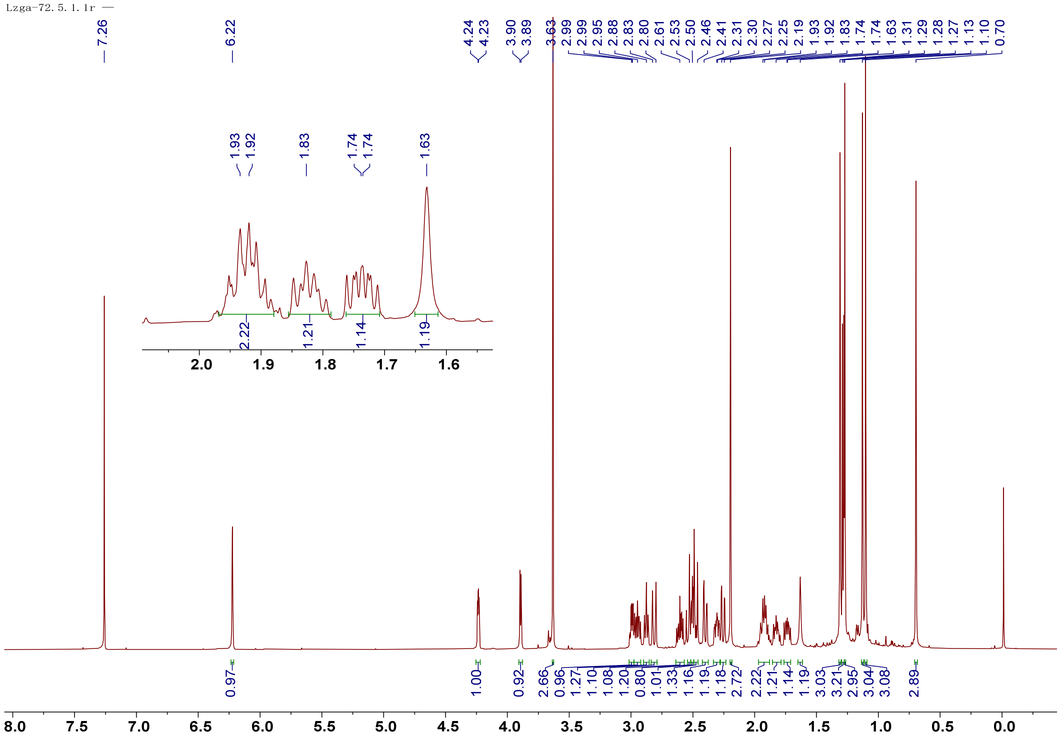

Figure S30. ^{13}C and DEPT NMR spectra of **5** (150 MHz, $CDCl_3$).

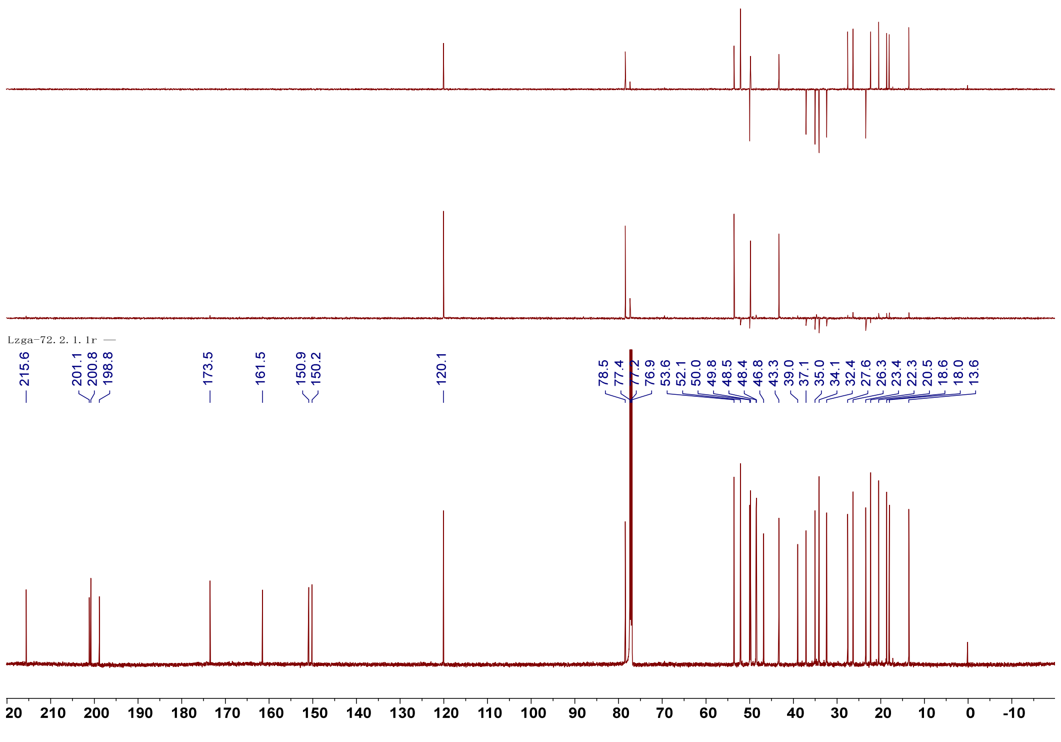

Figure S31. HSQC spectrum of **5**.

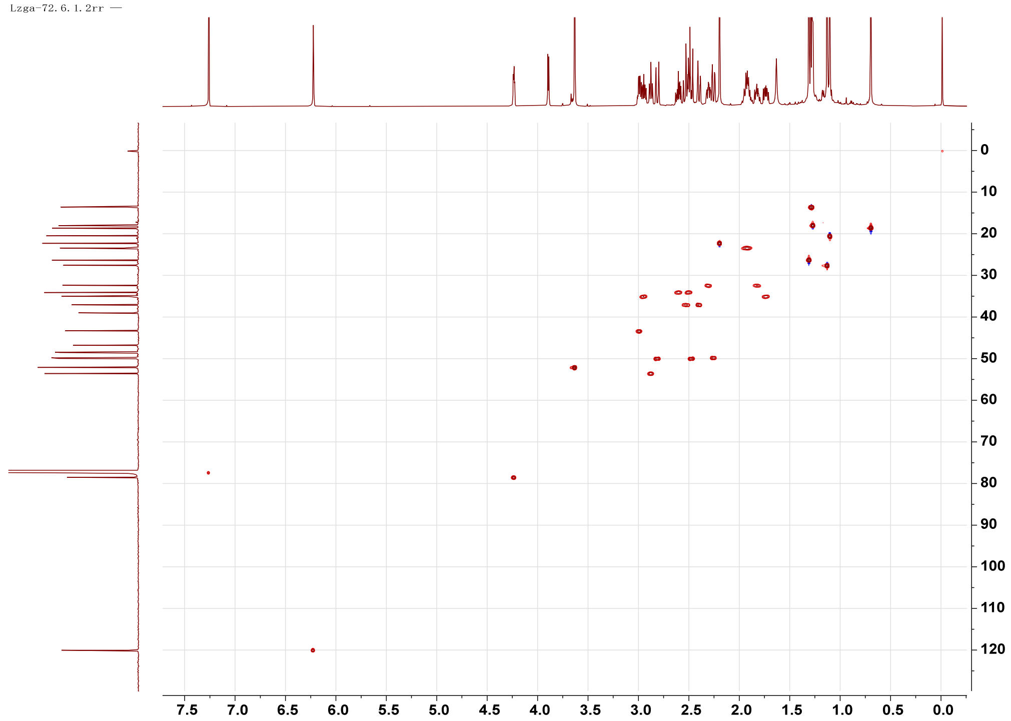

Figure S32. HMBC spectrum of **5**.

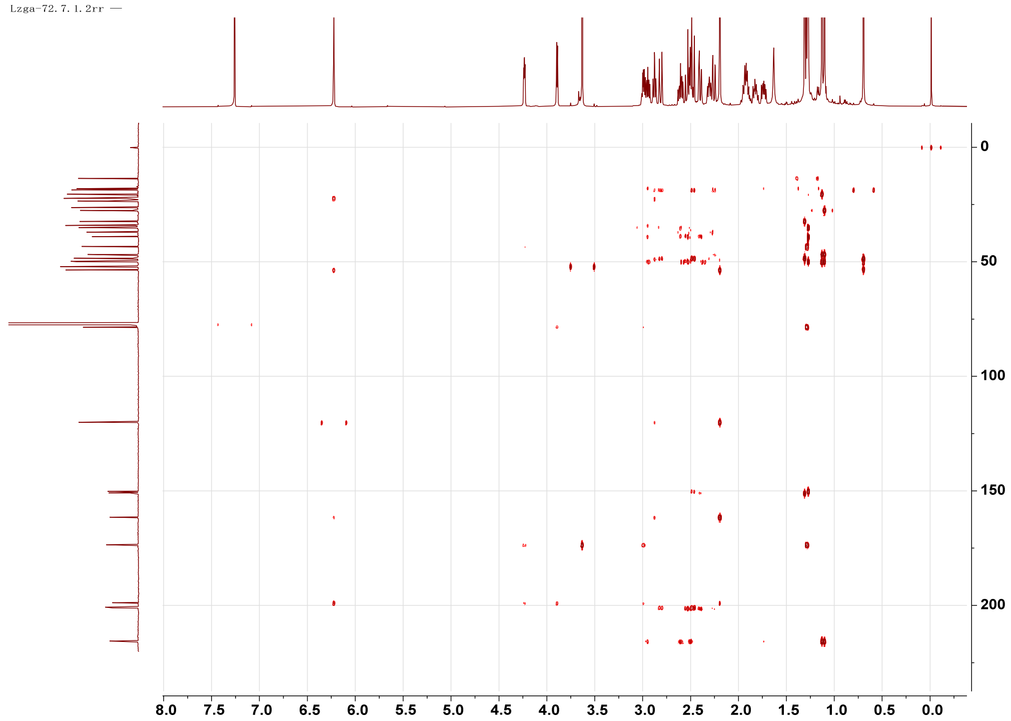

Figure S33. ^{1}H-^{1}H COSY spectrum of **5**.

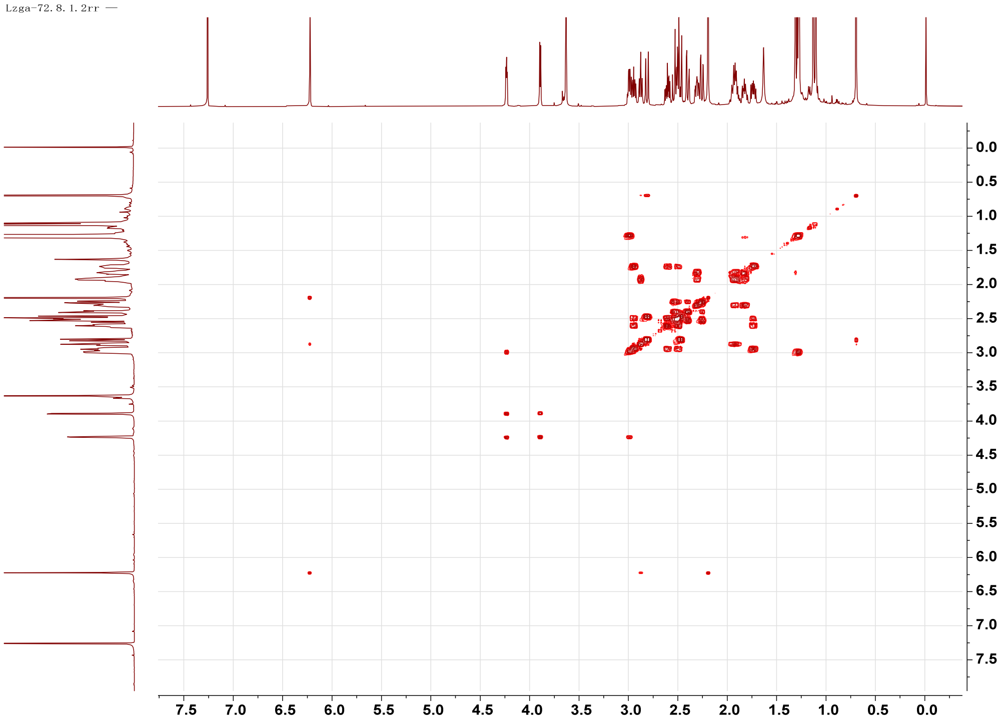

Figure S34. ROESY spectrum of **5**.

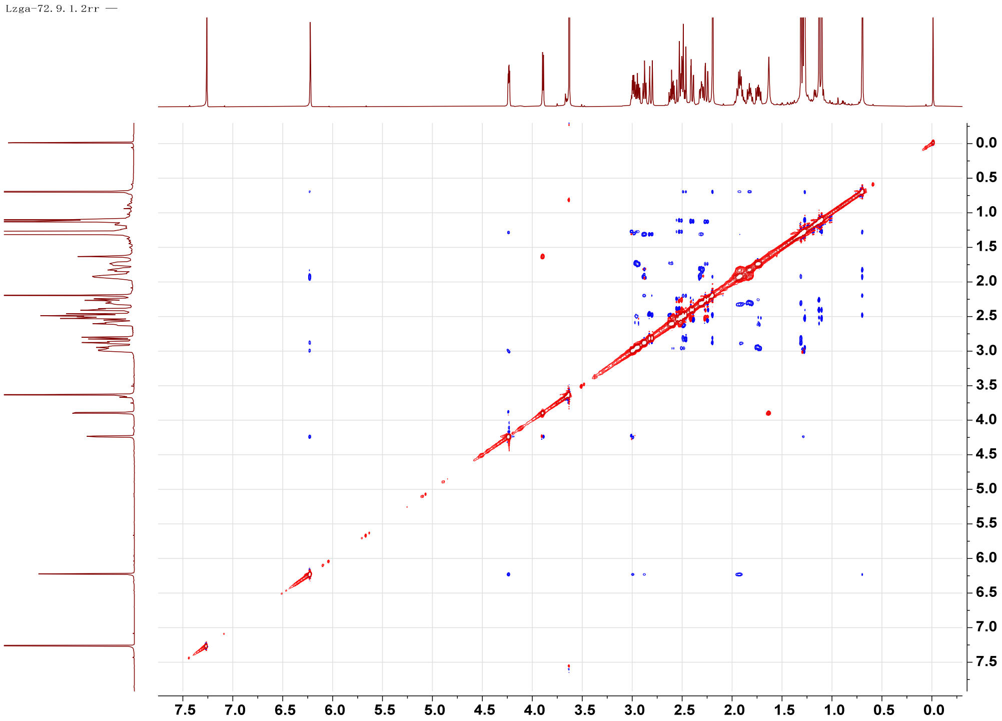

Figure S35. HRESIMS of **5**.

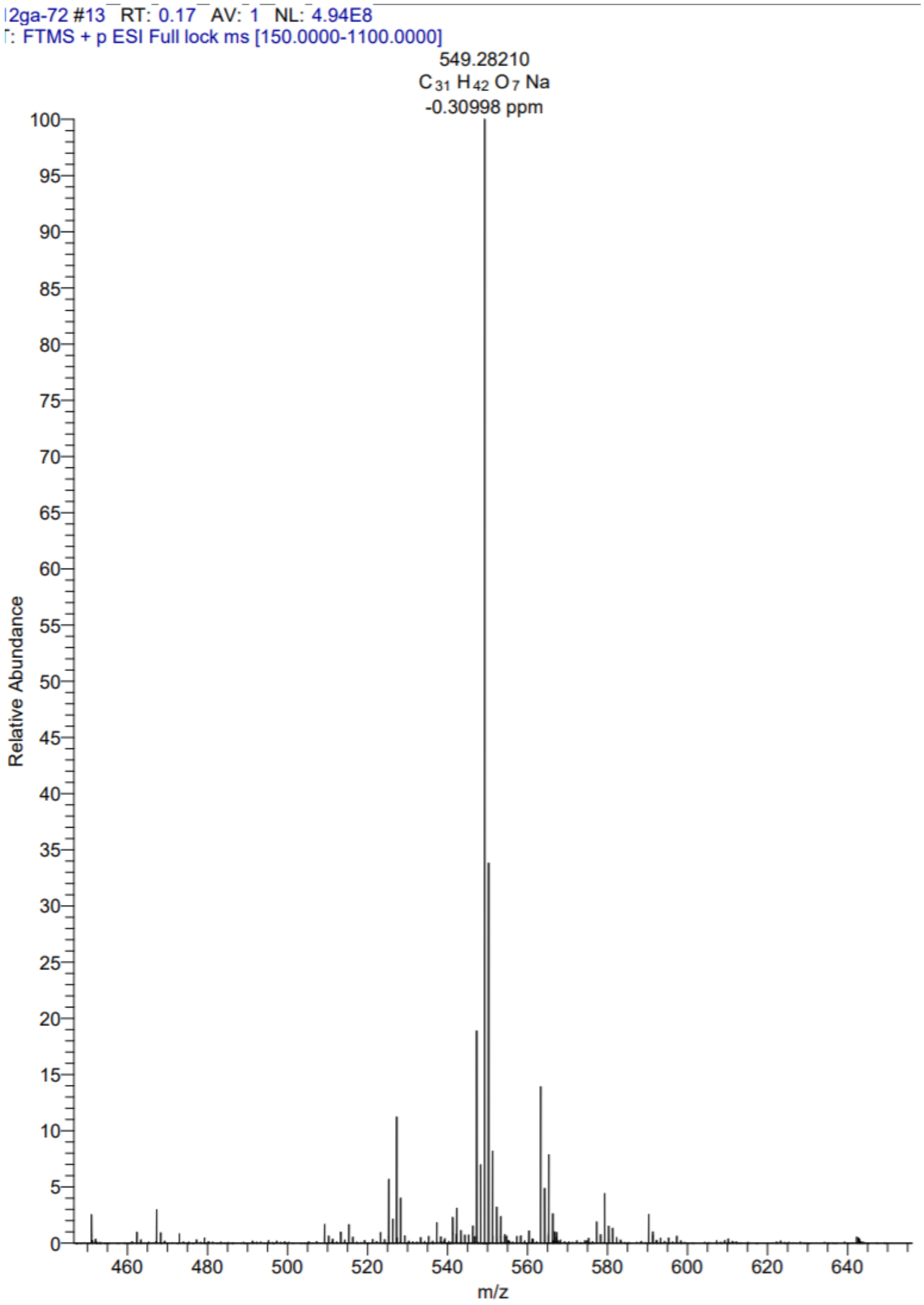

Figure S36. ^{1}H NMR spectrum of **6** (600 MHz, $CDCl_3$).

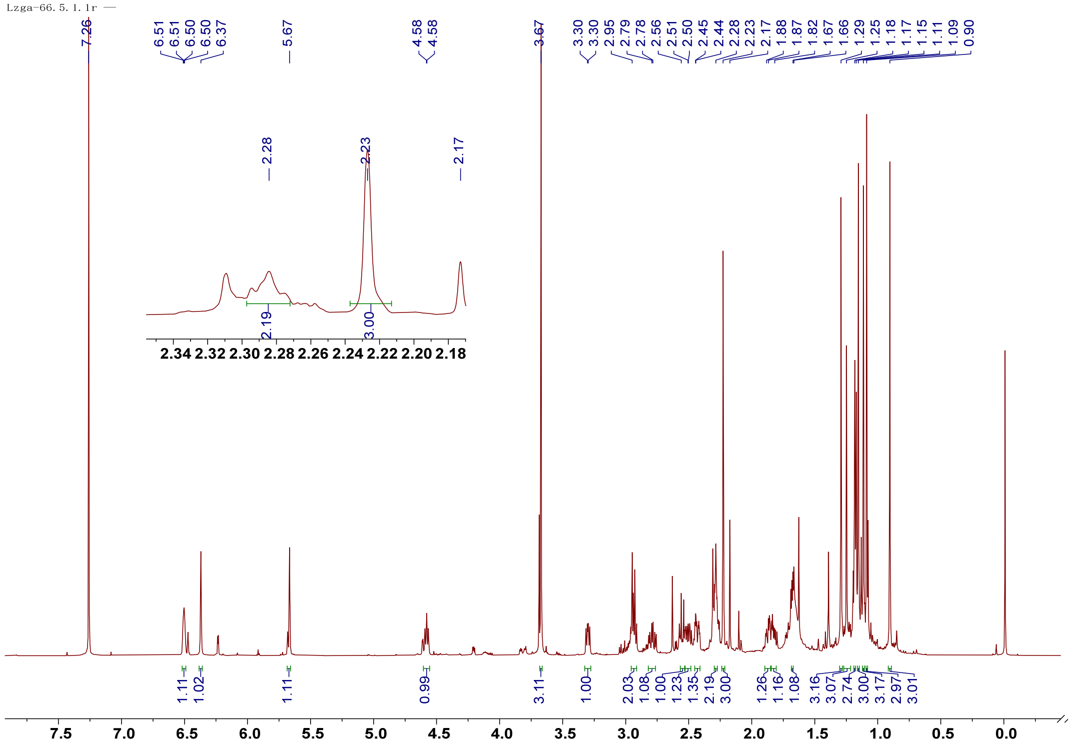

Figure S37. ^{13}C and DEPT NMR spectra of **6** (150 MHz, $CDCl_3$).

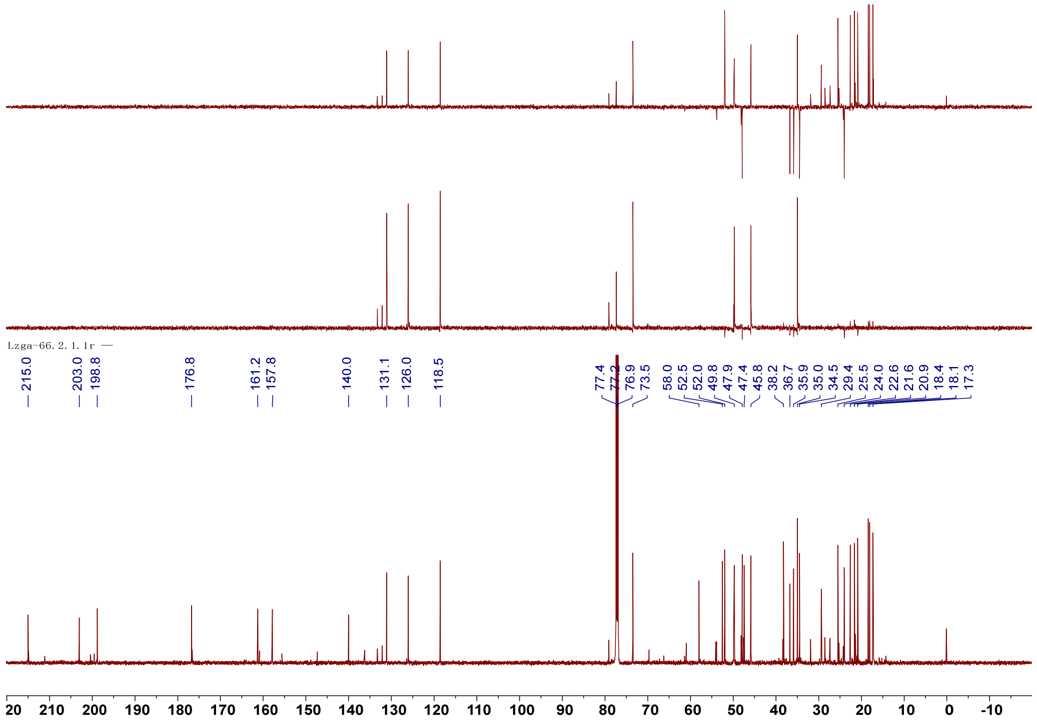

Figure S38. HSQC spectrum of **6**.

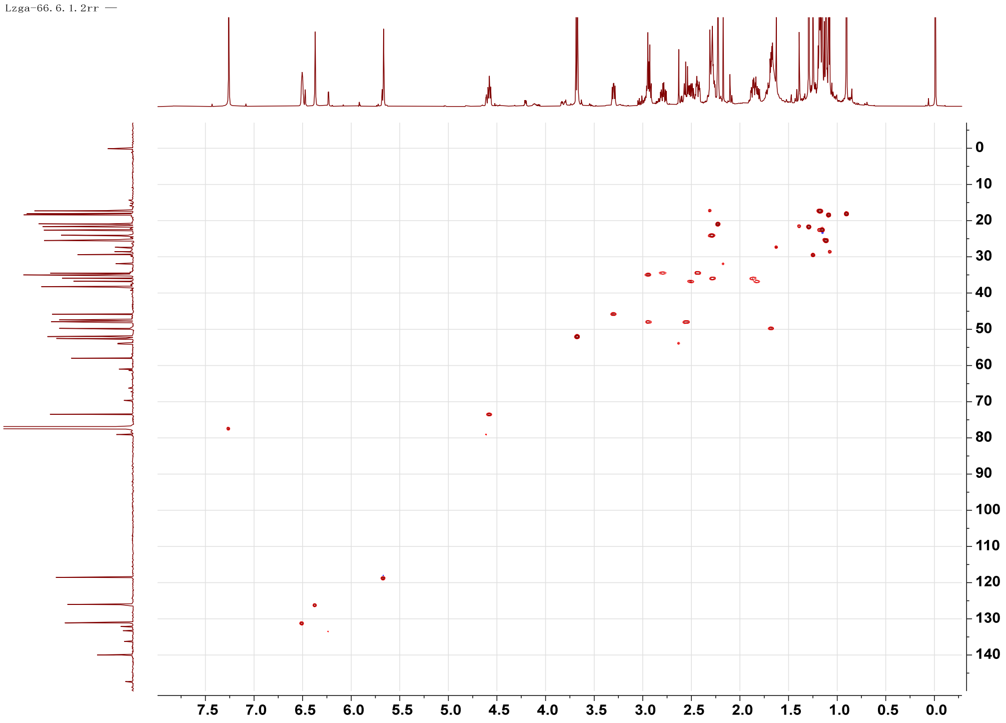

Figure S39. HMBC spectrum of **6**.

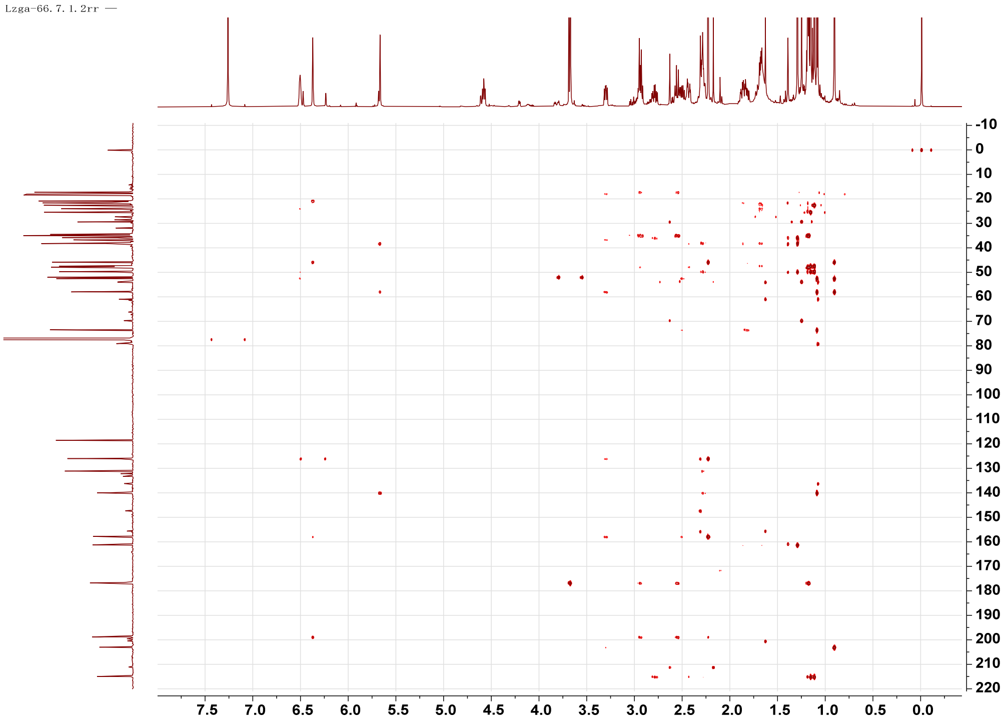

Figure S40. ^{1}H-^{1}H COSY spectrum of **6**.

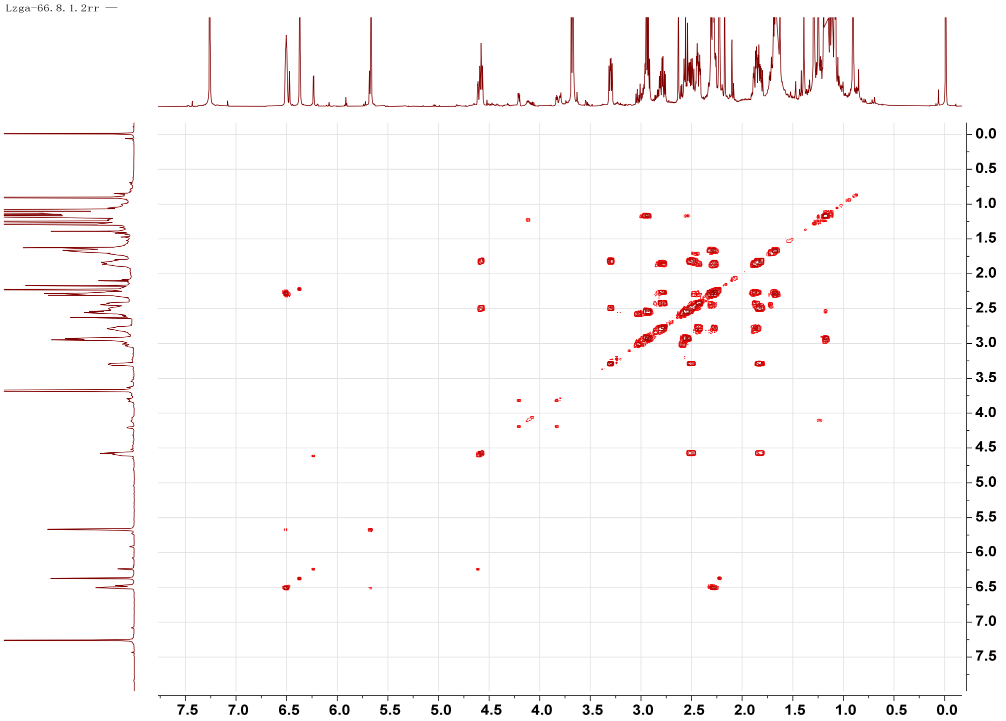

Figure S41. ROESY spectrum of **6**.

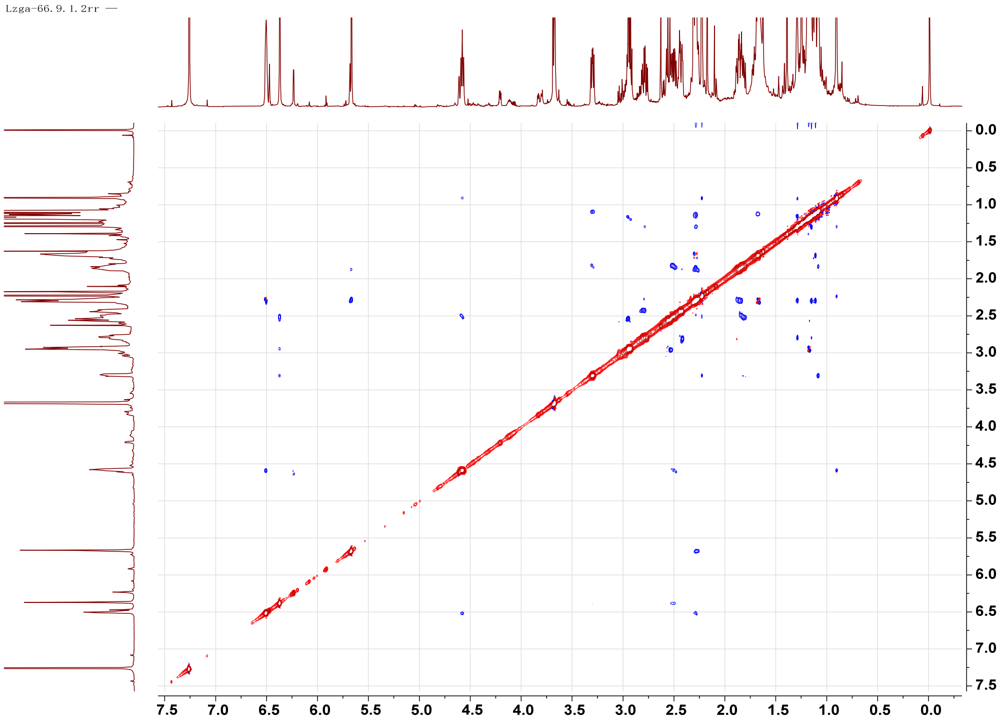

Figure S42. HRESIMS of **6**.

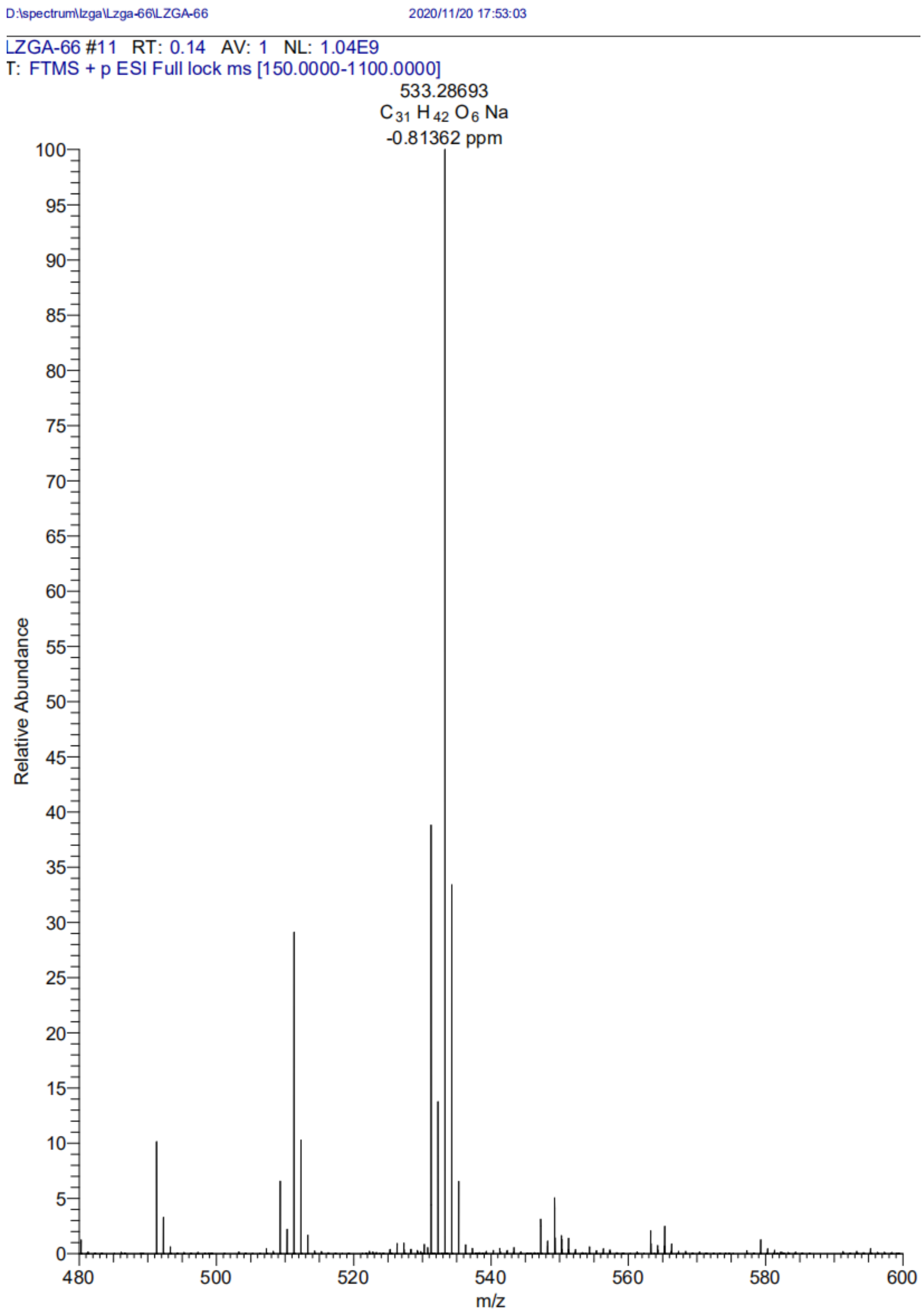

Figure S43. ^{1}H NMR spectrum of **7** (800 MHz, $CDCl_3$).

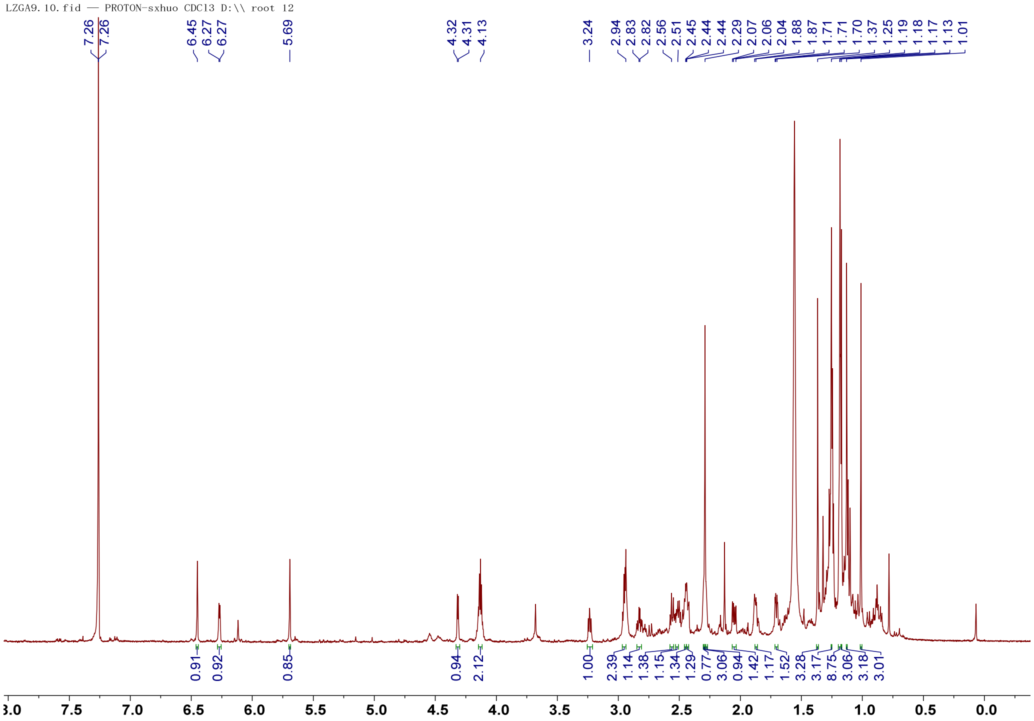

Figure S44. ^{13}C NMR spectra of **7** (200 MHz, $CDCl_3$).

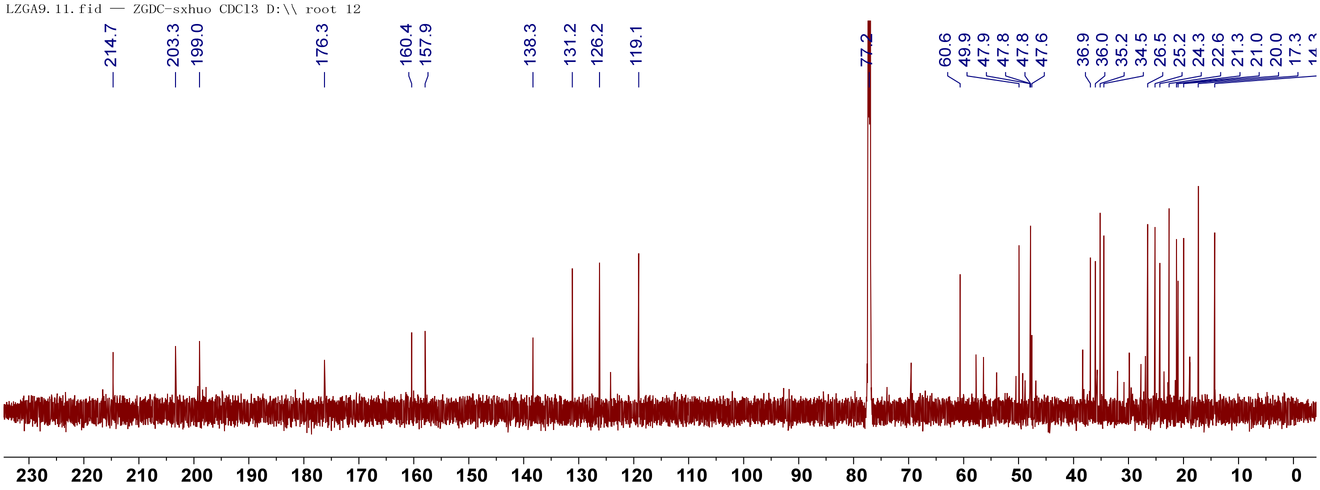

Figure S45. HSQC spectrum of **7**.

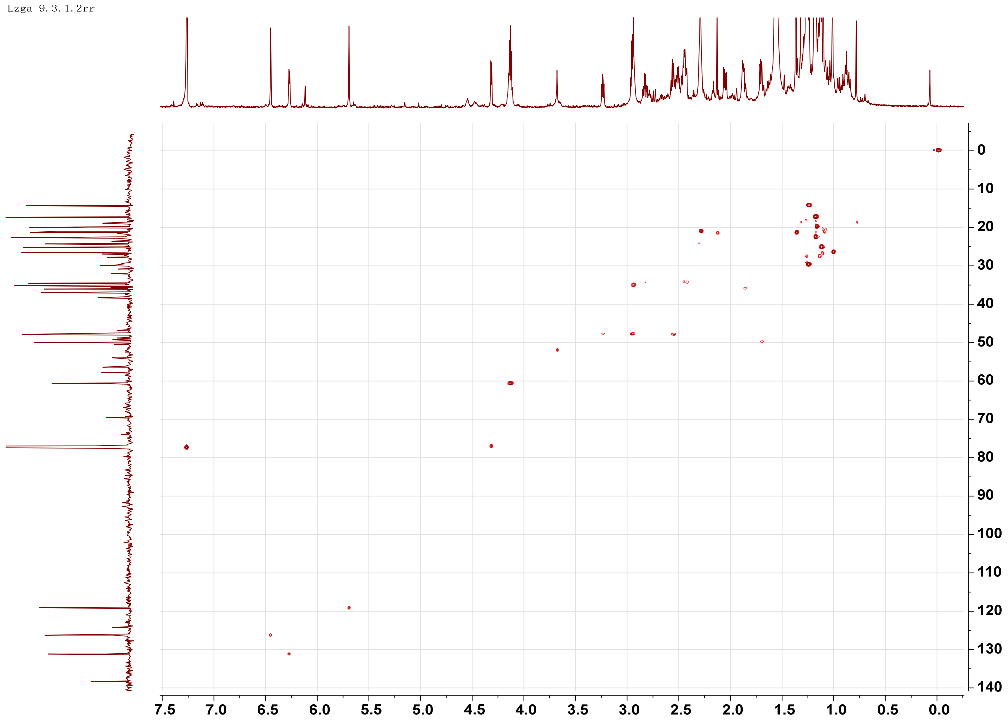

Figure S46. HMBC spectrum of **7**.

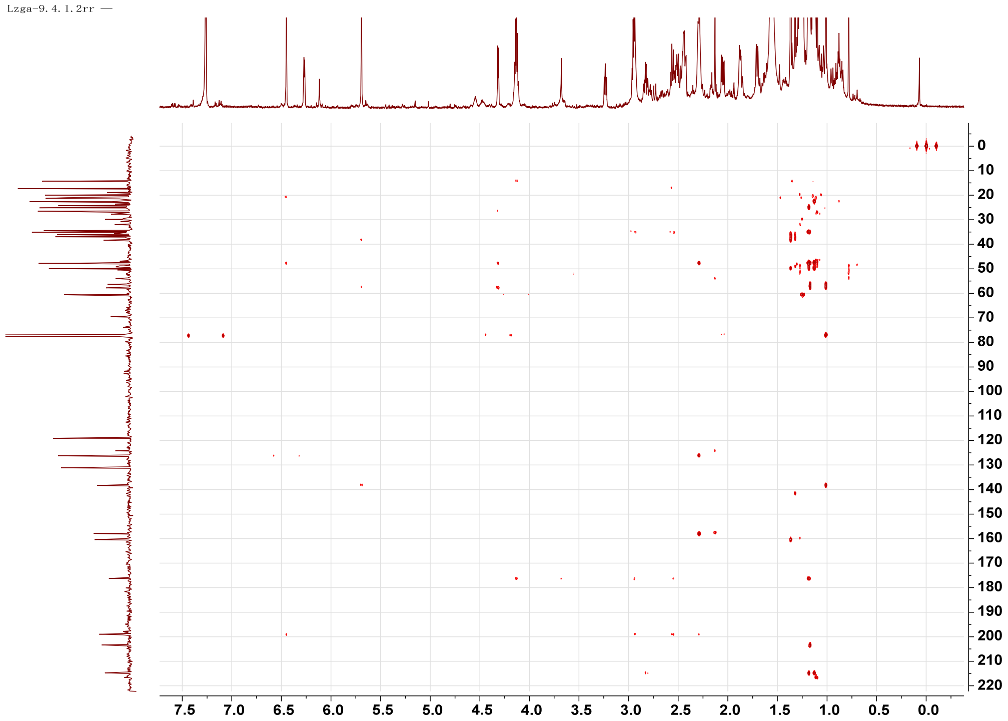

Figure S47. ^{1}H-^{1}H COSY spectrum of **7**.

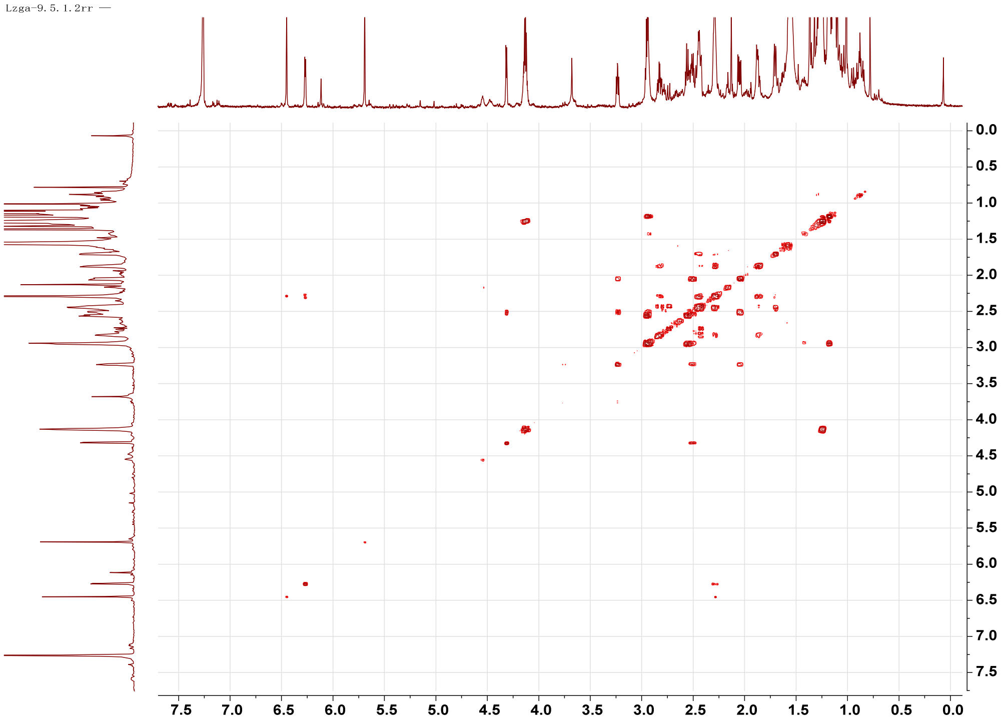

Figure S48. ROESY spectrum of **7**.

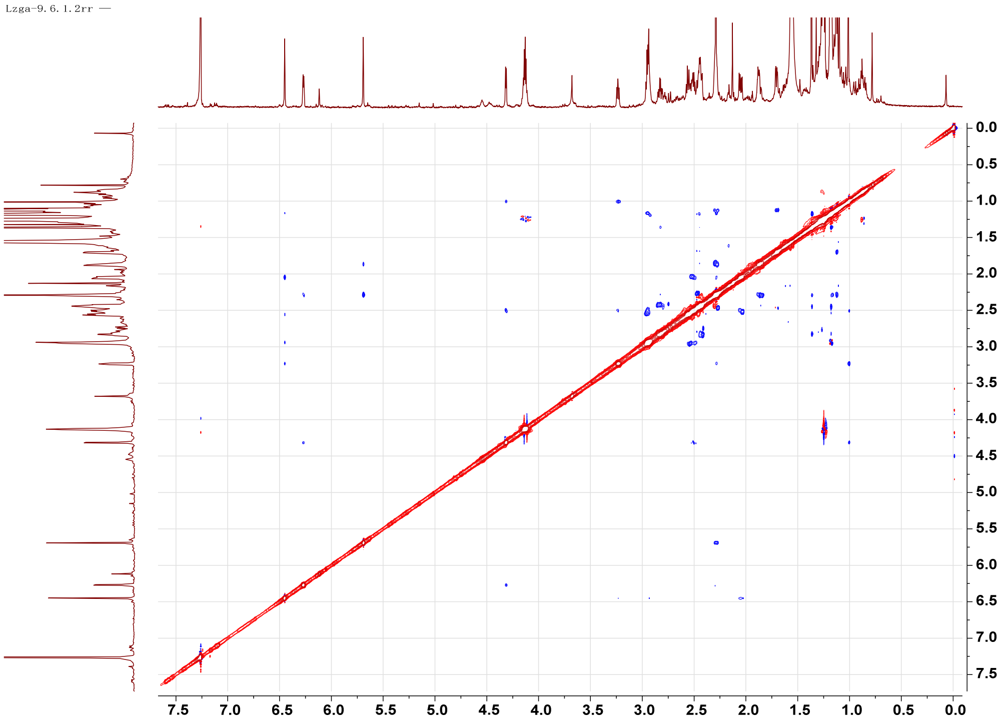

Figure S49. HRESIMS of **7**.

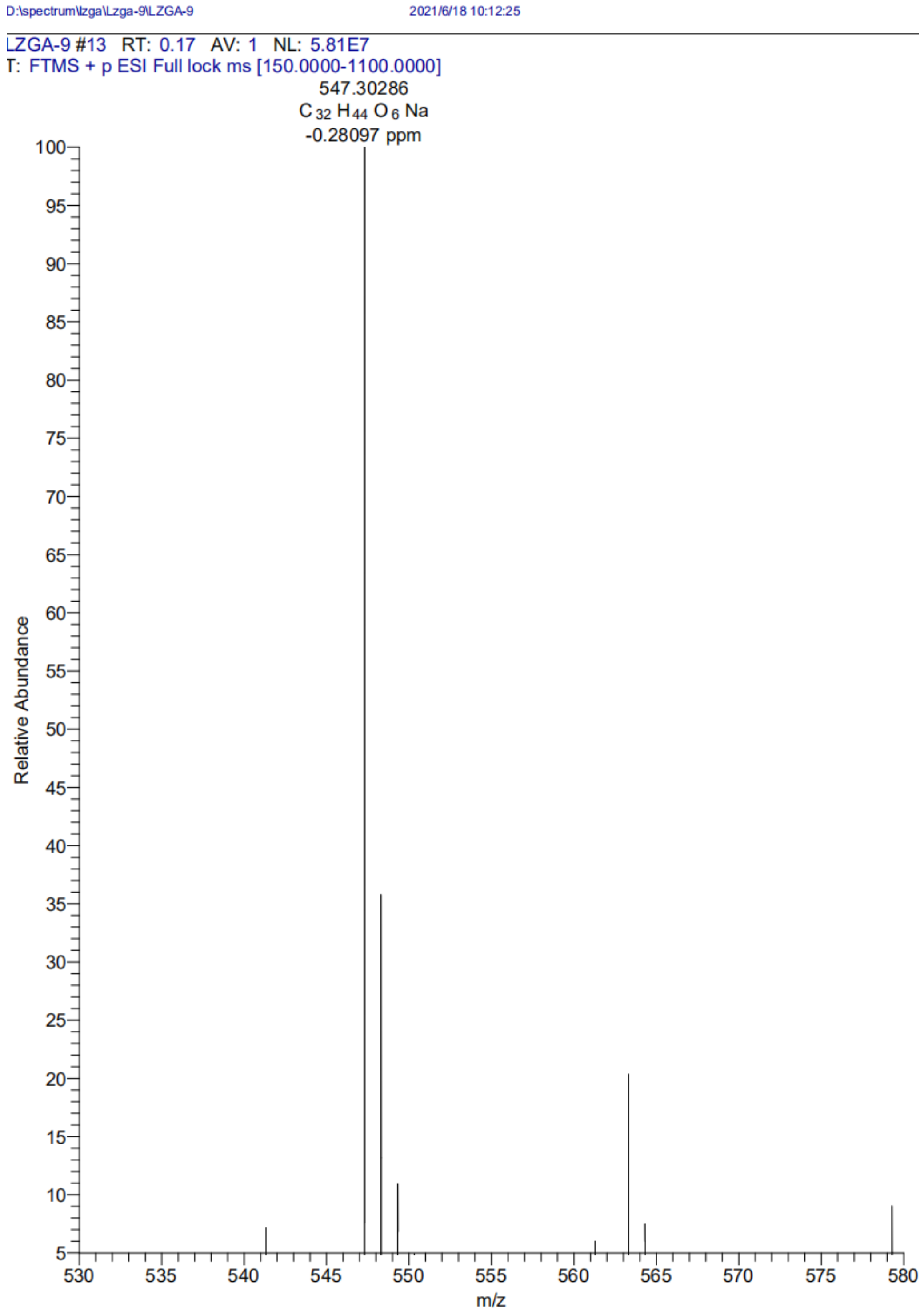

Figure S50. ^{1}H NMR spectrum of **8** (600 MHz, $CDCl_3$).

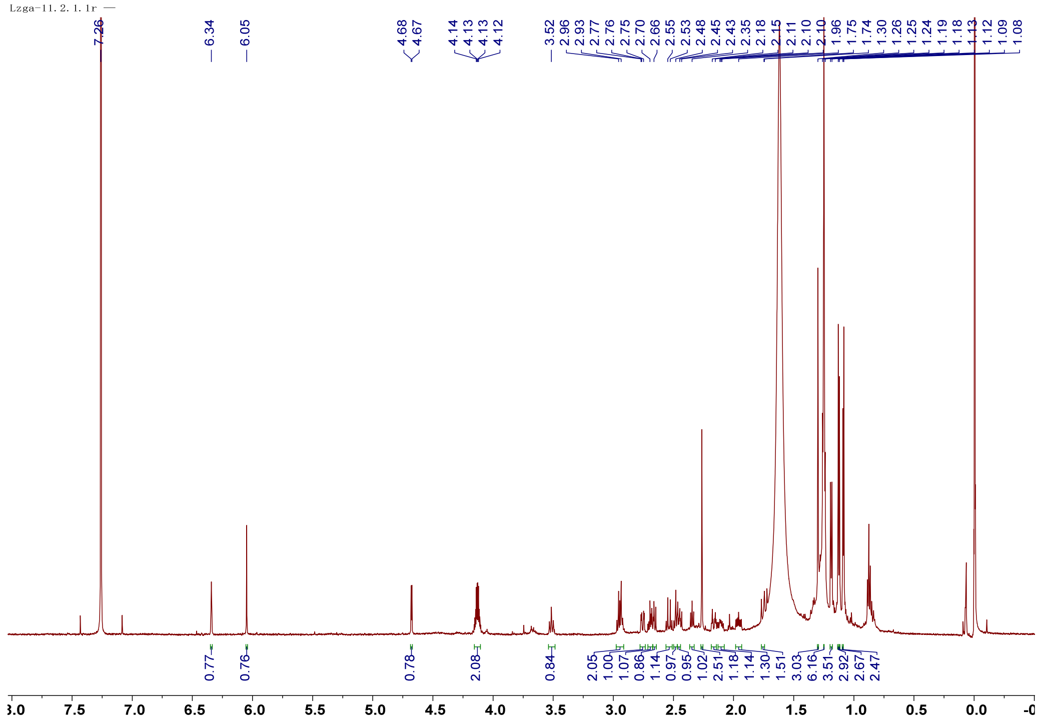

Figure S51. ^{13}C NMR spectra of **8** (150 MHz, $CDCl_3$).

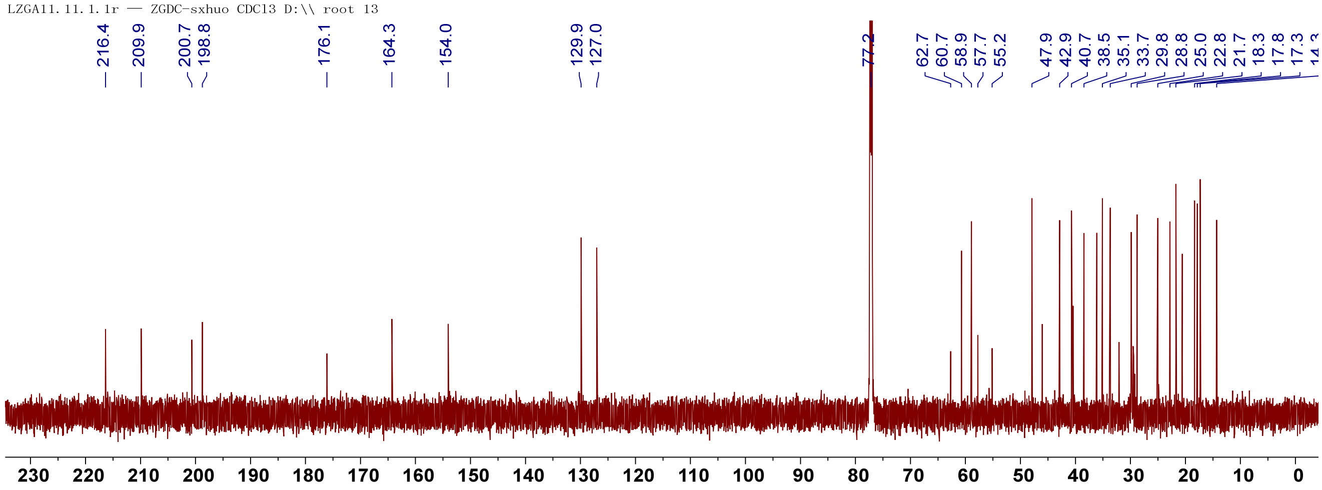

Figure S52. HSQC spectrum of **8**.

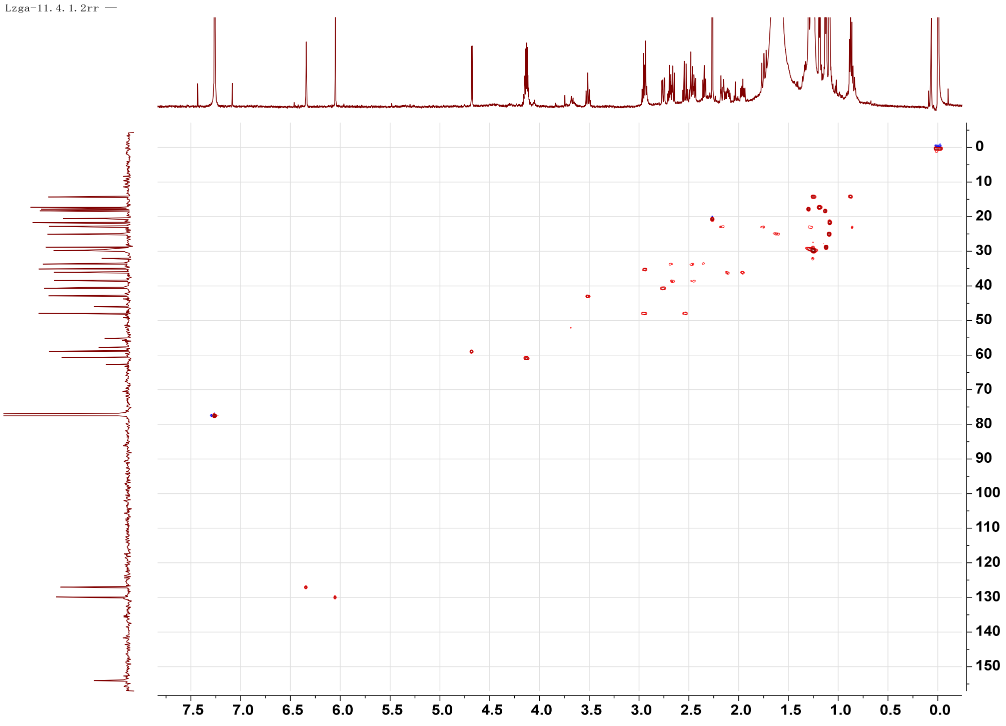

Figure S53. HMBC spectrum of **8**.

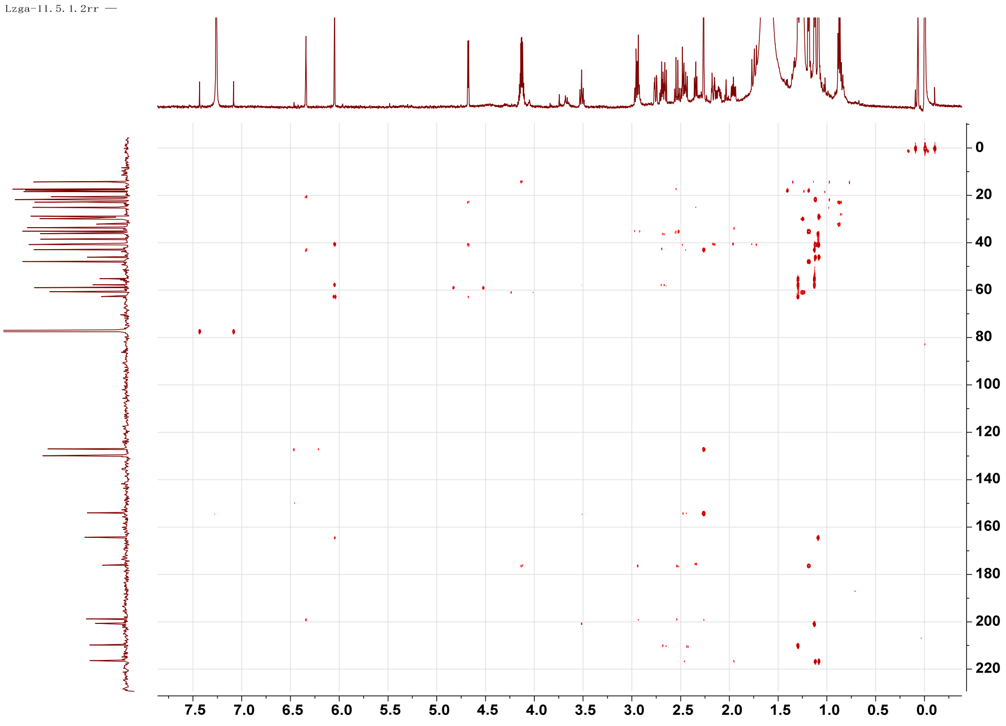

Figure S54. ^{1}H-^{1}H COSY spectrum of **8**.

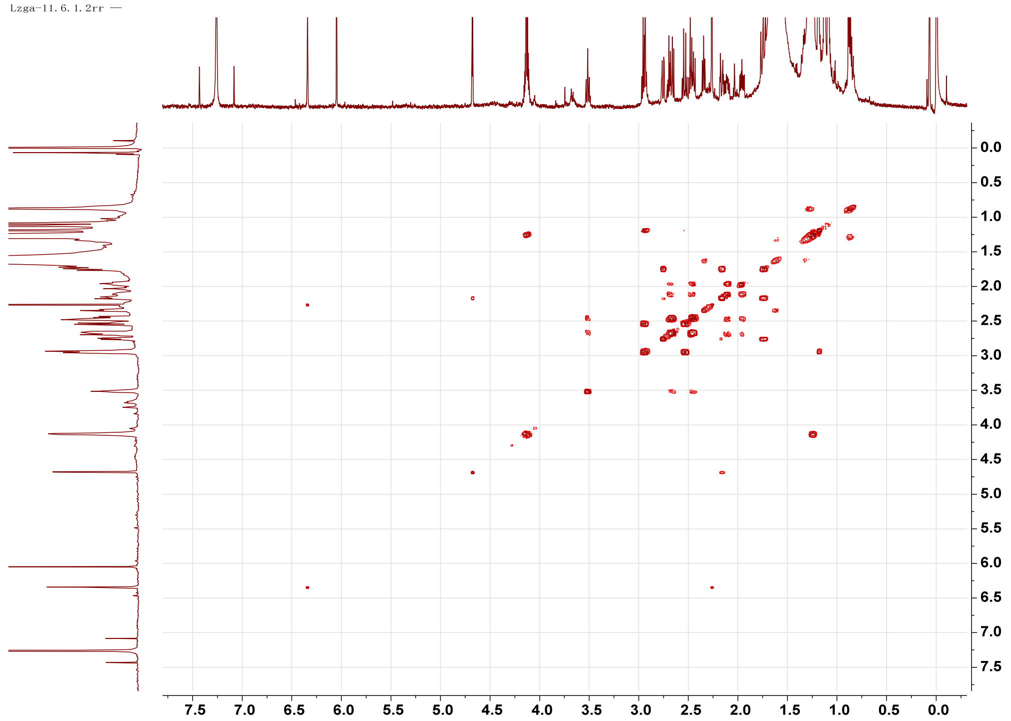

Figure S55. ROESY spectrum of **8**.

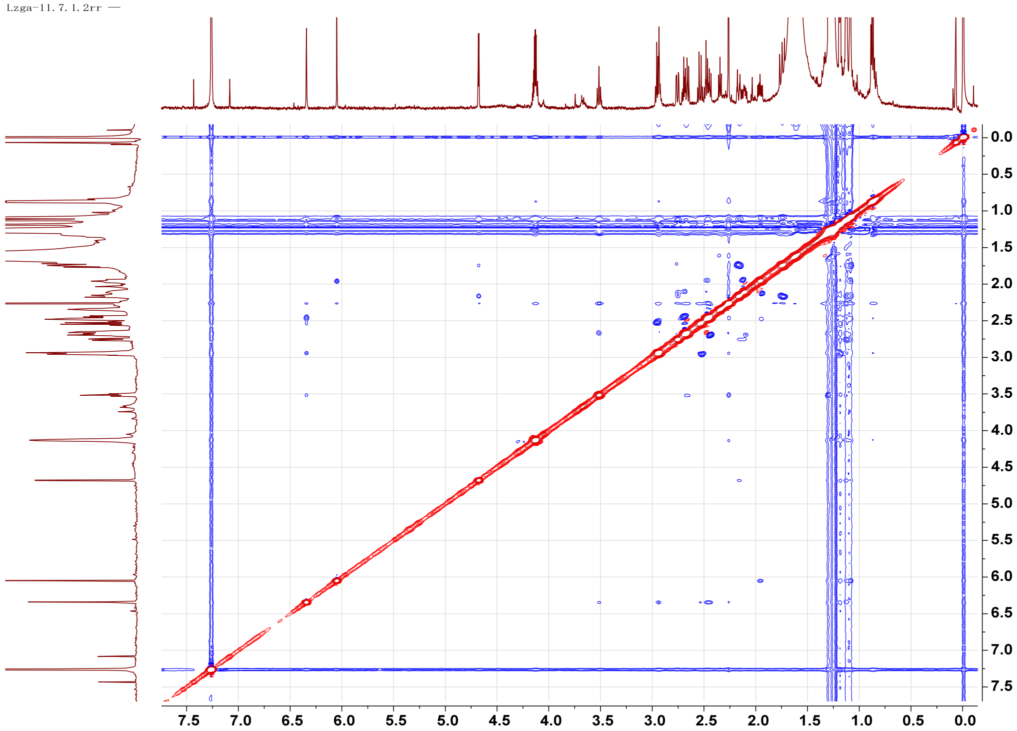

Figure S56. HRESIMS of **8**.

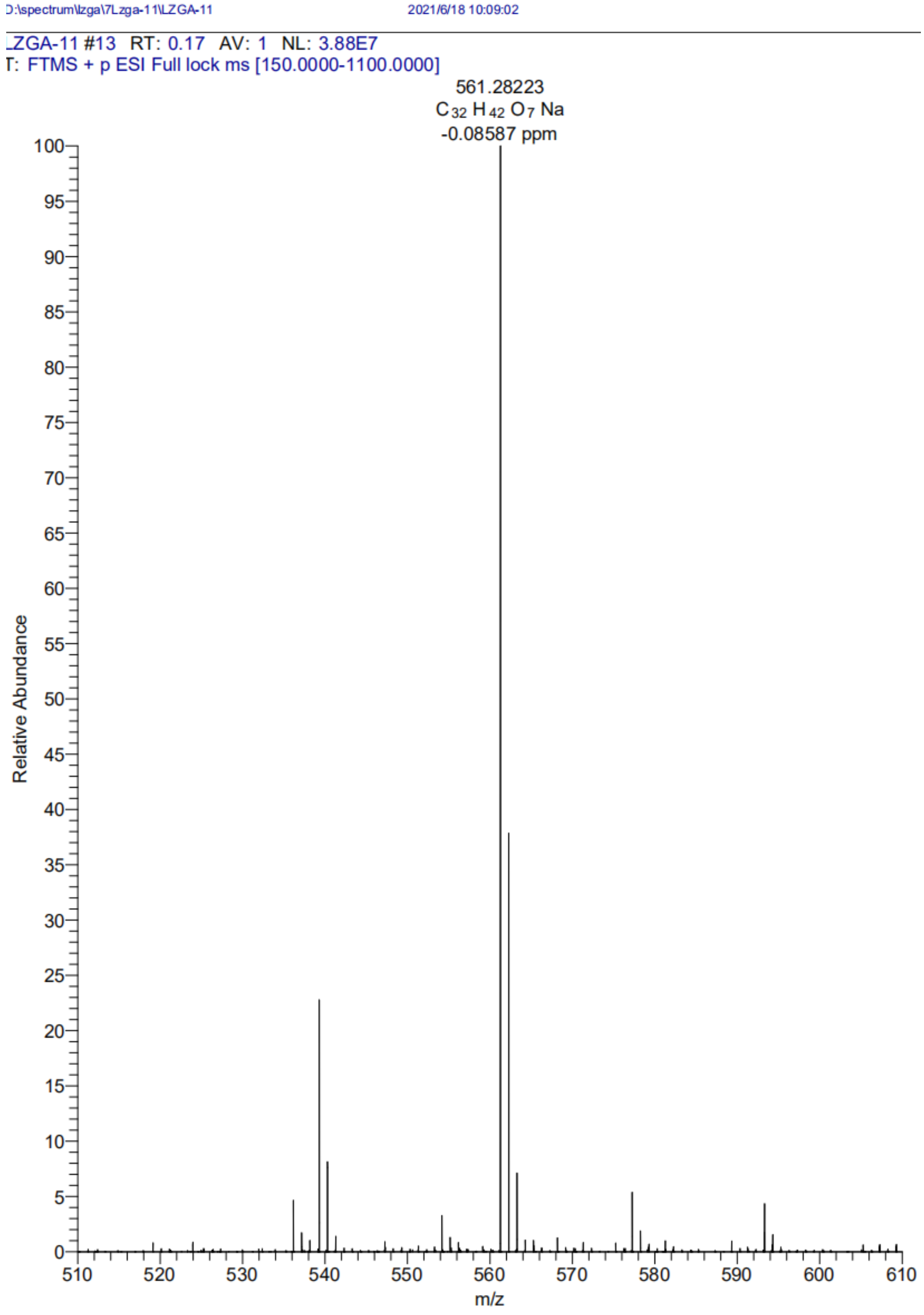

Figure S57. ^{1}H NMR spectrum of **9** (600 MHz, $CDCl_3$).

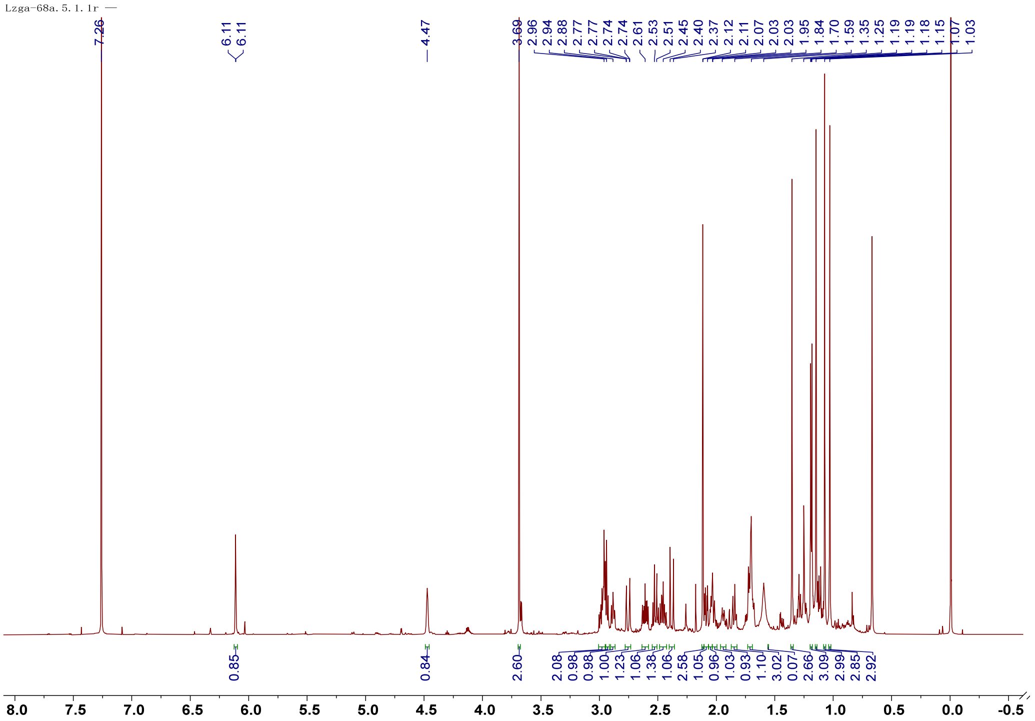

Figure S58. ^{13}C and DEPT NMR spectra of **9** (150 MHz, $CDCl_3$).

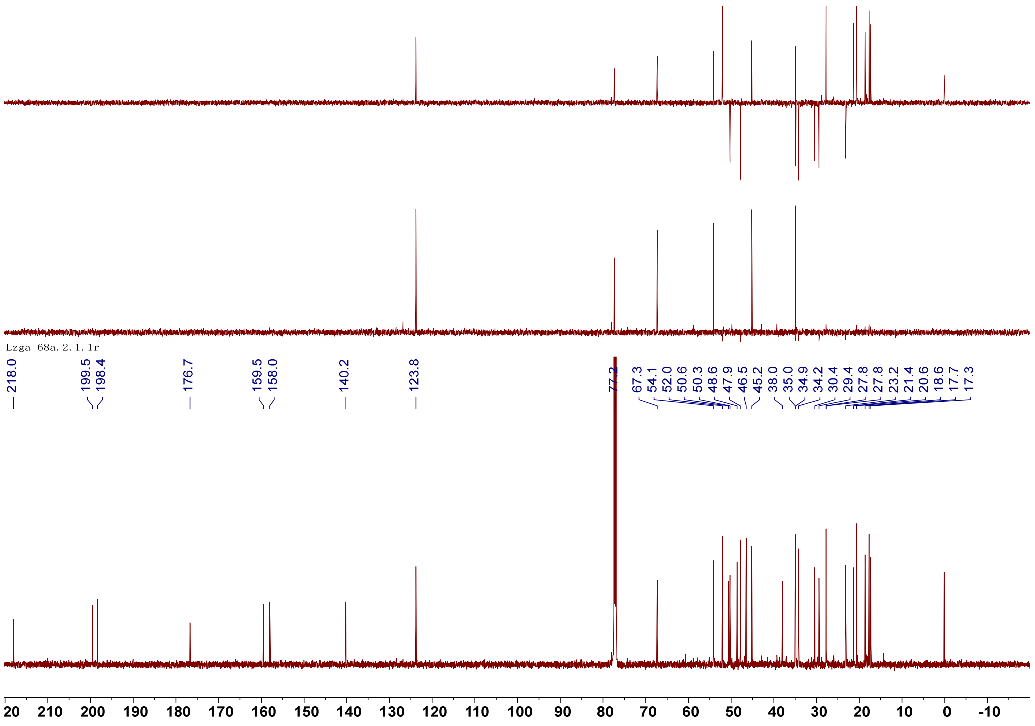

Figure S59. HSQC spectrum of **9**.

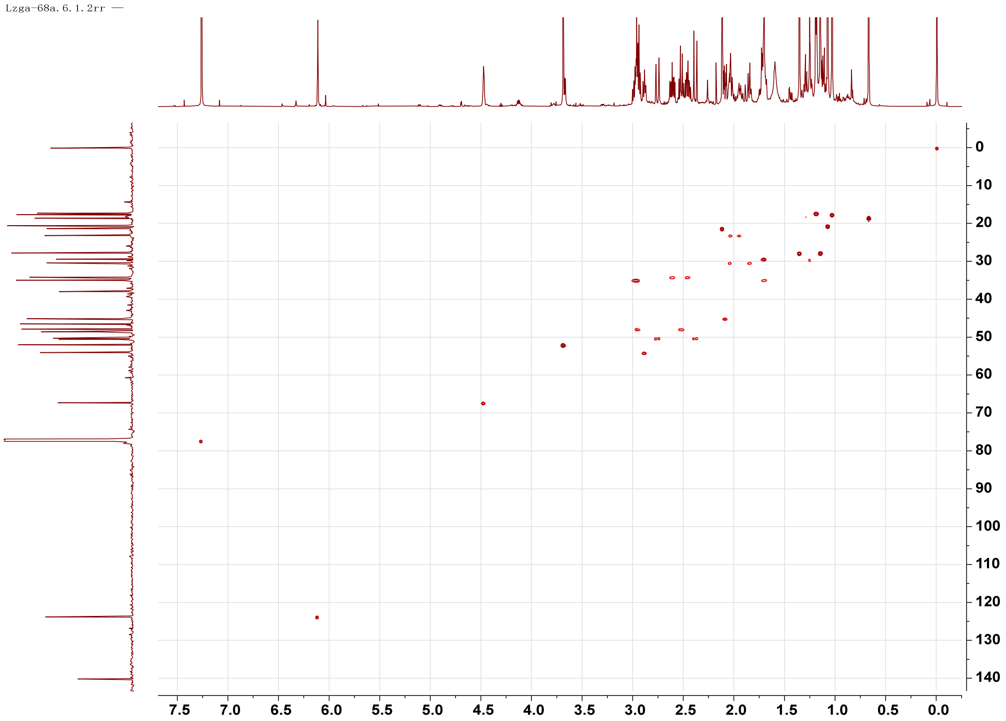

Figure S60. HMBC spectrum of **9**.

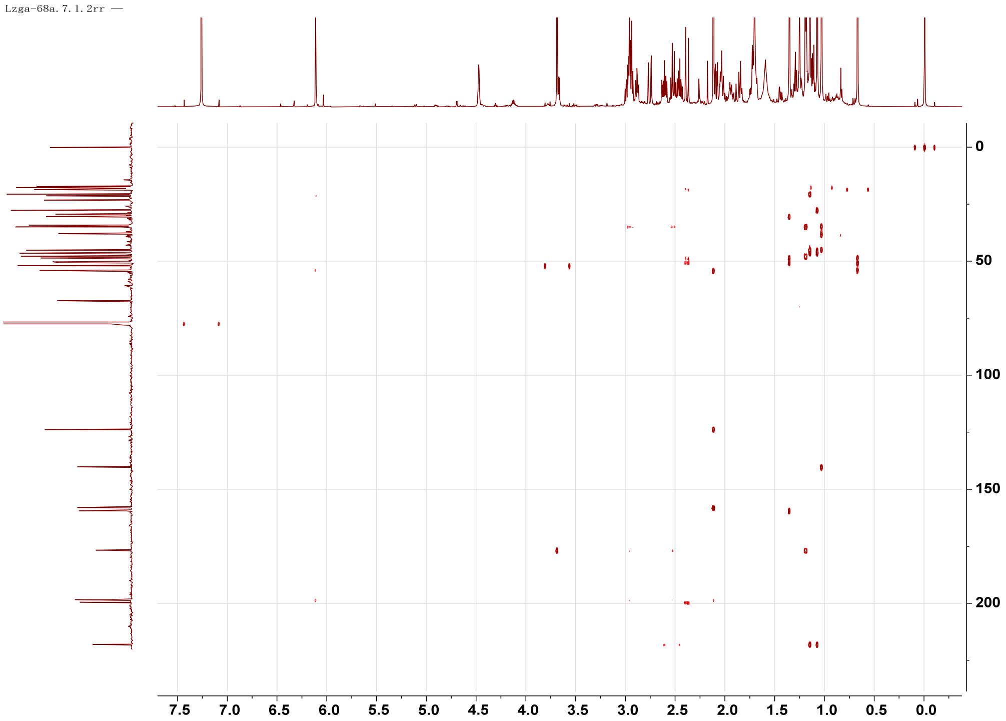

Figure S61. ^{1}H-^{1}H COSY spectrum of **9**.

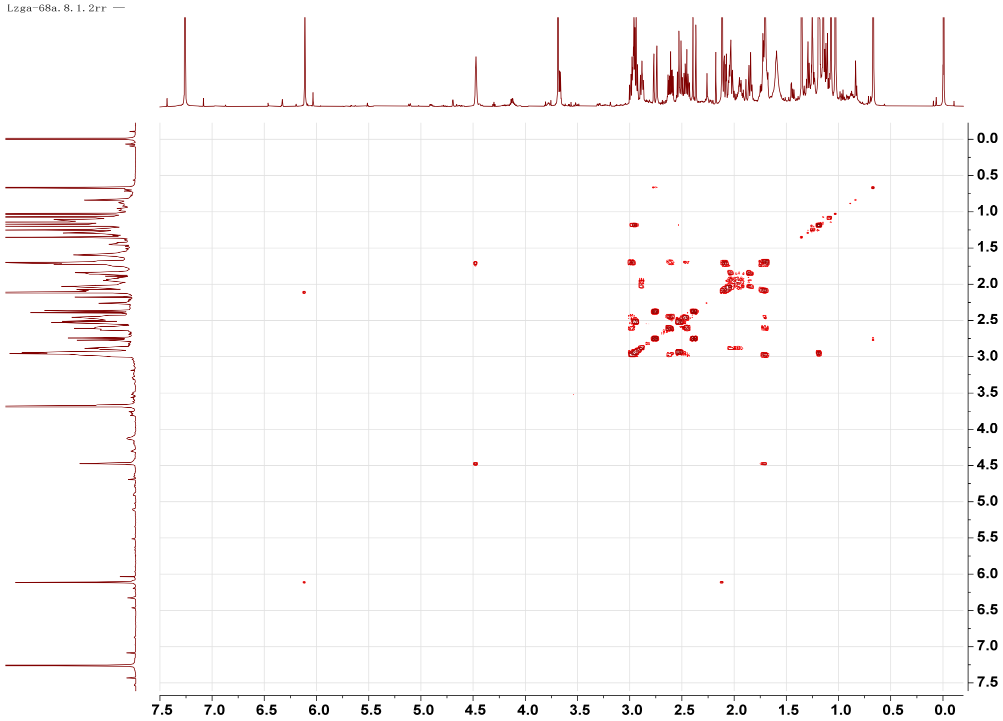

Figure S62. ROESY spectrum of **9**.

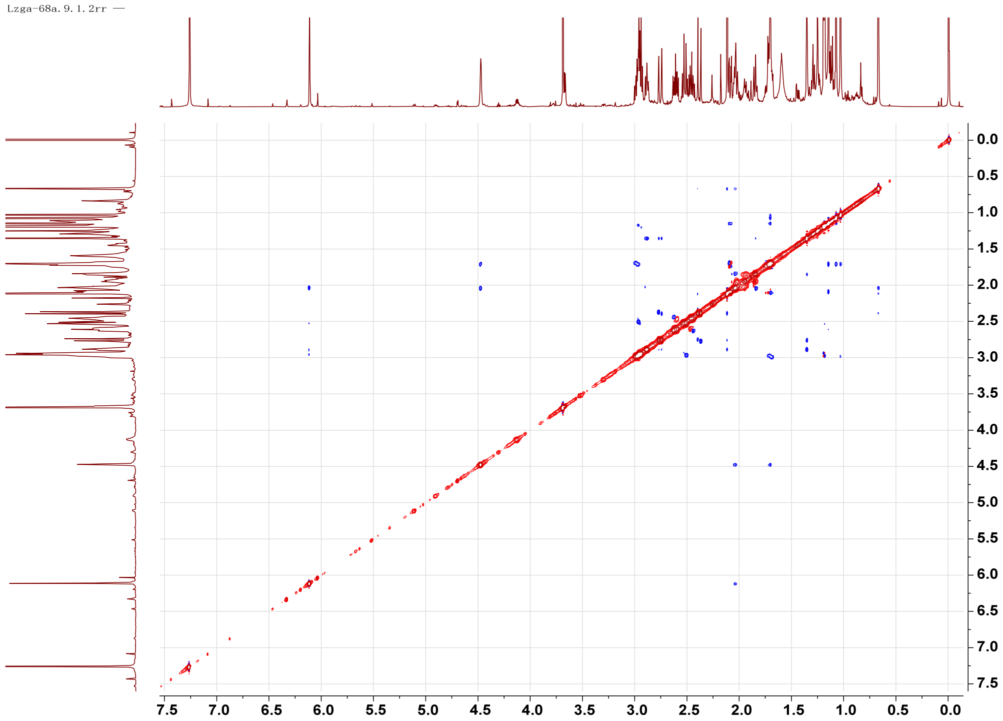

Figure S63. HRESIMS of **9**.

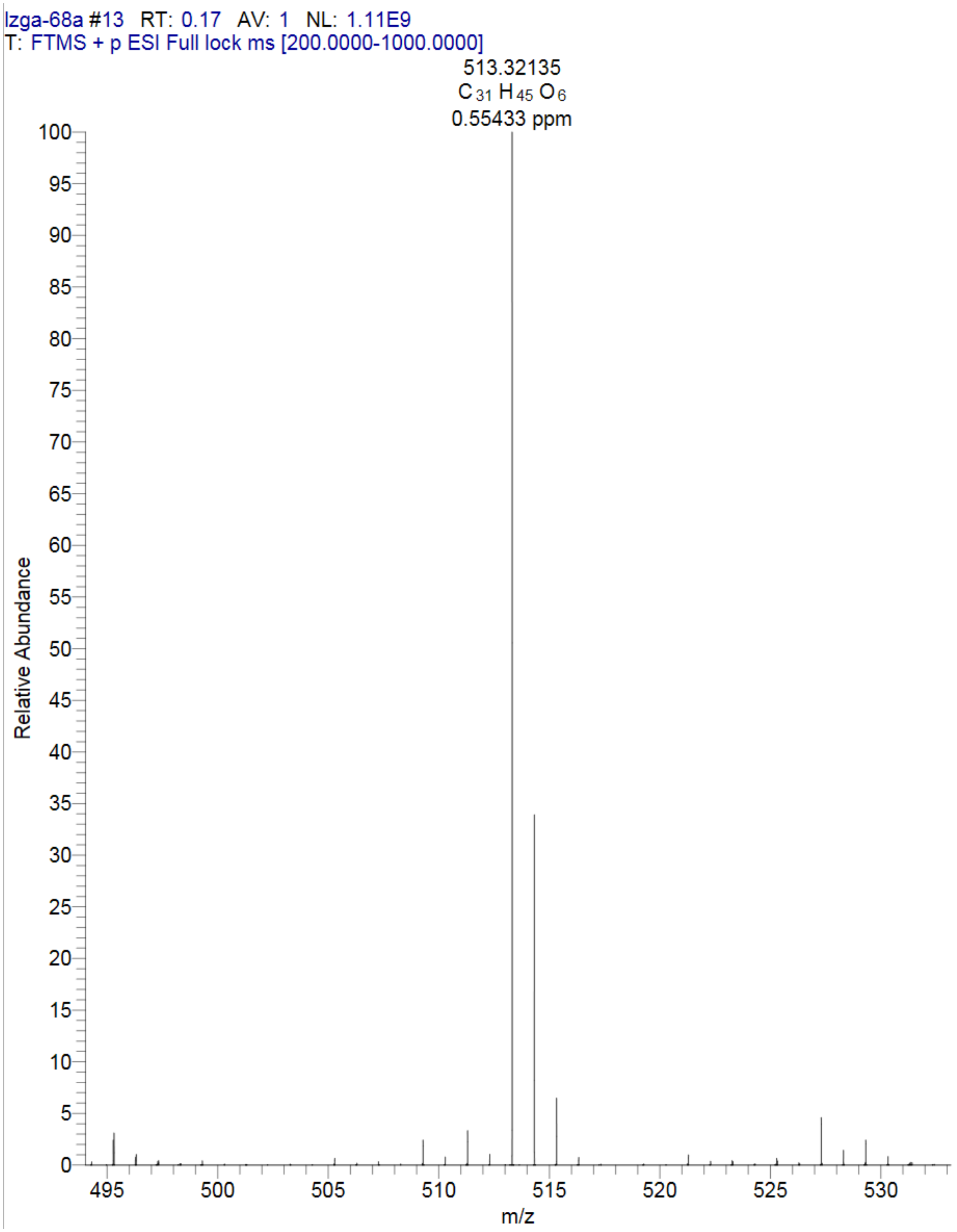

Figure S64. ^{1}H NMR spectrum of **10** (600 MHz, $CDCl_3$).

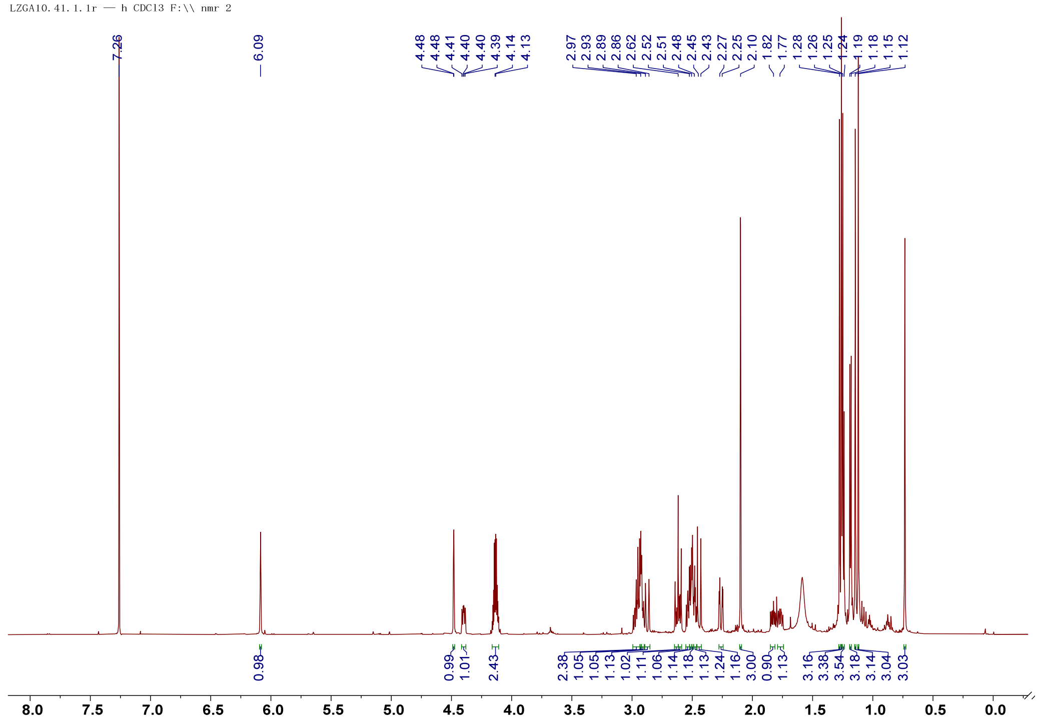

Figure S65. ^{13}C and DEPT NMR spectra of **10** (150 MHz, $CDCl_3$).

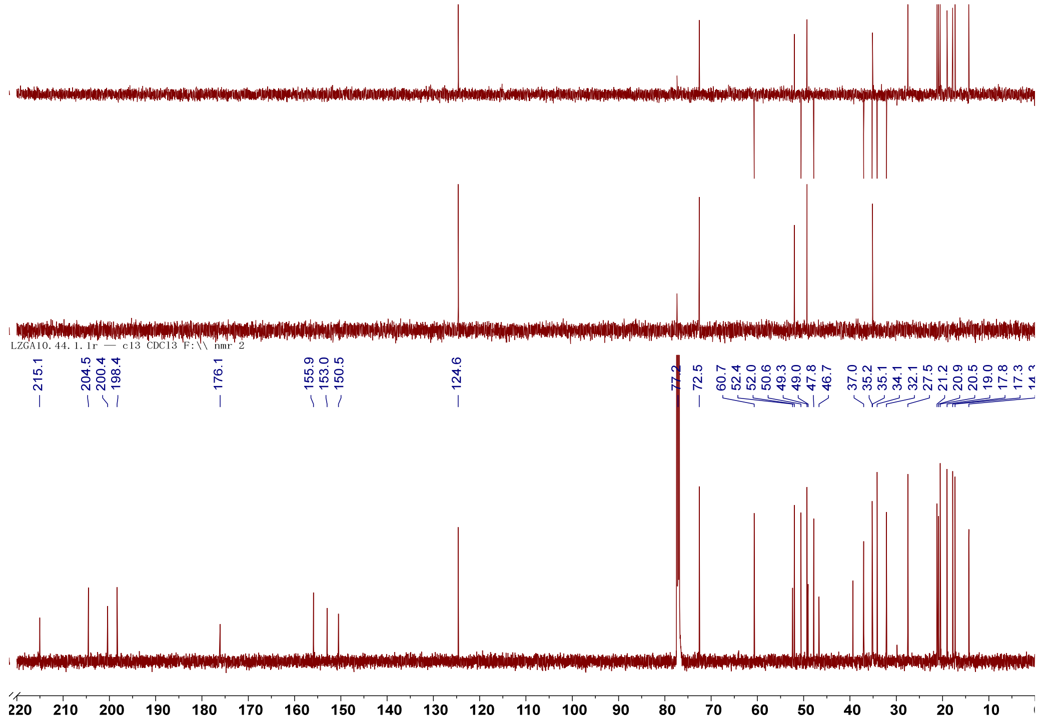

Figure S66. HSQC spectrum of **10**.

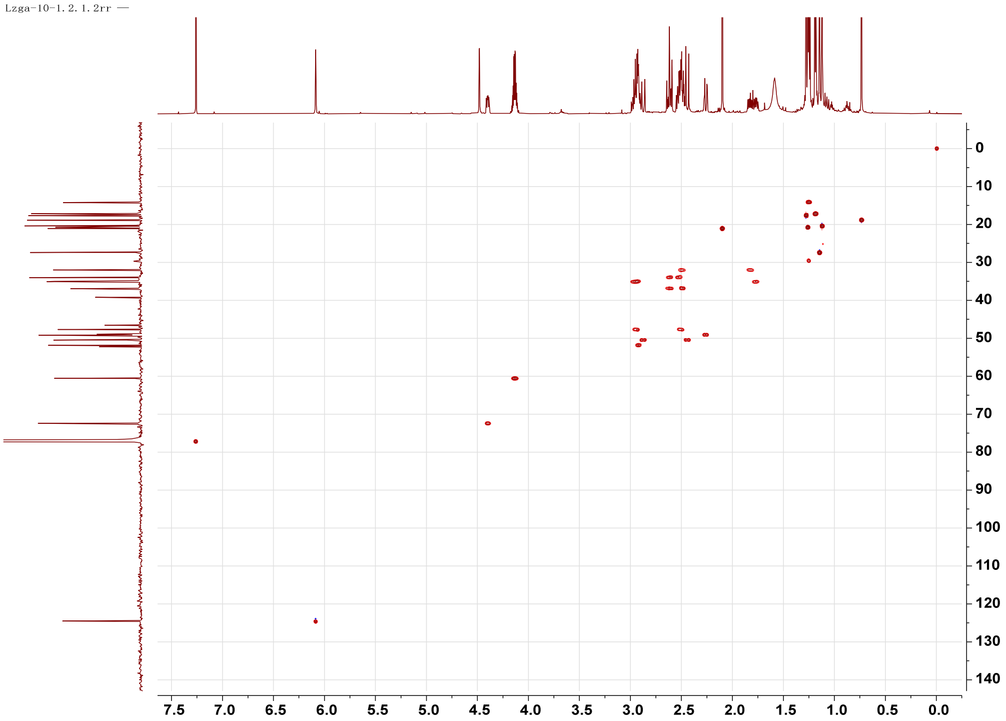

Figure S67. HMBC spectrum of **10**.

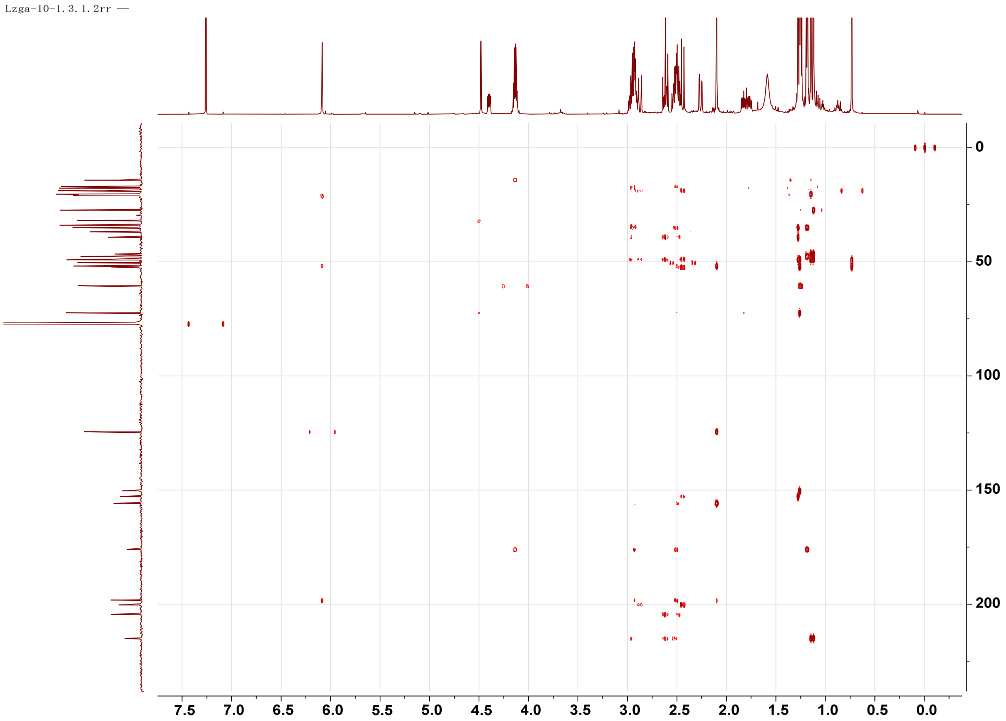

Figure S68. ^{1}H-^{1}H COSY spectrum of **10**.

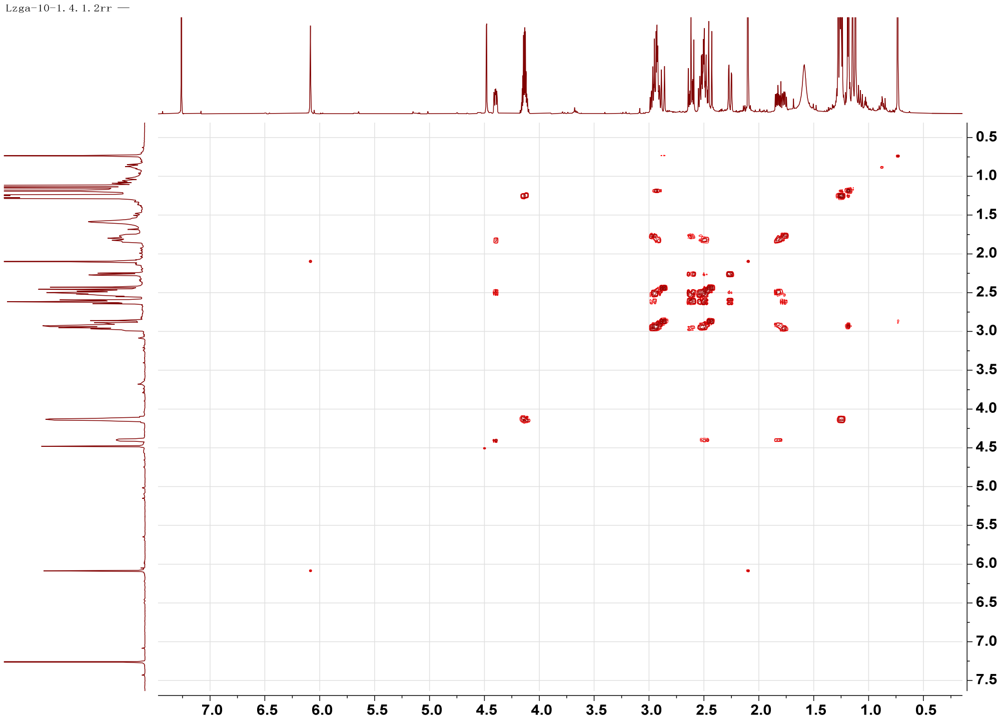

Figure S69. ROESY spectrum of **10**.

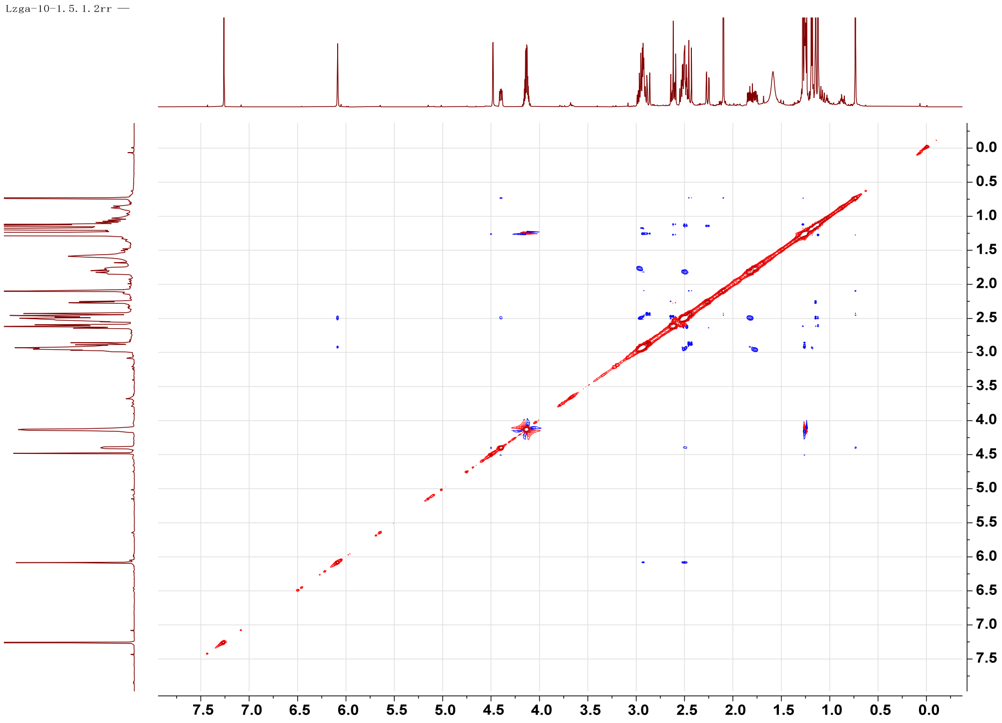

Figure S70. HRESIMS of **10**.

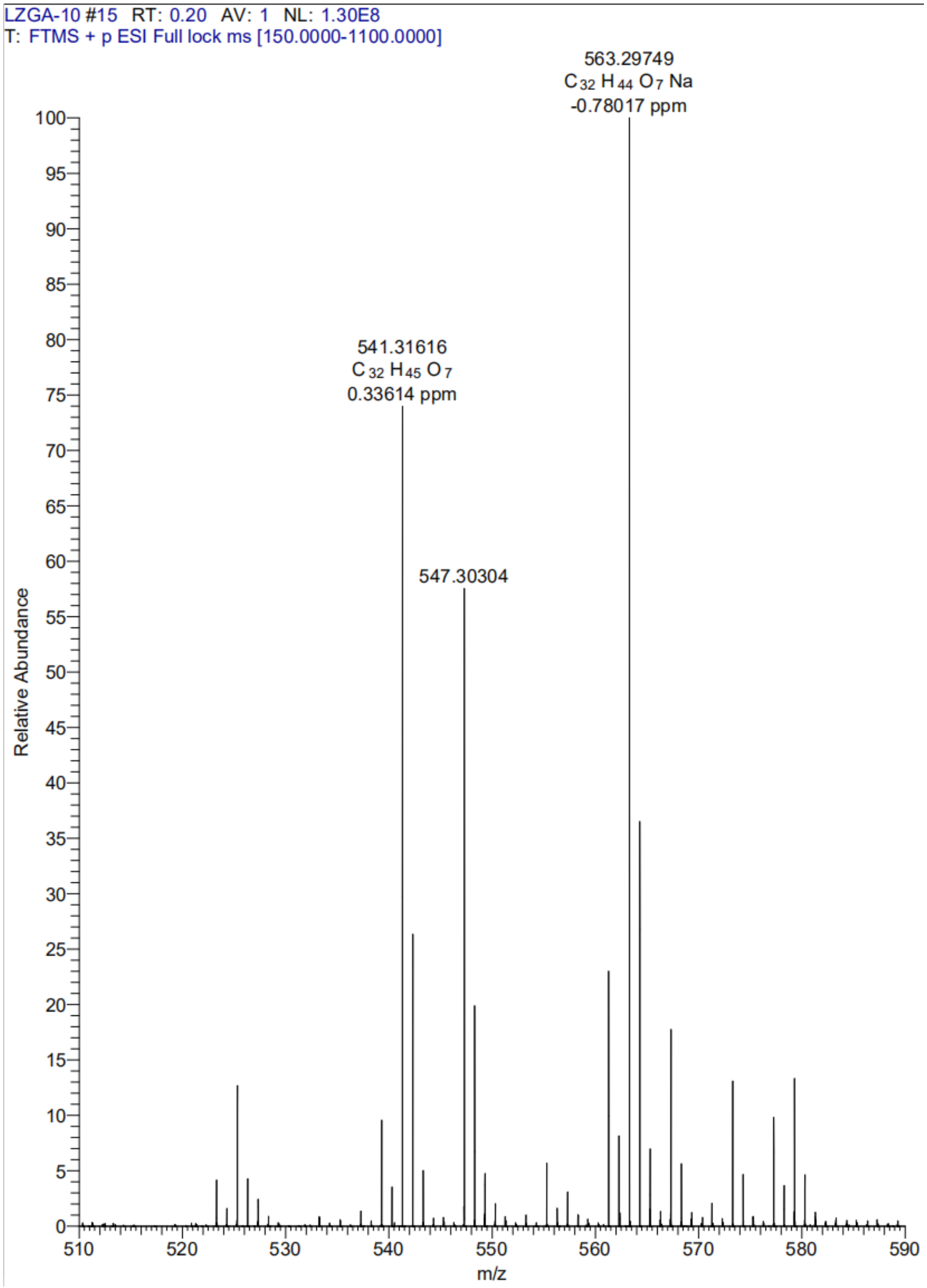

Figure S71. ^{1}H NMR spectrum of **11** (600 MHz, $CDCl_3$).

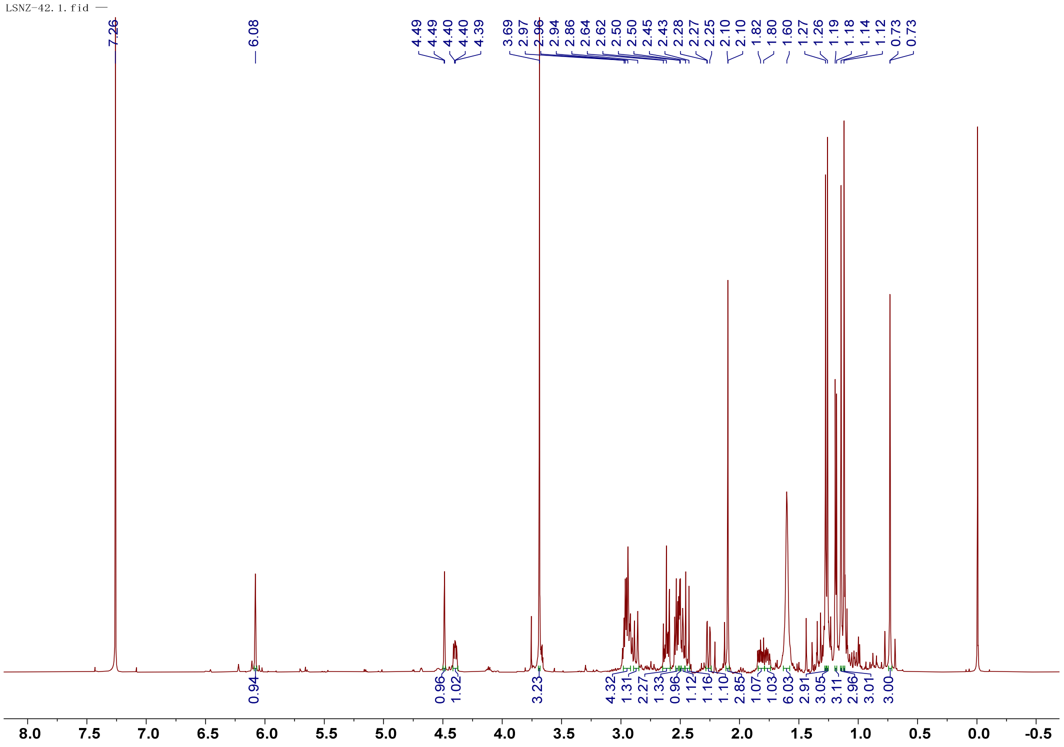

Figure S72. ^{13}C and DEPT NMR spectra of **11** (150 MHz, $CDCl_3$).

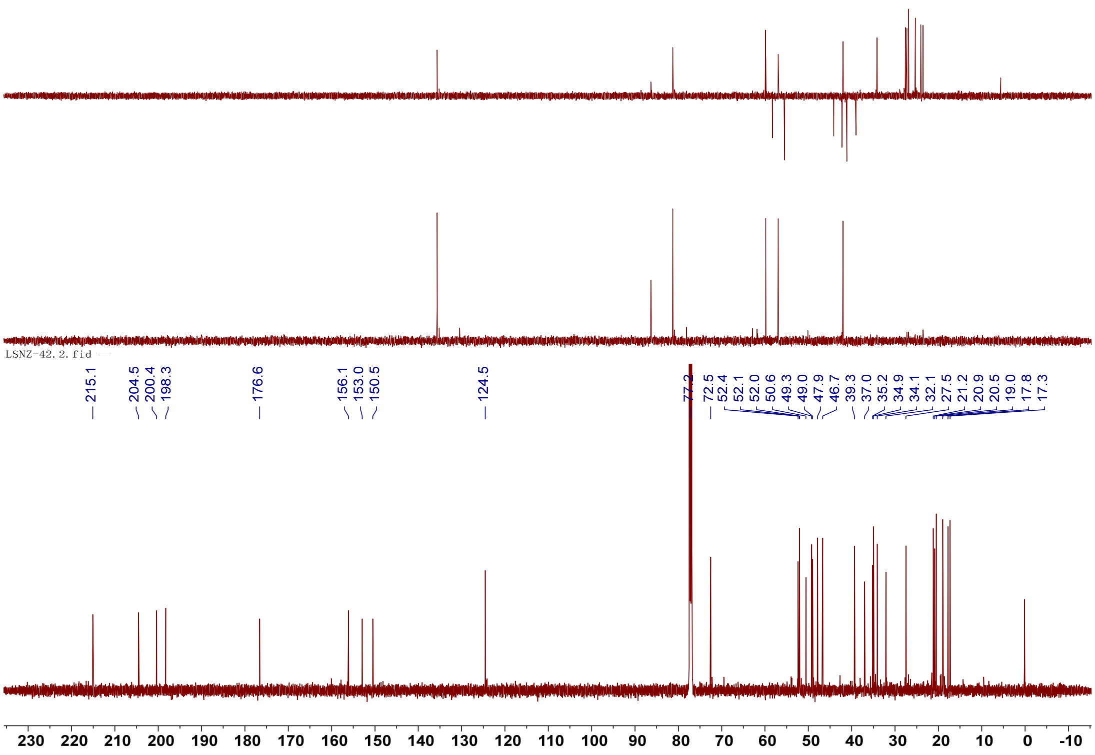

Figure S73. HSQC spectrum of **11**.

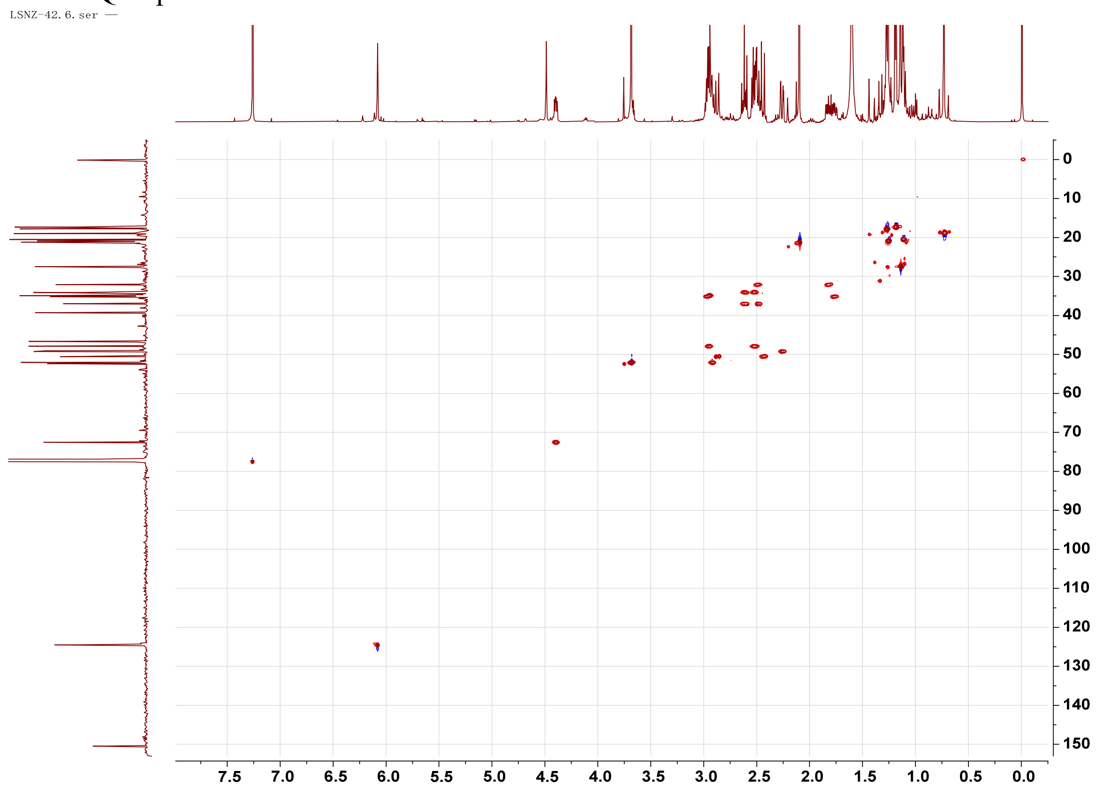

Figure S74. HMBC spectrum of **11**.

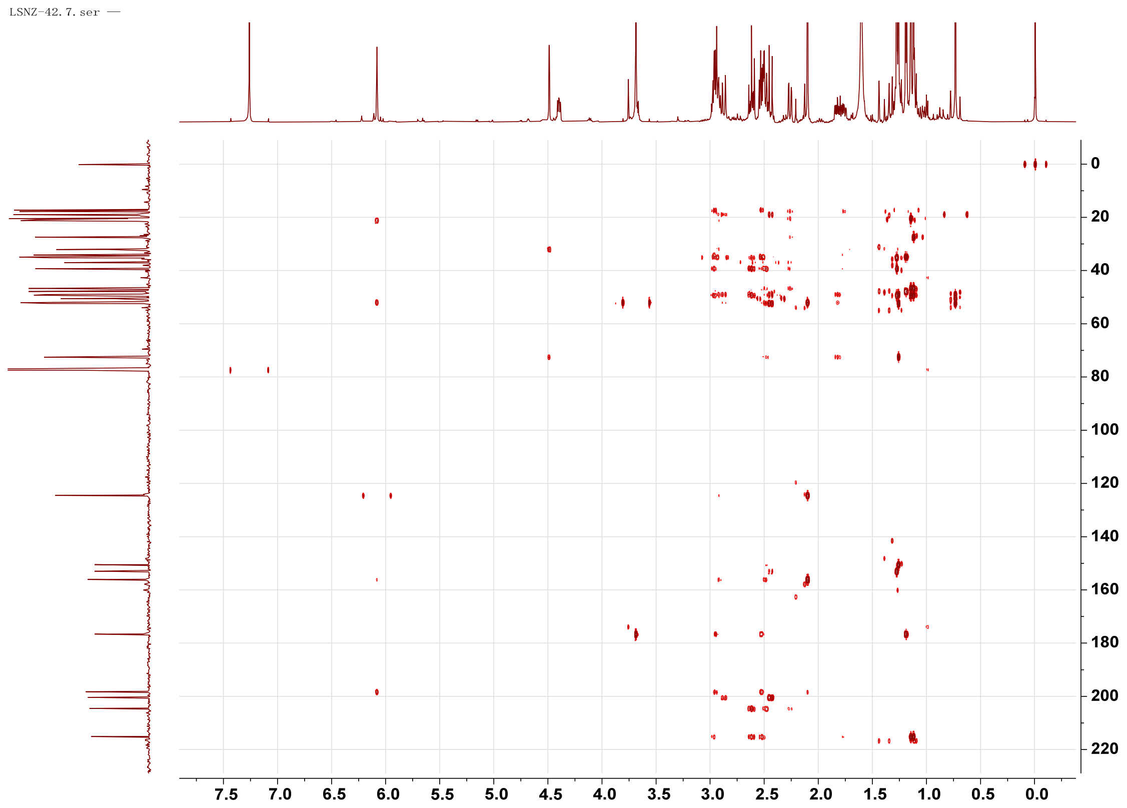

Figure S75. ^{1}H-^{1}H COSY spectrum of **11**.

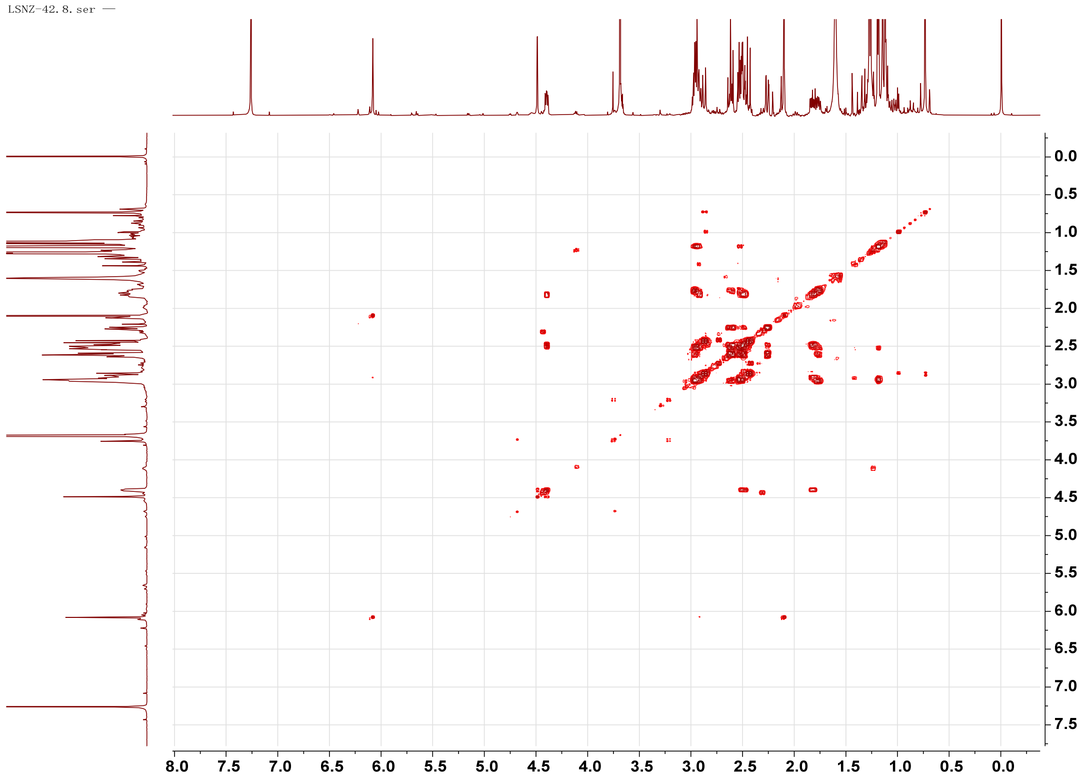

Figure S76. ROESY spectrum of **11**.

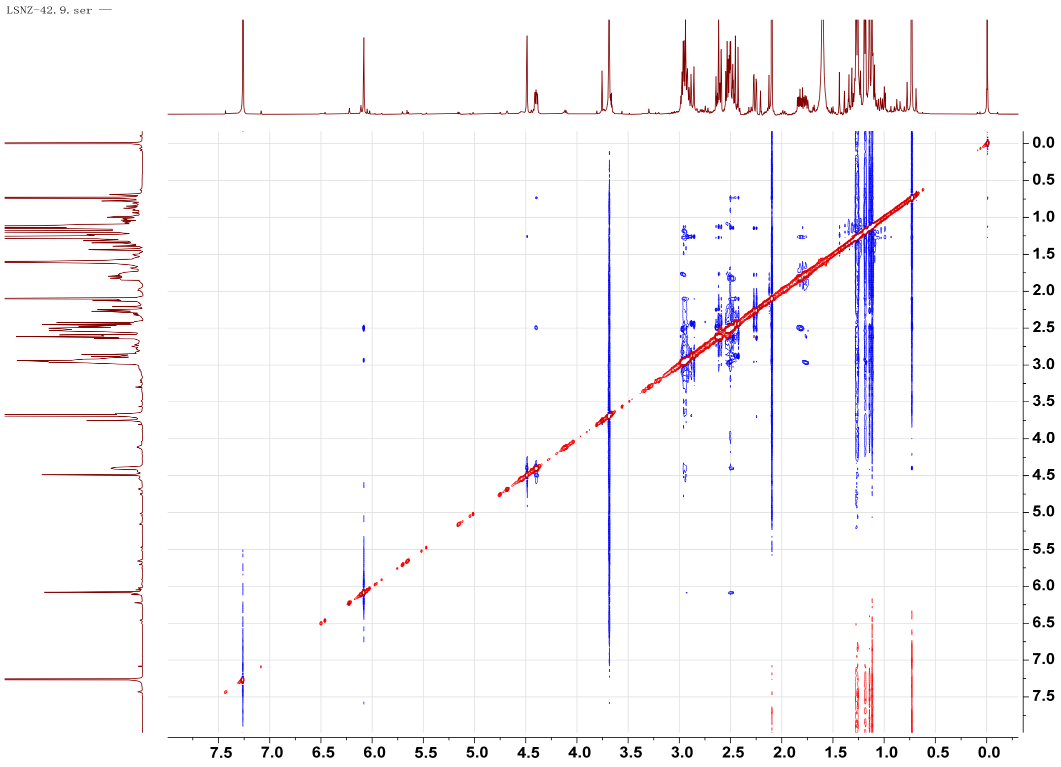

Figure S77. HRESIMS of **11**.

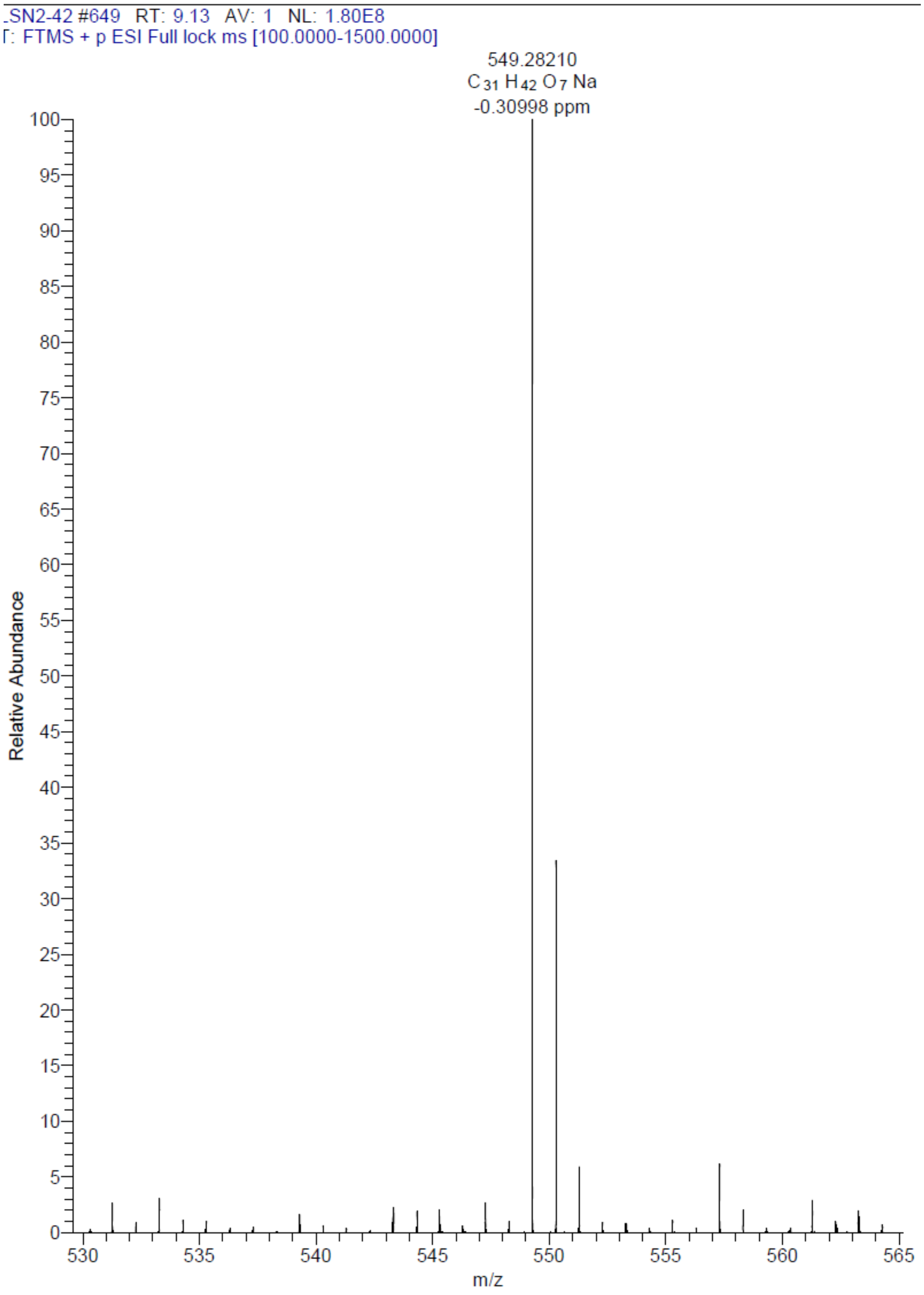

Figure S78. ^{1}H NMR spectrum of **12** (600 MHz, $CDCl_3$).

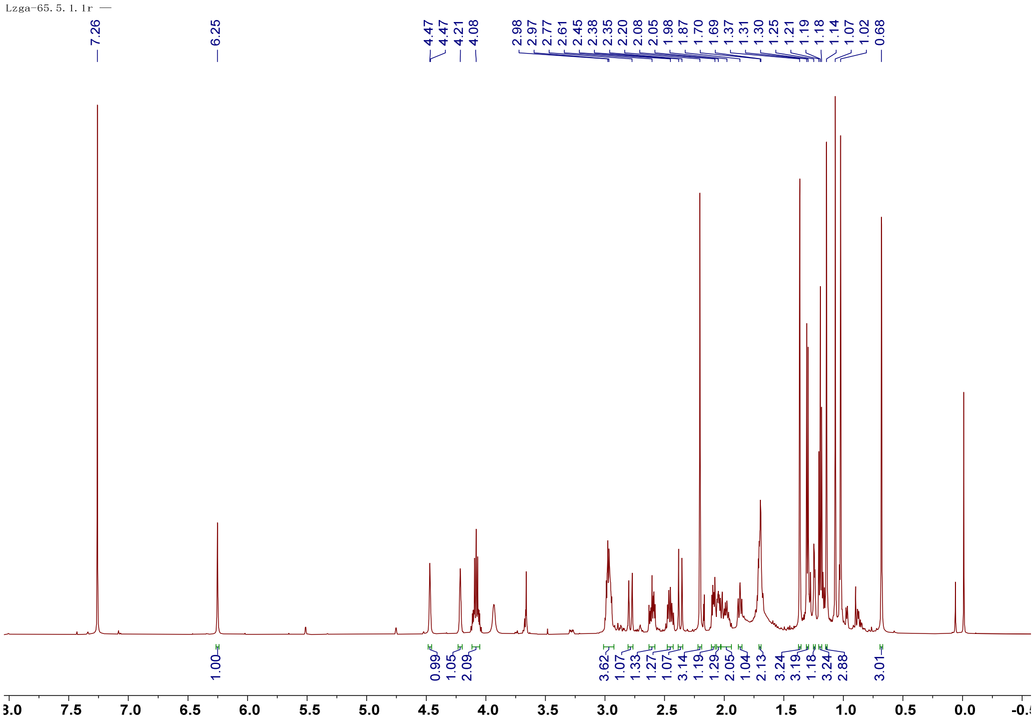

Figure S79. ^{13}C and DEPT NMR spectra of **12** (150 MHz, $CDCl_3$).

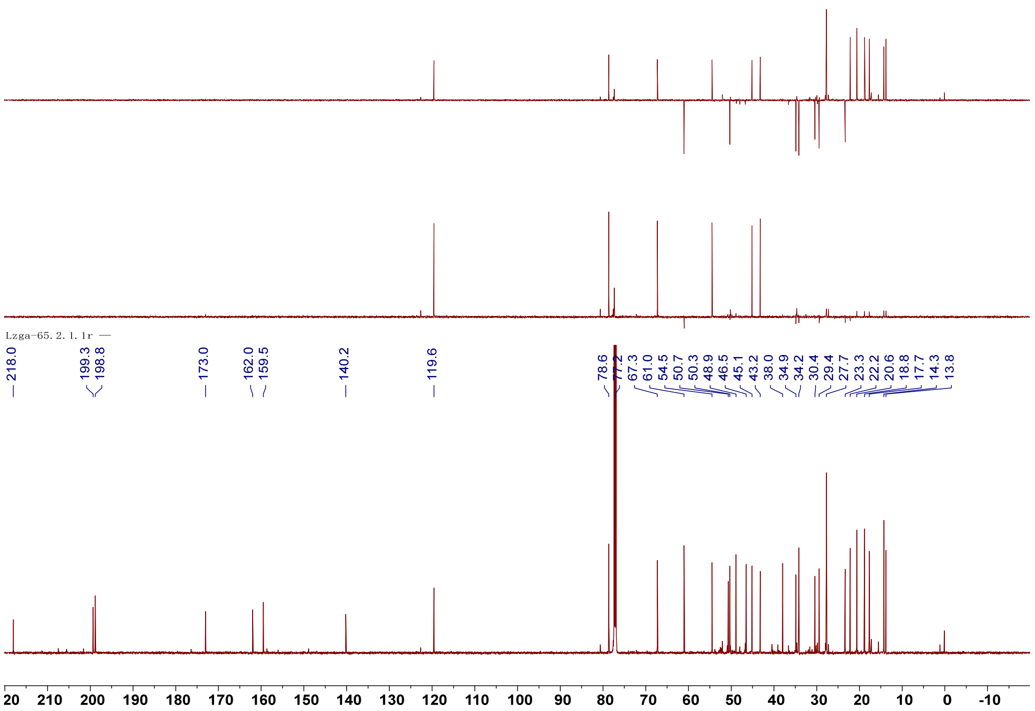

Figure S80. HSQC spectrum of **12**.

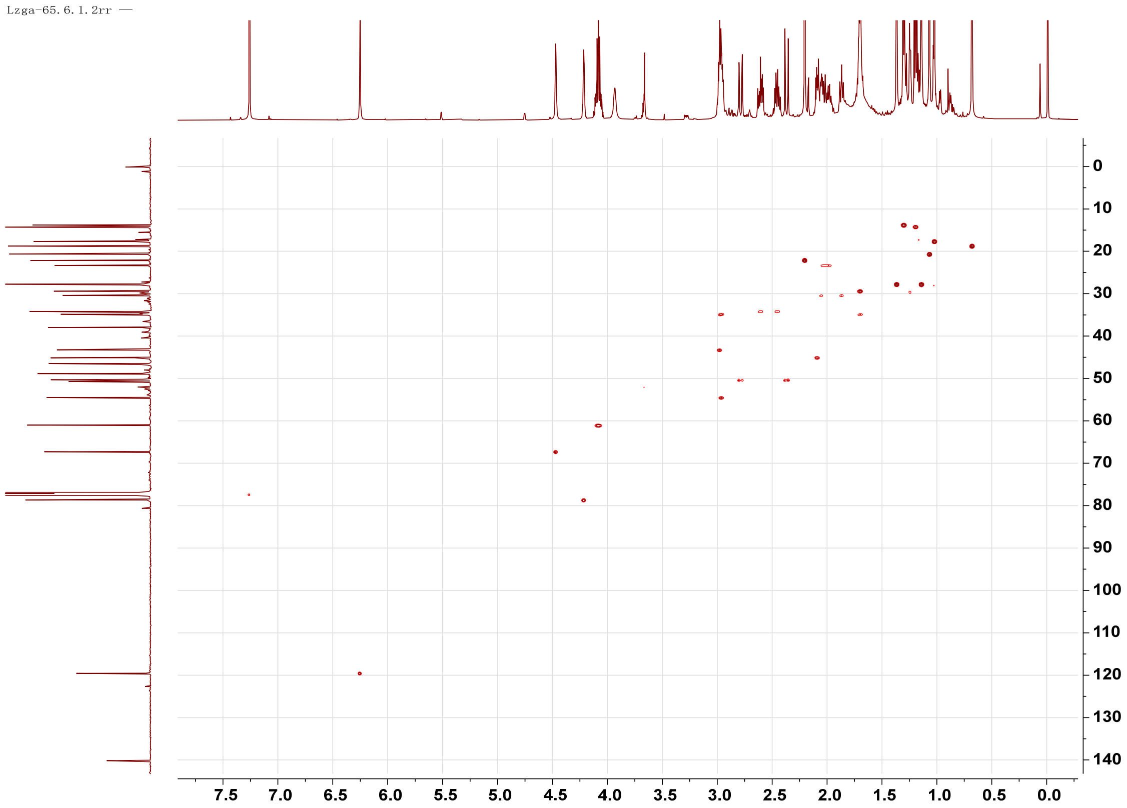

Figure S81. HMBC spectrum of **12**.

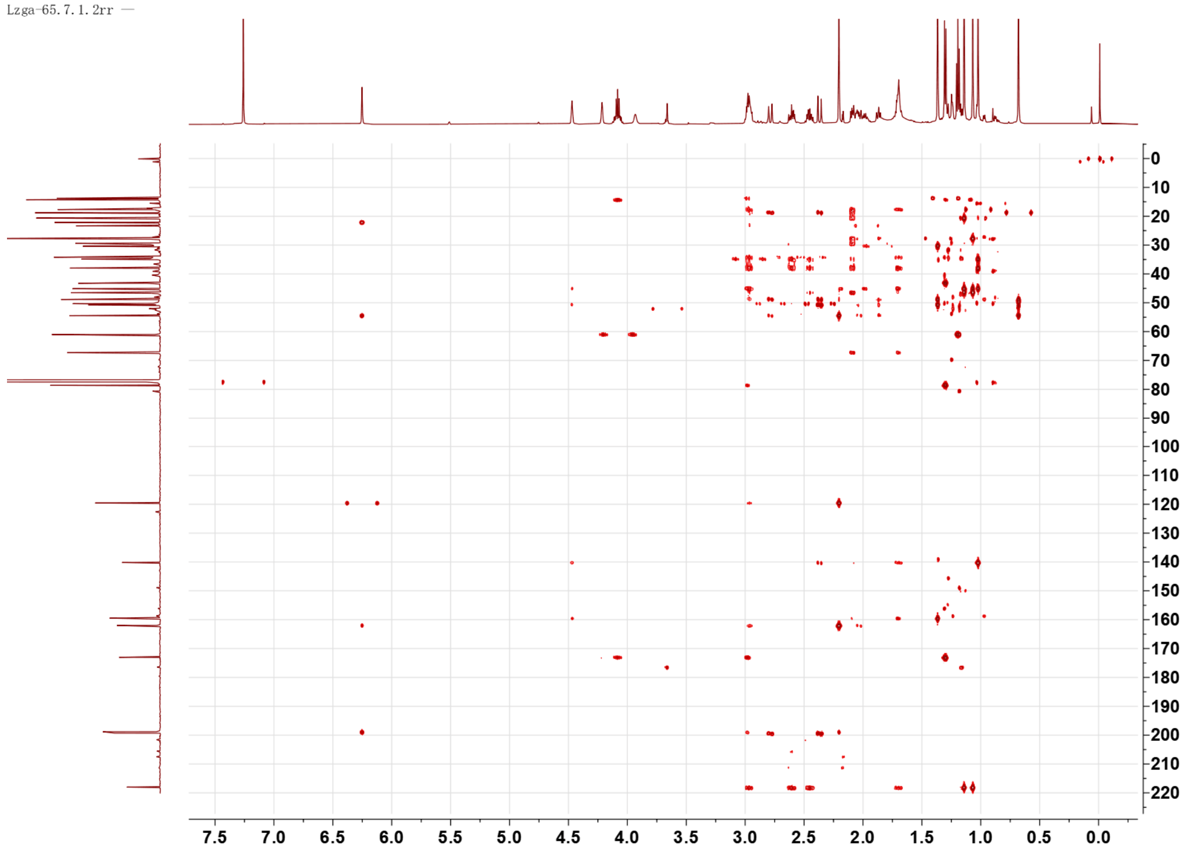

Figure S82. ^{1}H-^{1}H COSY spectrum of **12**.

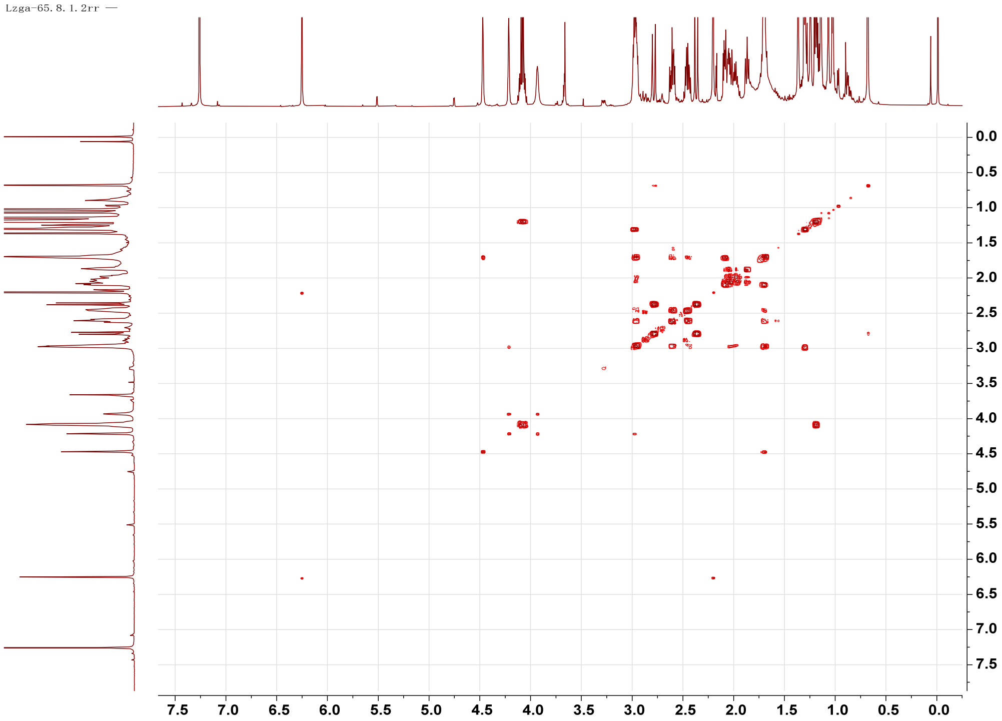

Figure S83. ROESY spectrum of **12**.

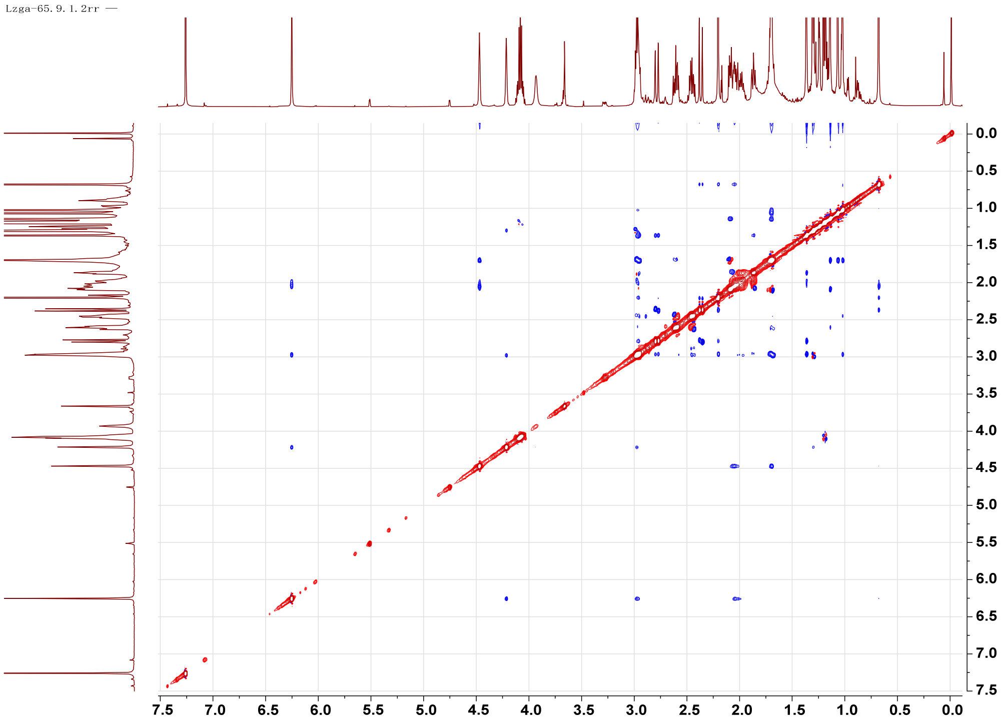

Figure S84. HRESIMS of **12**.

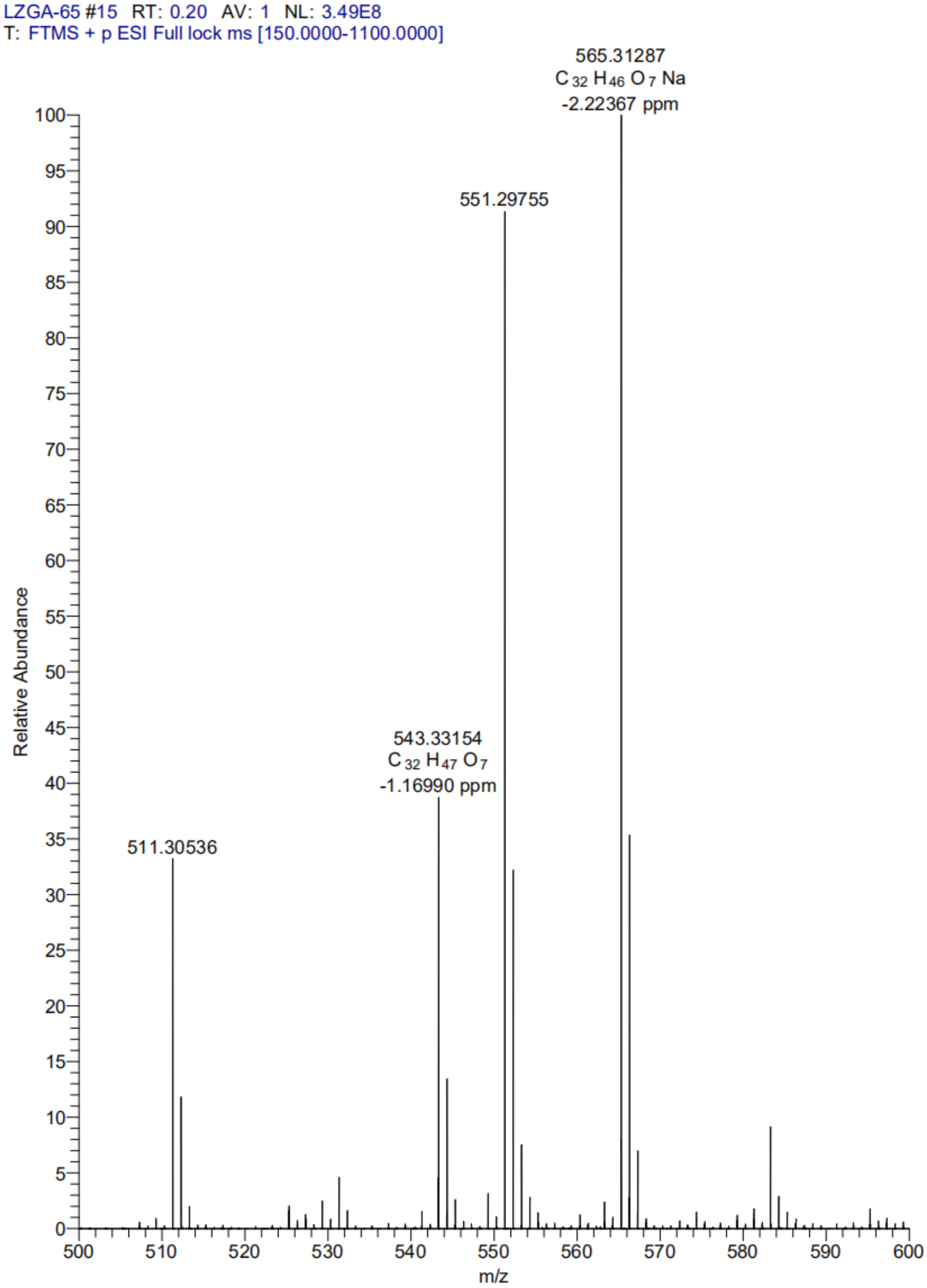

Figure S85. ^{1}H NMR spectrum of **13** (600 MHz, $CDCl_3$).

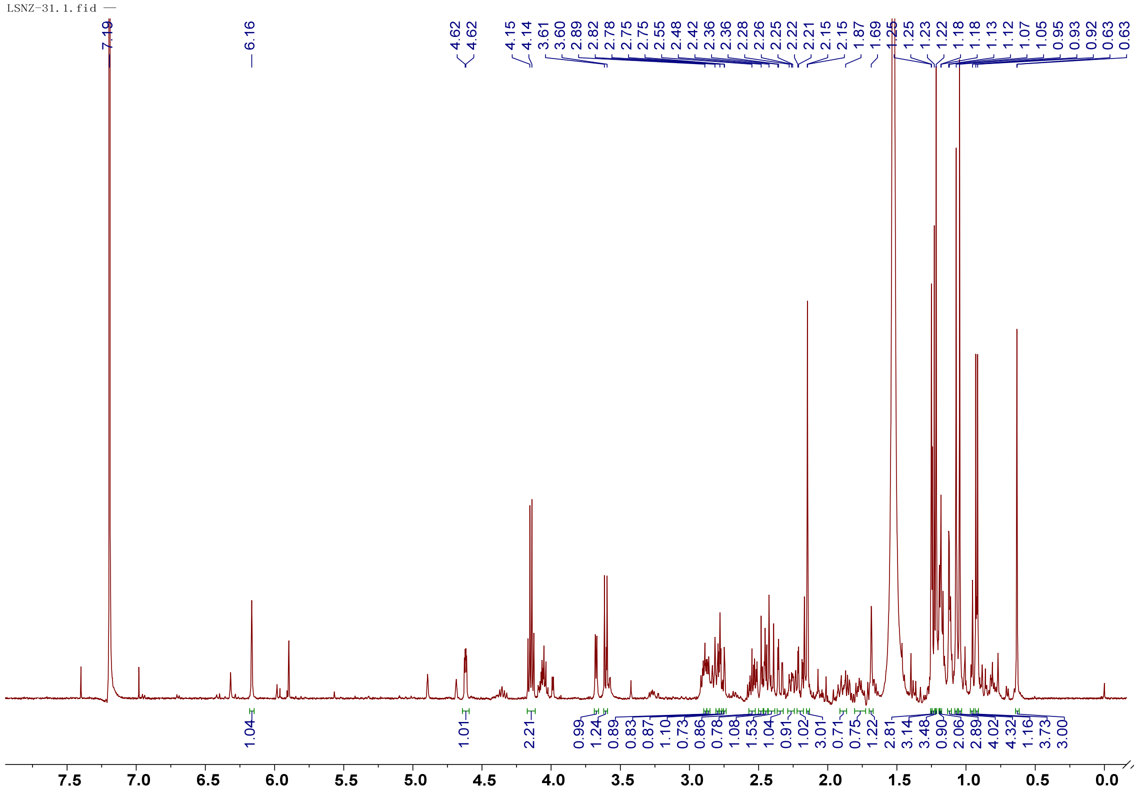

Figure S86. ^{13}C and DEPT NMR spectra of **13** (150 MHz, $CDCl_3$).

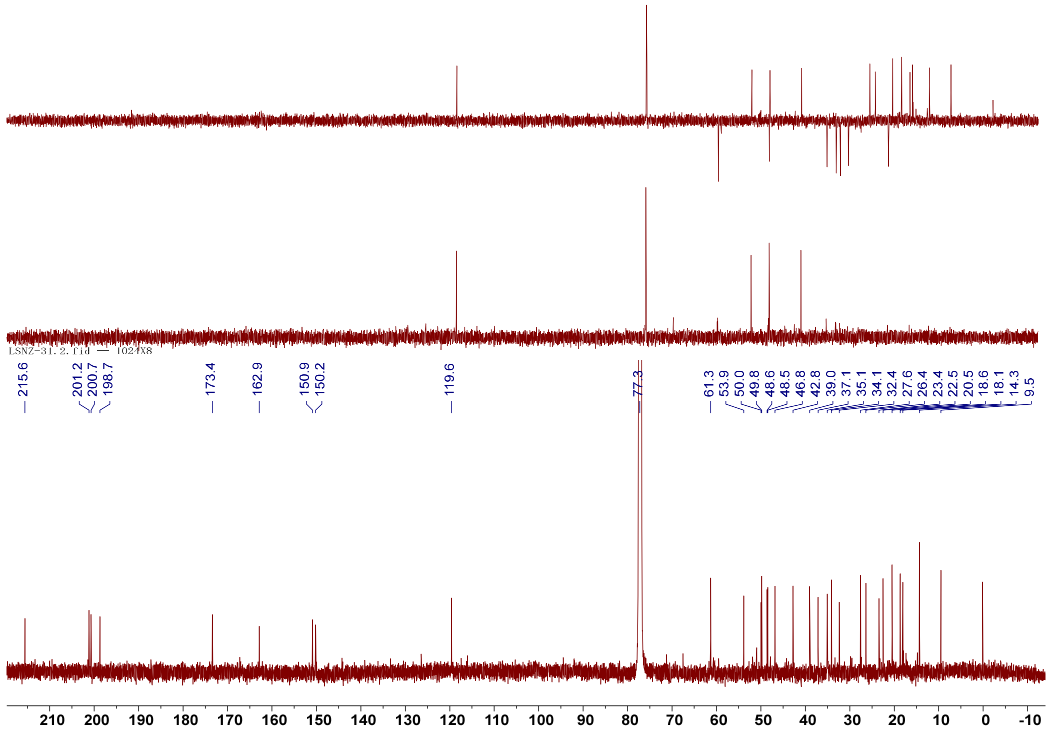

Figure S87. HSQC spectrum of **13**.

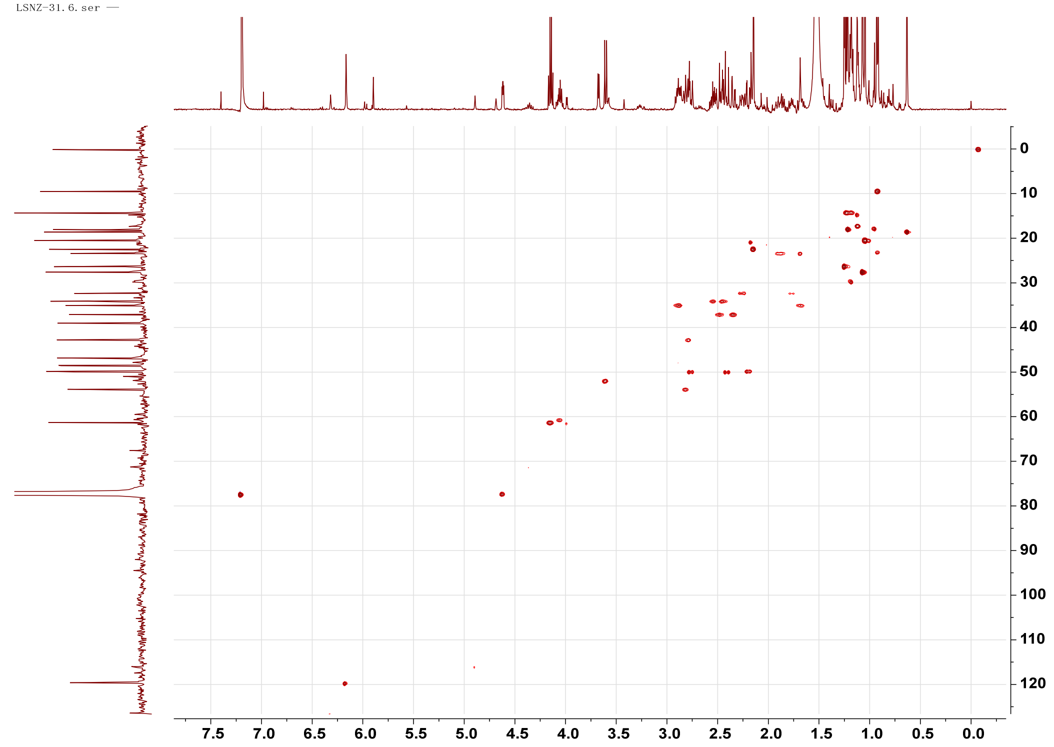

Figure S88. HMBC spectrum of **13**.

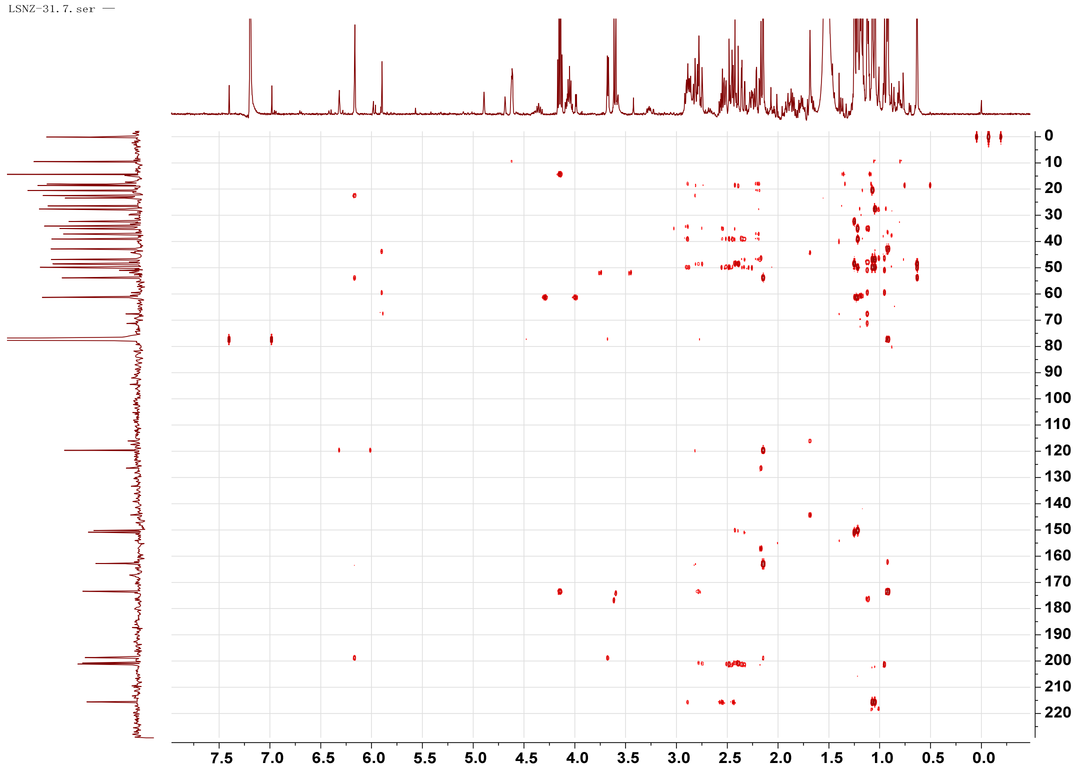

Figure S89. ^{1}H-^{1}H COSY spectrum of **13**.

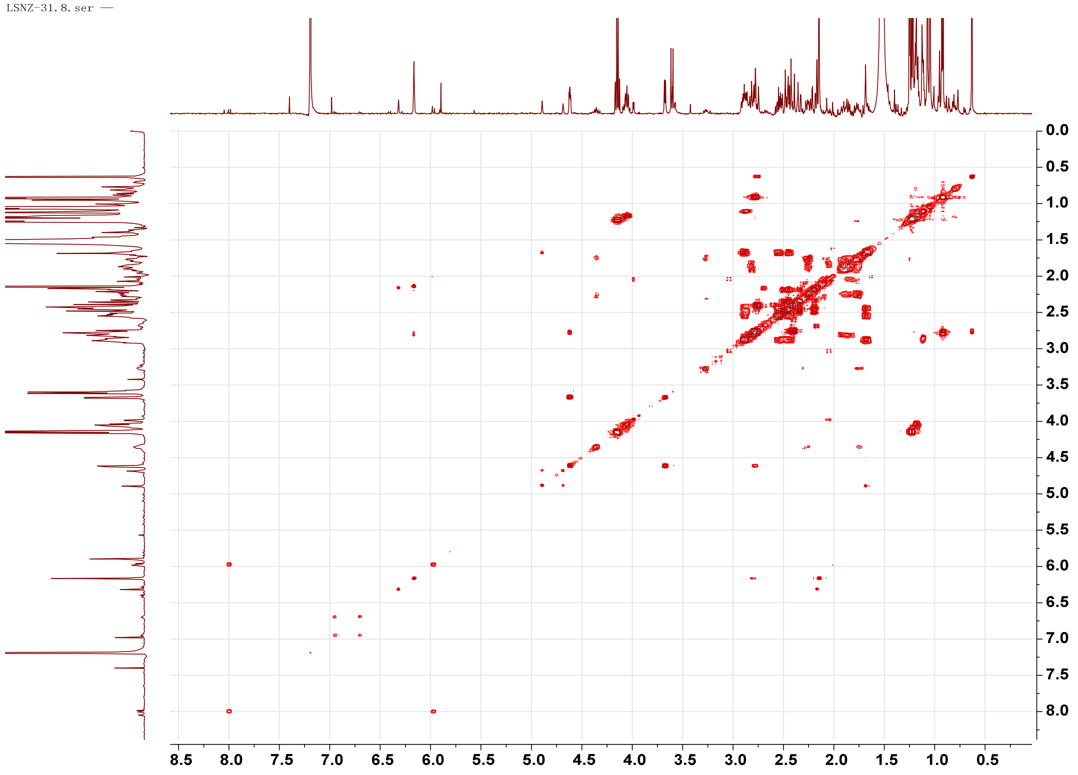

Figure S90. ROESY spectrum of **13**.

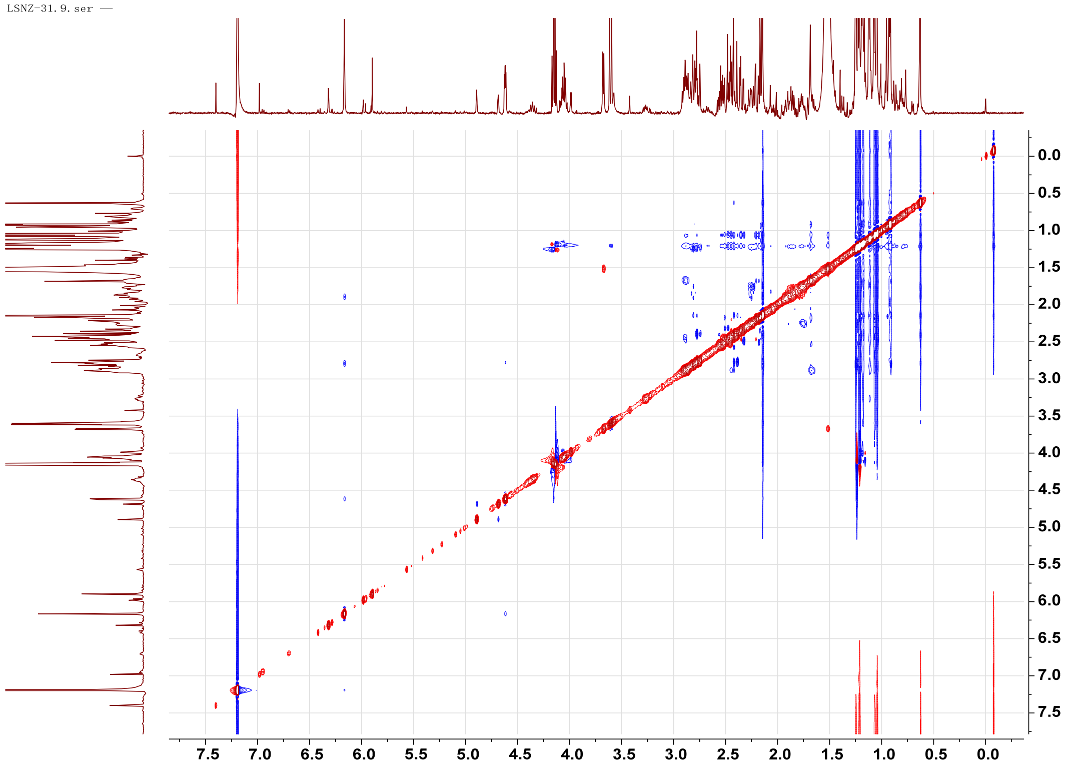

Figure S91. HRESIMS of **13**.

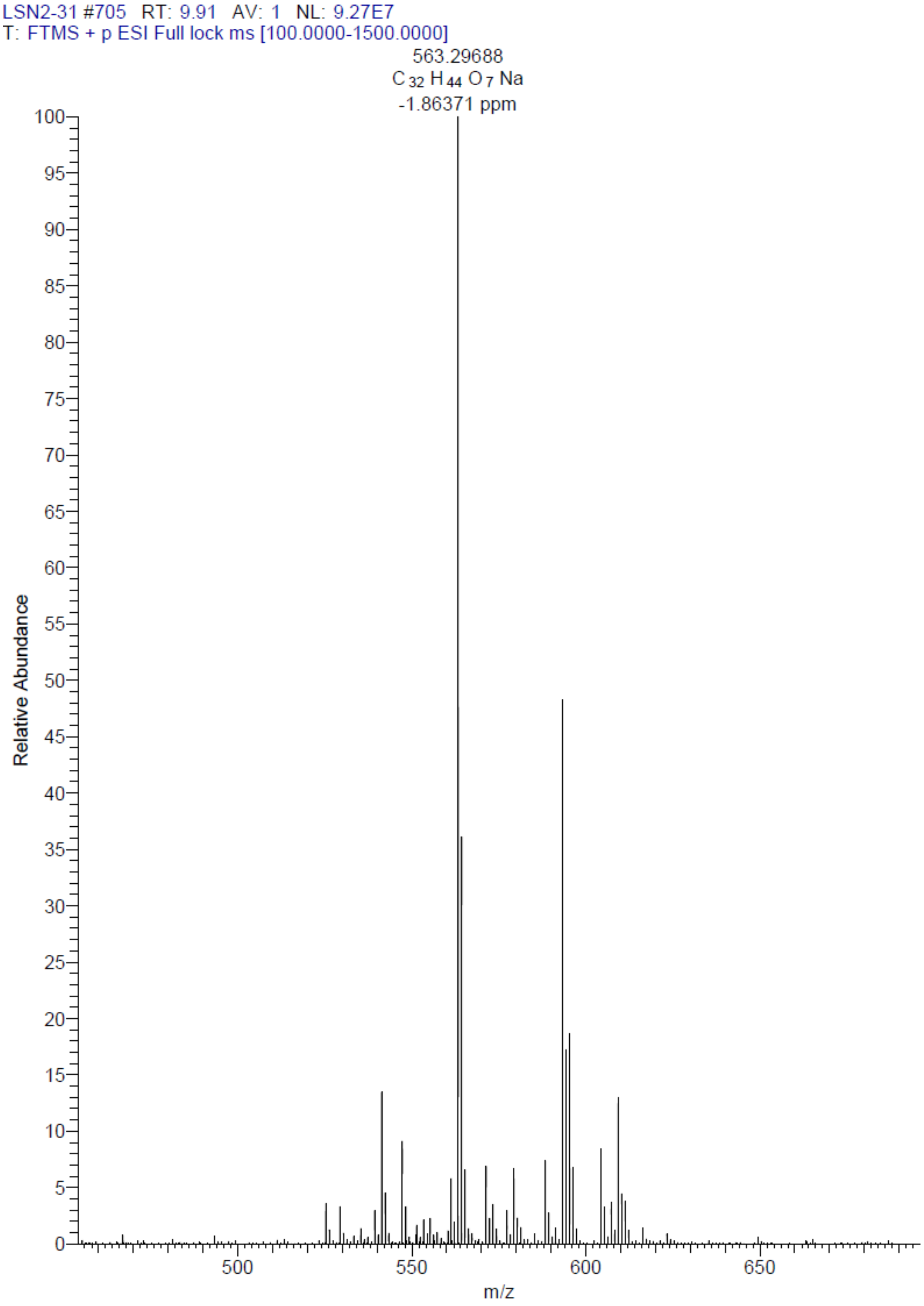

Figure S92. ^{1}H NMR spectrum of **1a** (600 MHz, $CDCl_3$).

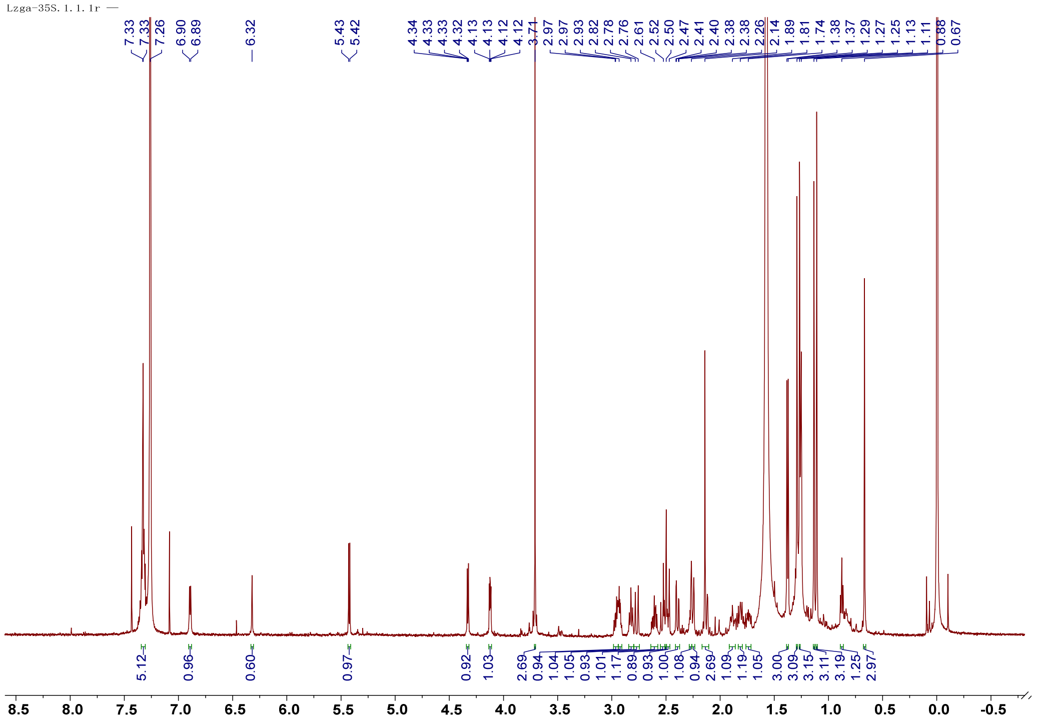

Figure S93. ^{1}H-^{1}H COSY spectrum of **1a**.

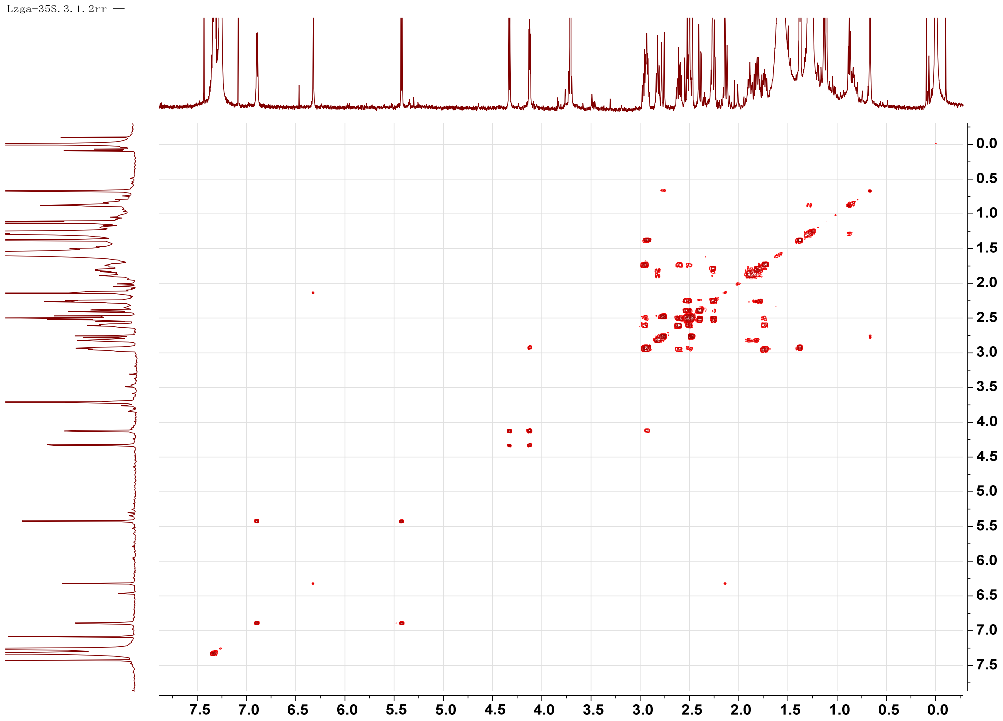

Figure S94. ^{1}H NMR spectrum of **1b** (600 MHz, $CDCl_3$).

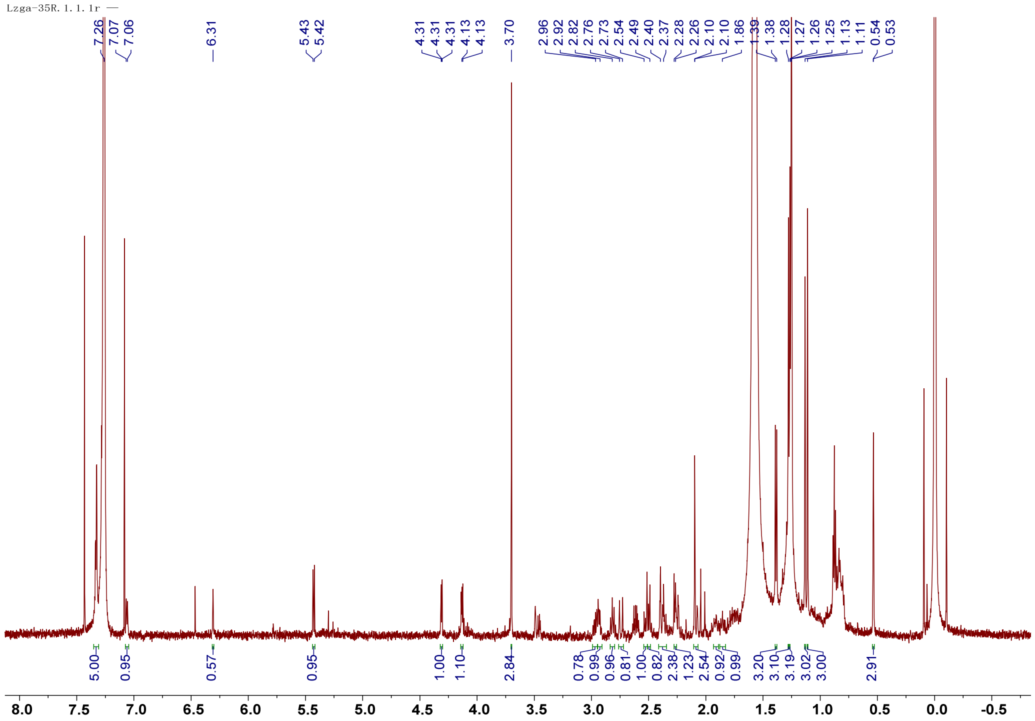

Figure S95. ^{1}H-^{1}H COSY spectrum of **1b**.

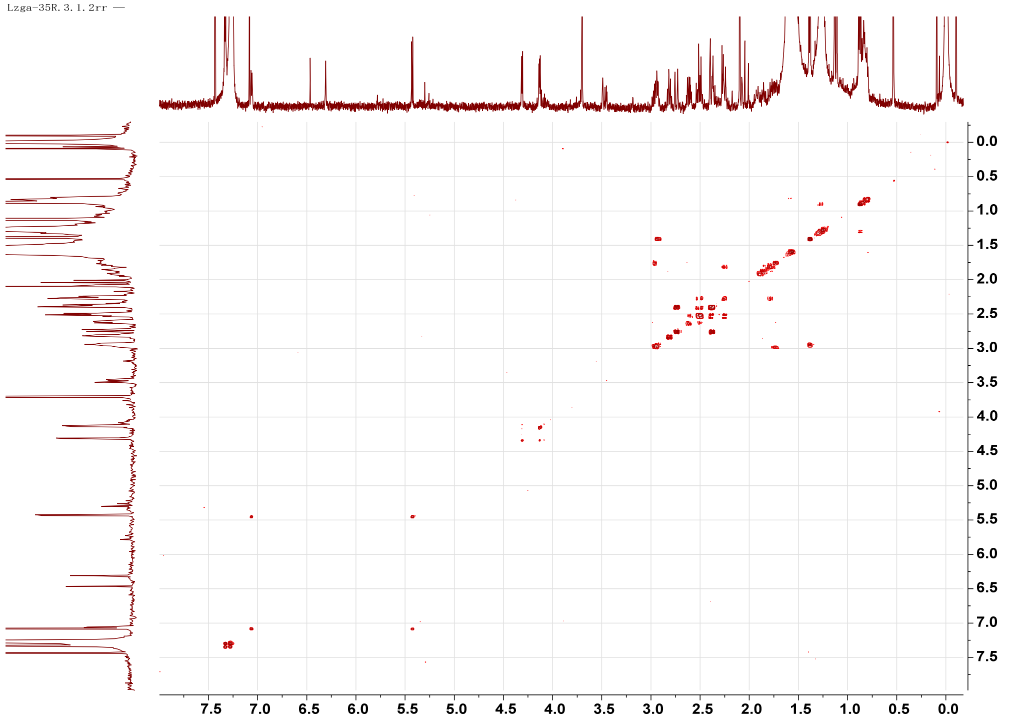

Figure S96. HRESIMS of **1a**.

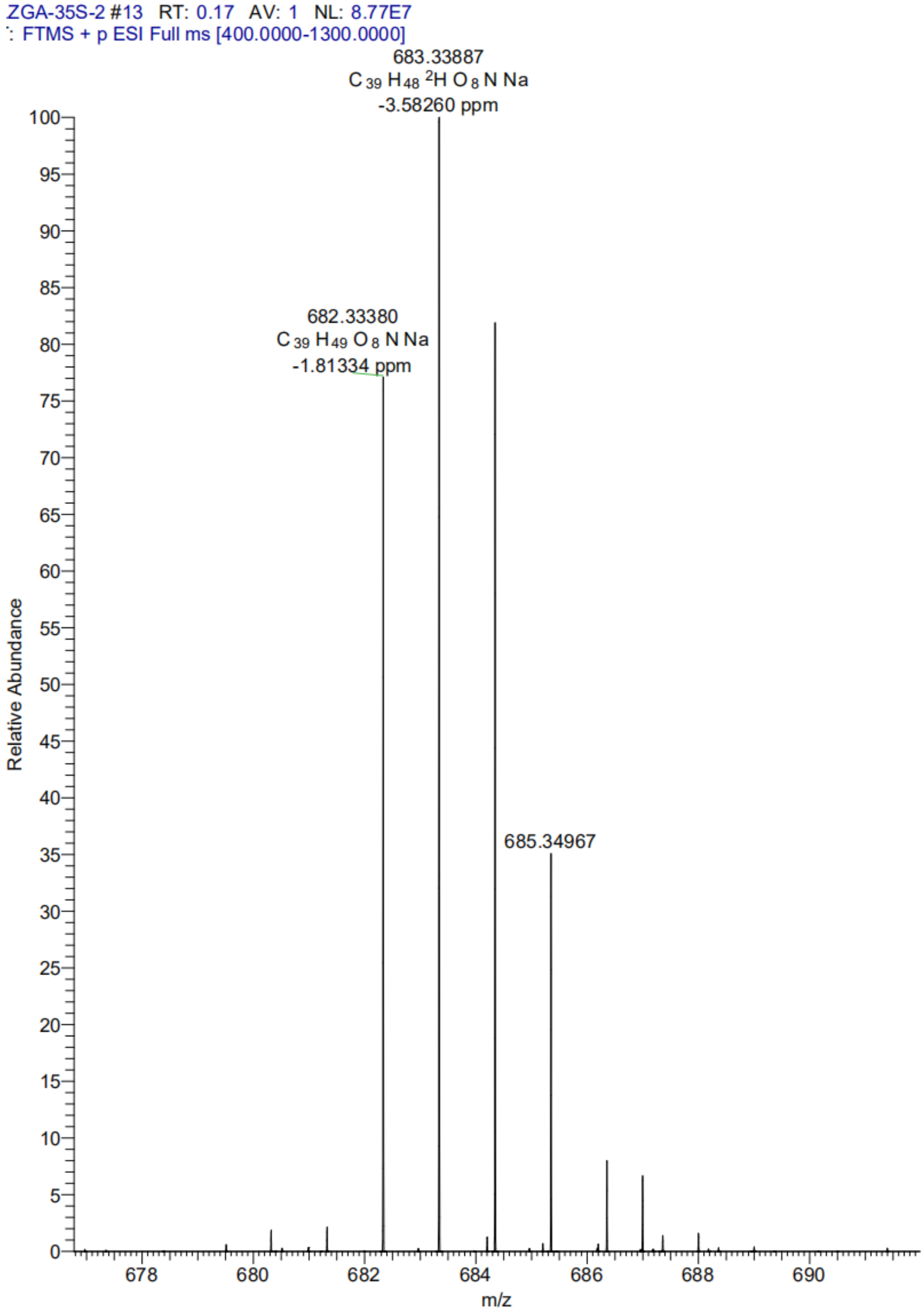

Figure S97. HRESIMS of **1b**.

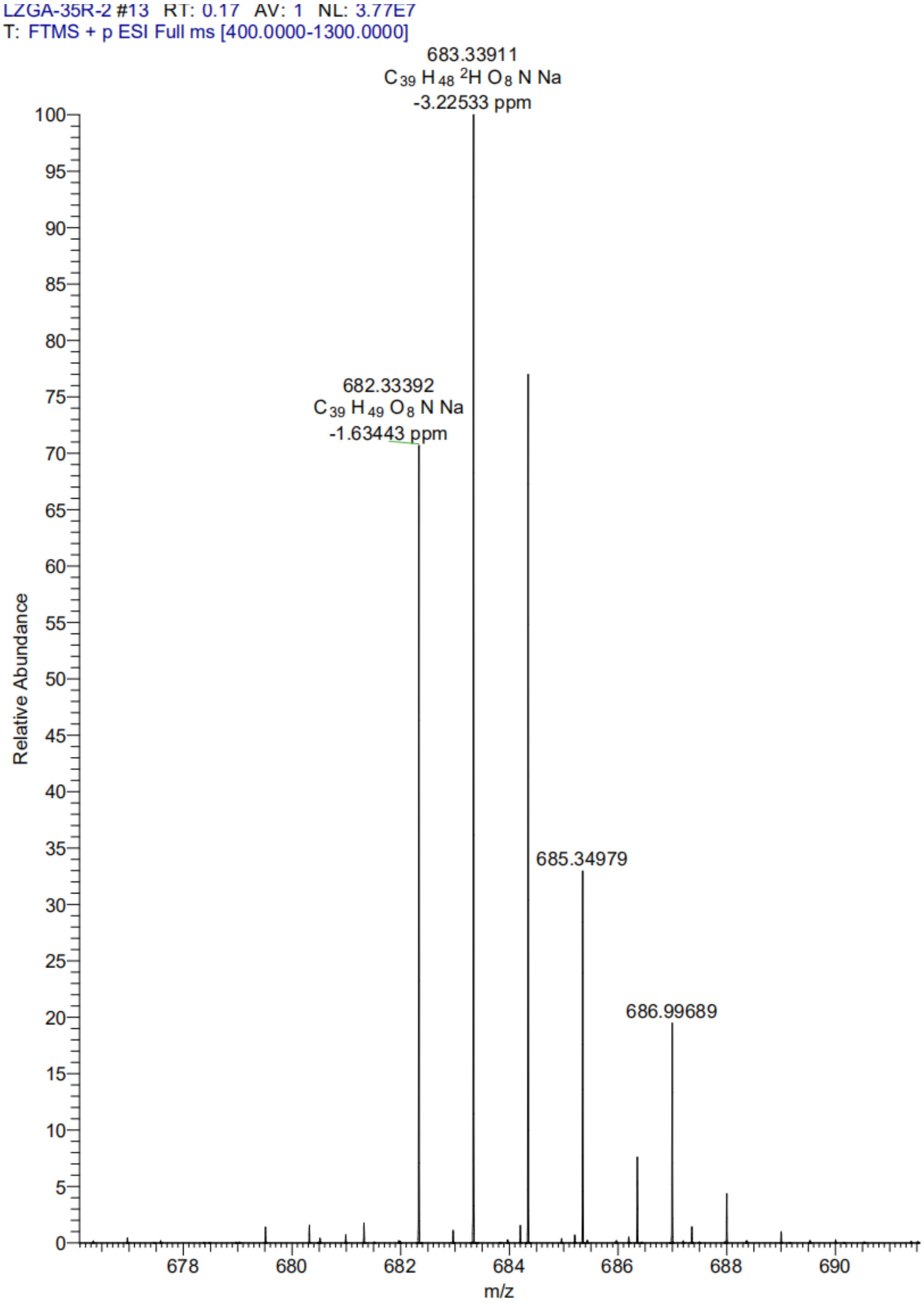

Figure S98. ^{1}H NMR spectrum of **2a** (600 MHz, $CDCl_3$).

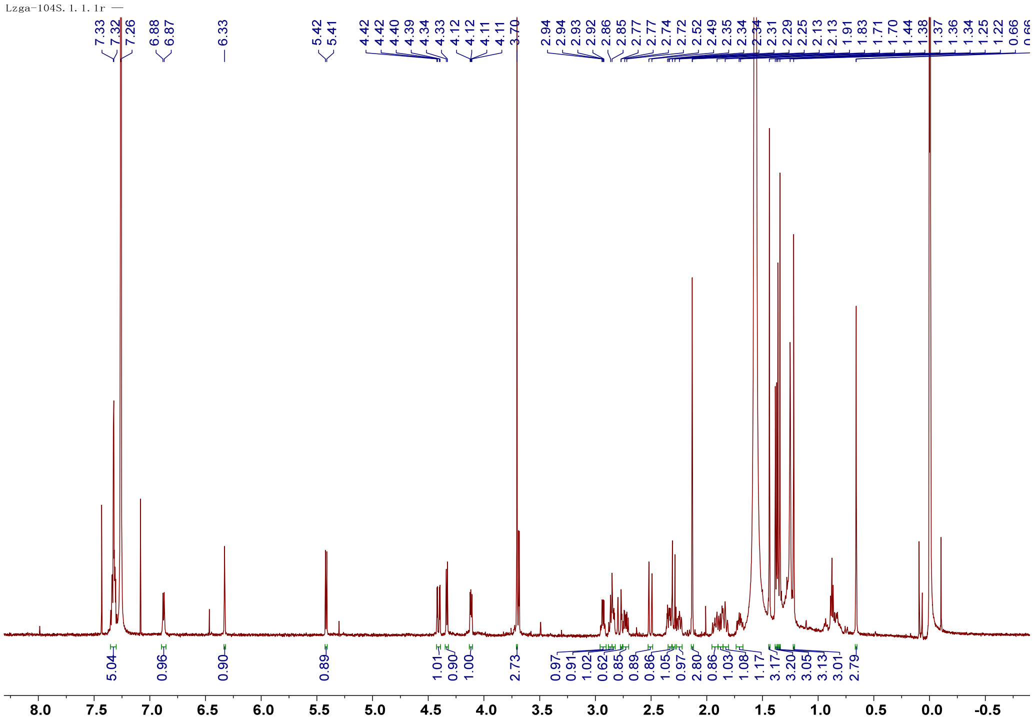

Figure S99. ^{1}H-^{1}H COSY spectrum of **2a**.

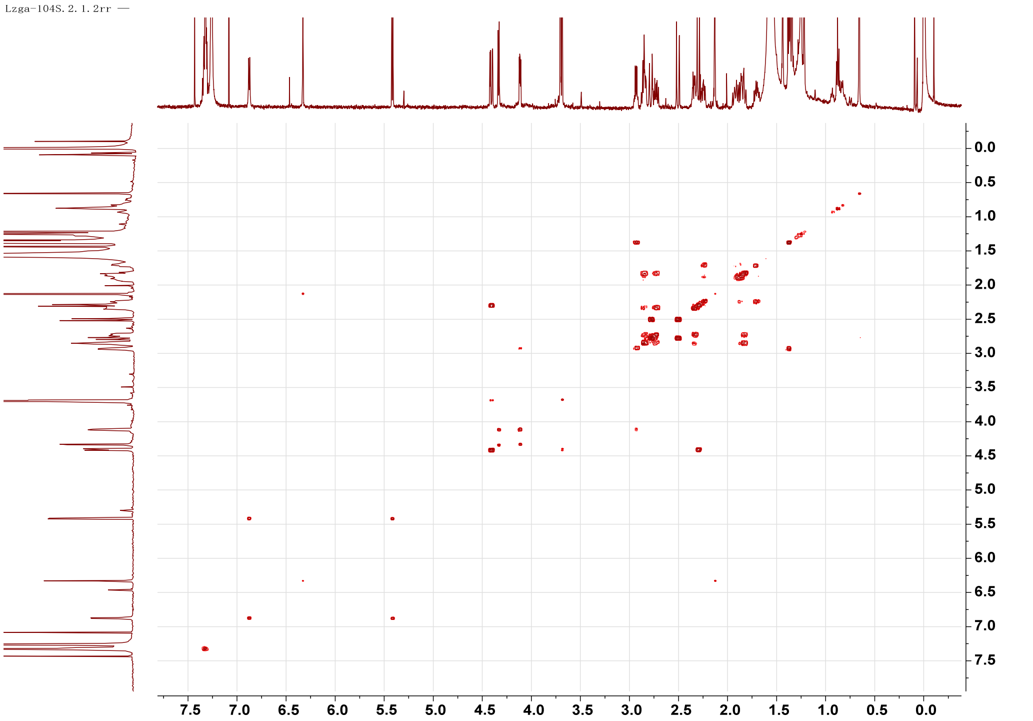

Figure S100. ^{1}H NMR spectrum of **2b** (600 MHz, $CDCl_3$).

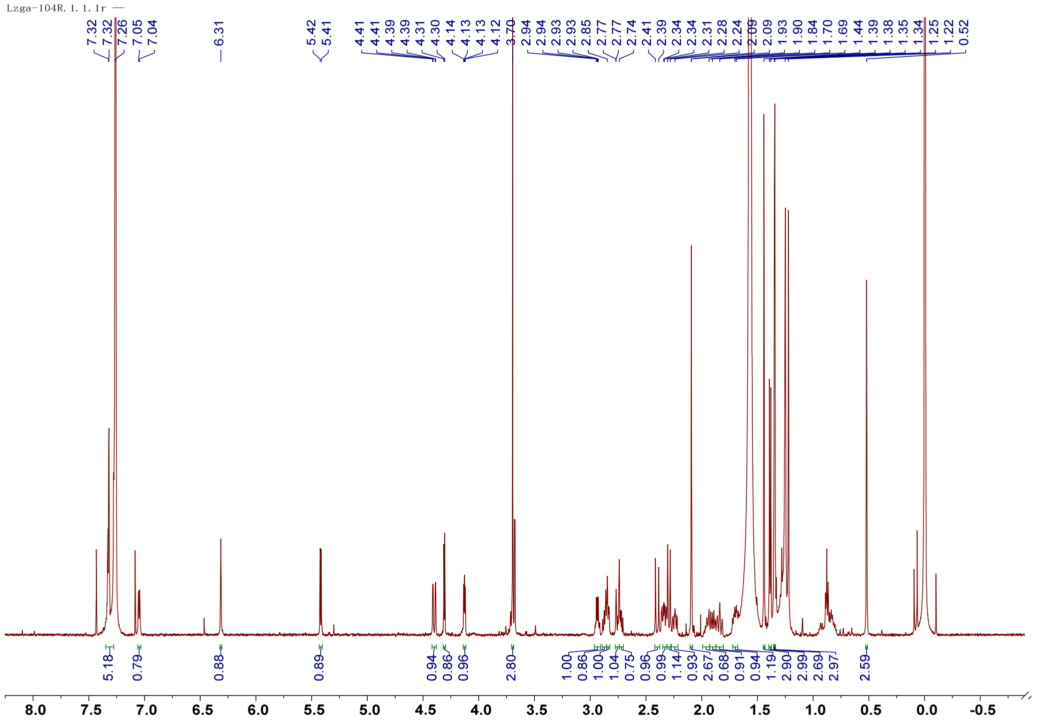

Figure S101. ^{1}H-^{1}H COSY spectrum of **2b**.

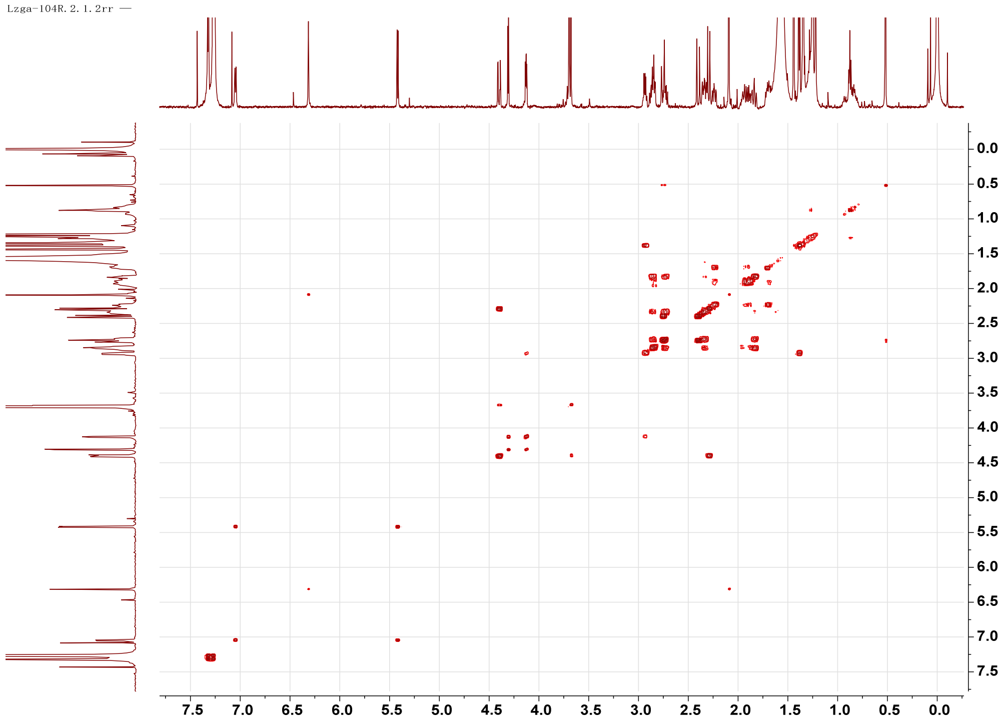

Figure S102. HRESIMS of **2a**.

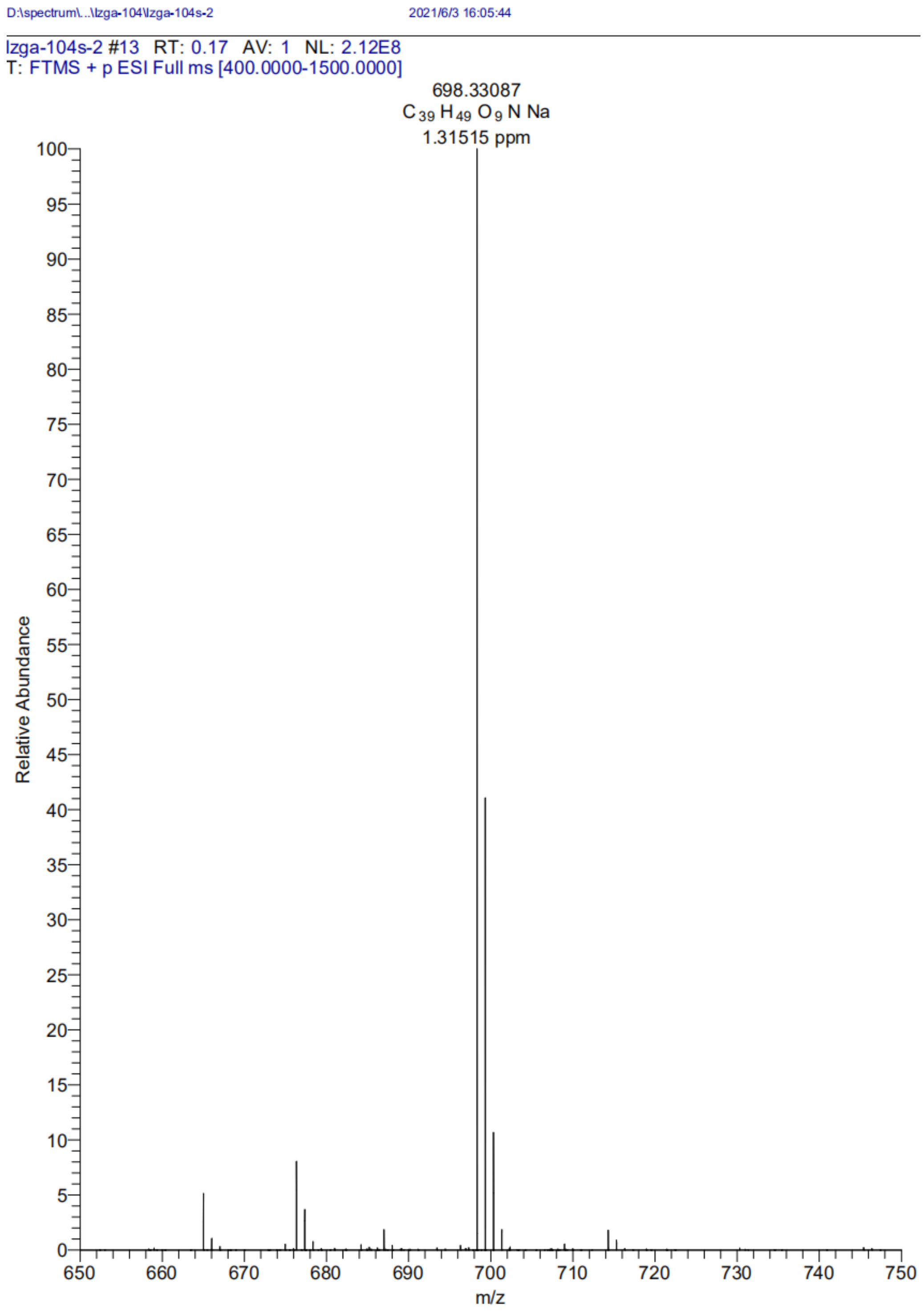

Figure S103. HRESIMS of **2b**.

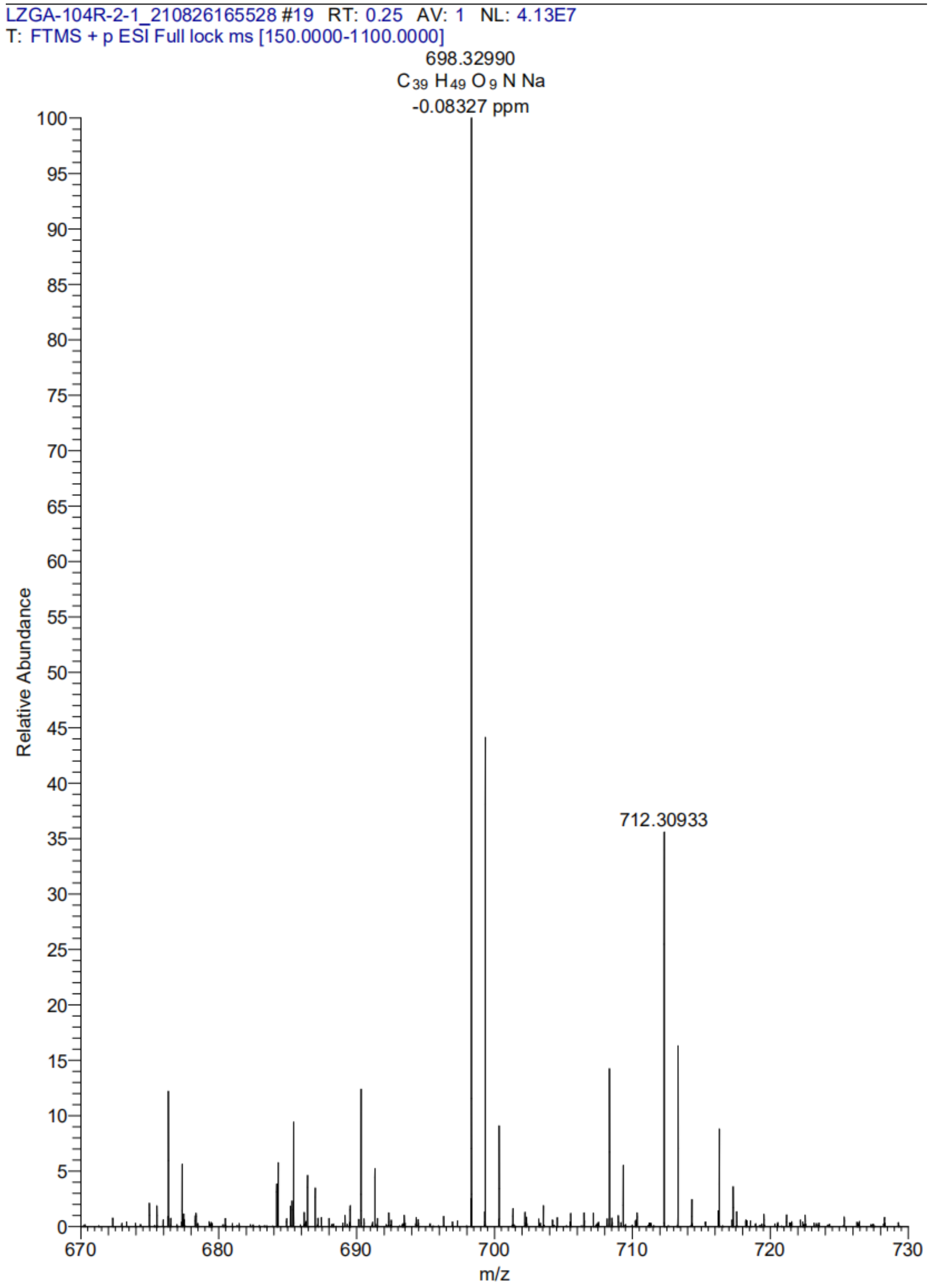

Figure S104. ^{1}H NMR spectrum of **3a** (600 MHz, $CDCl_3$).

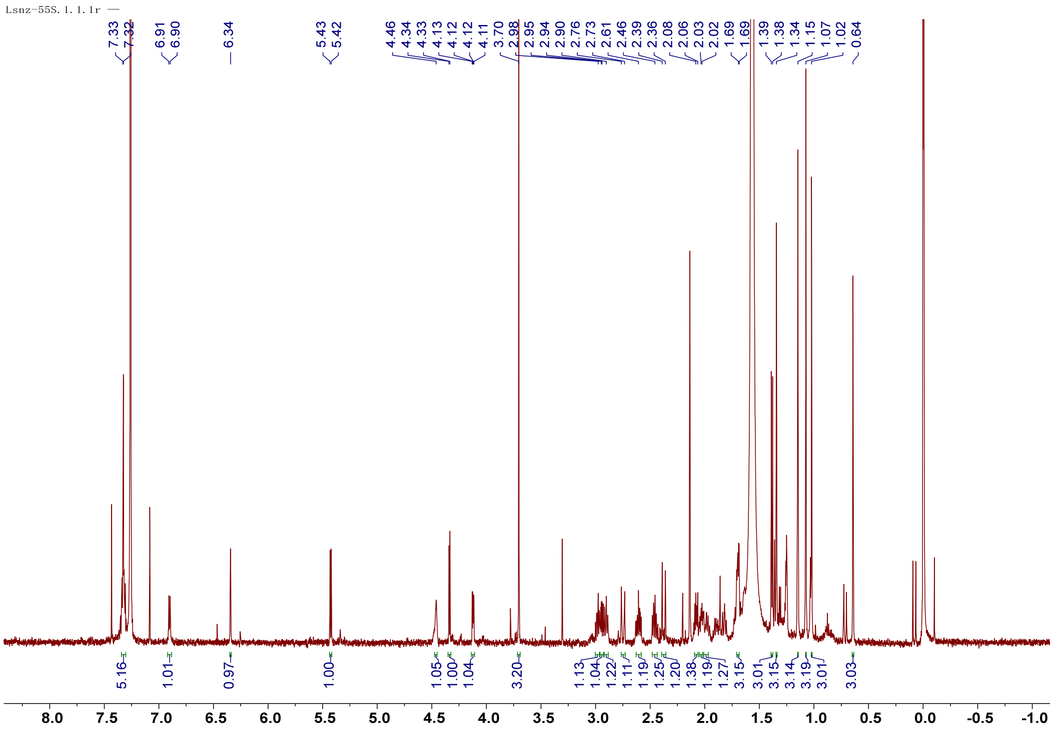

Figure S105. ^{1}H-^{1}H COSY spectrum of **3a**.

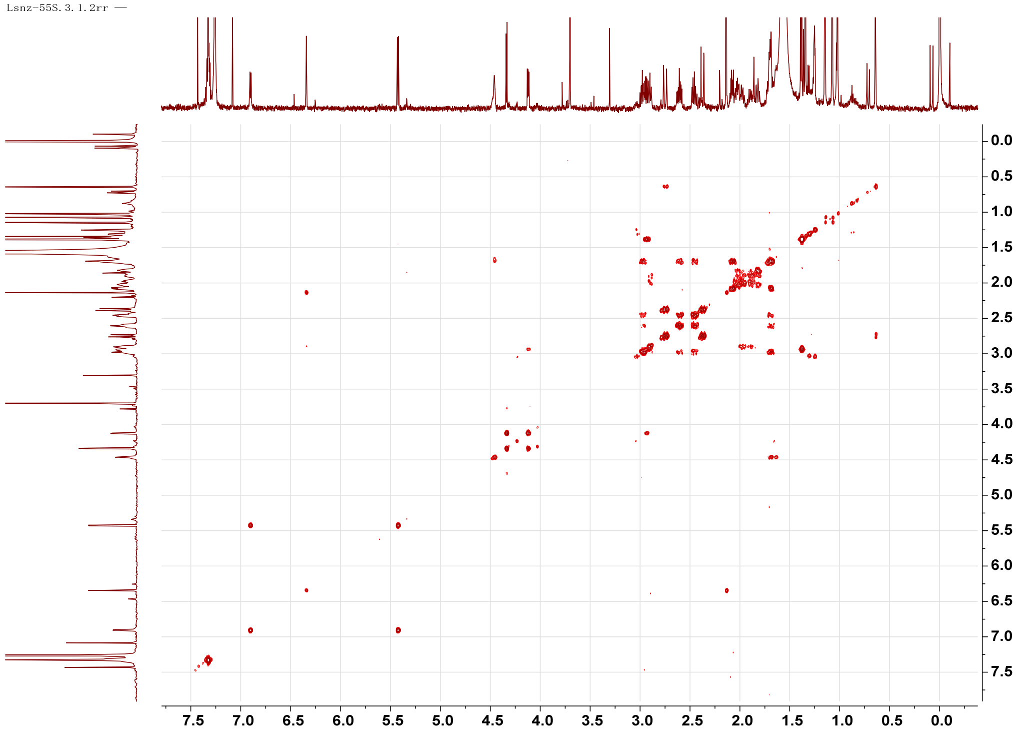

Figure S106. ^{1}H NMR spectrum of **3b** (600 MHz, $CDCl_3$).

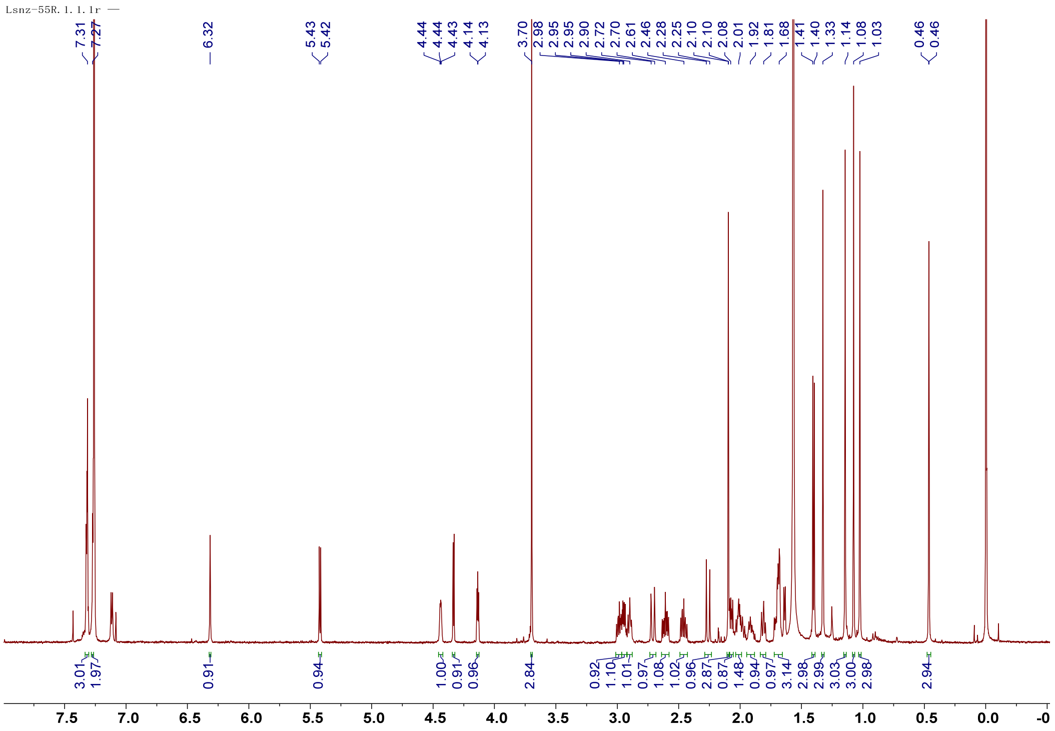

Figure S107. ^{1}H-^{1}H COSY spectrum of **3b**.

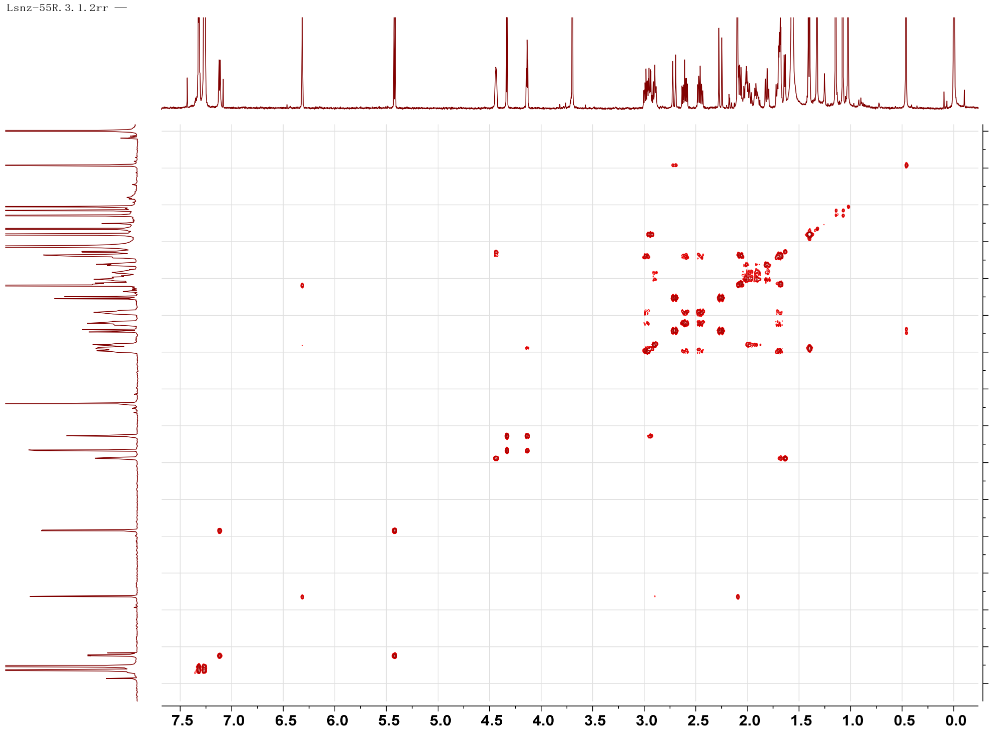

Figure S108. HRESIMS of **3a**.

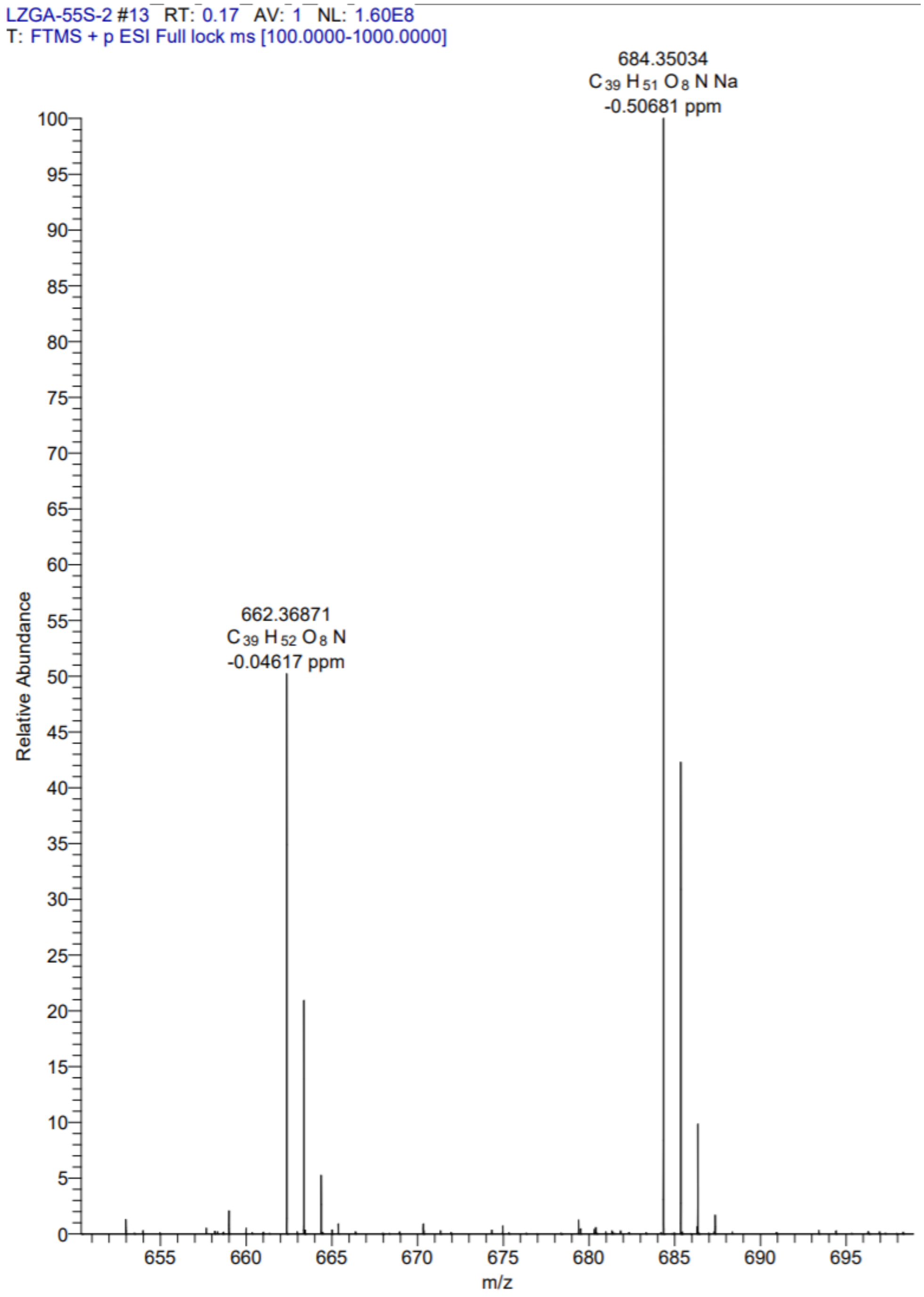

Figure S109. HRESIMS of **3b**.

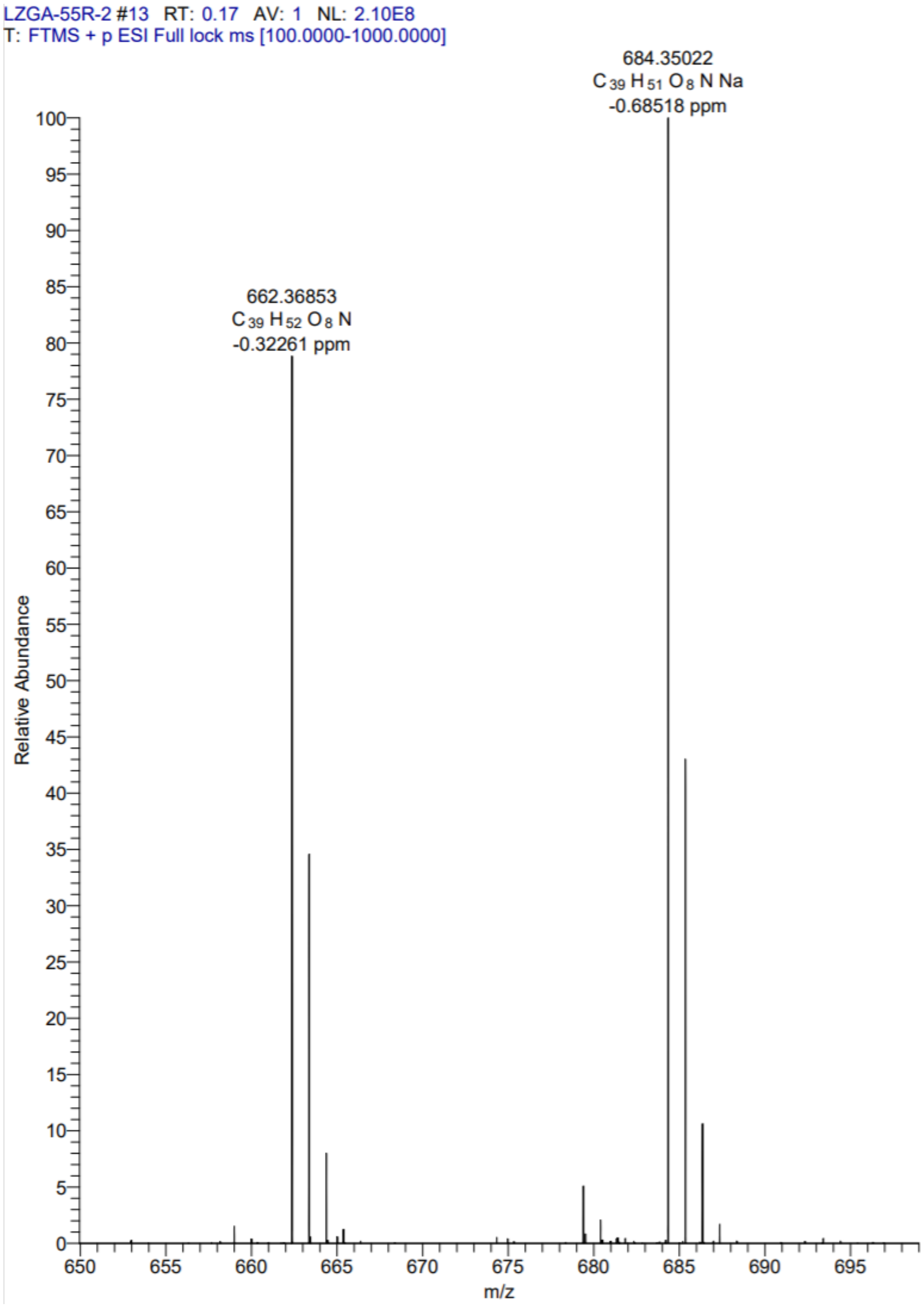

Figure S110. ^{1}H NMR spectrum of **6H** (600 MHz, $CDCl_3$).

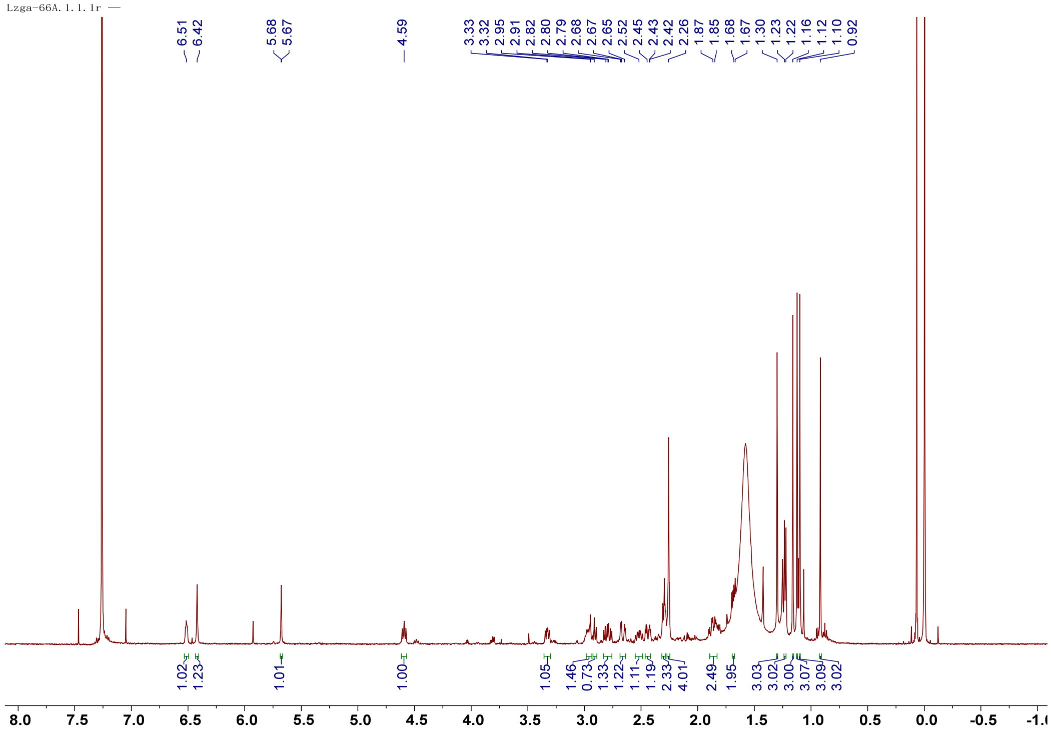

Figure S111. ^{13}C NMR spectrum of **6H** (600 MHz, $CDCl_3$).

Number of scans= 18000

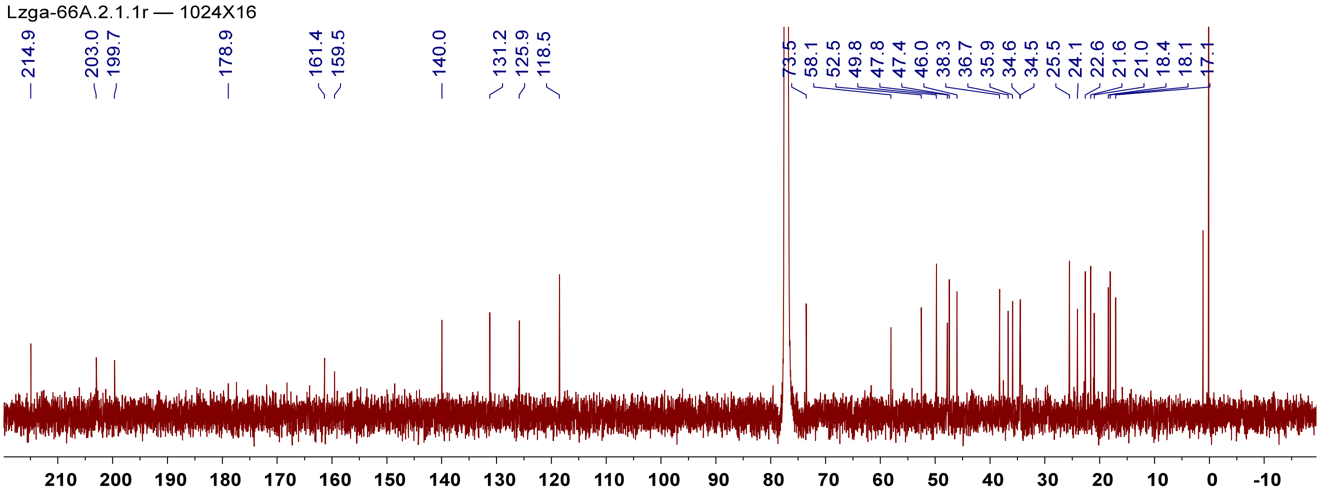

Figure S112. ^{1}H NMR spectrum of **6Ha** (600 MHz, $CDCl_3$).

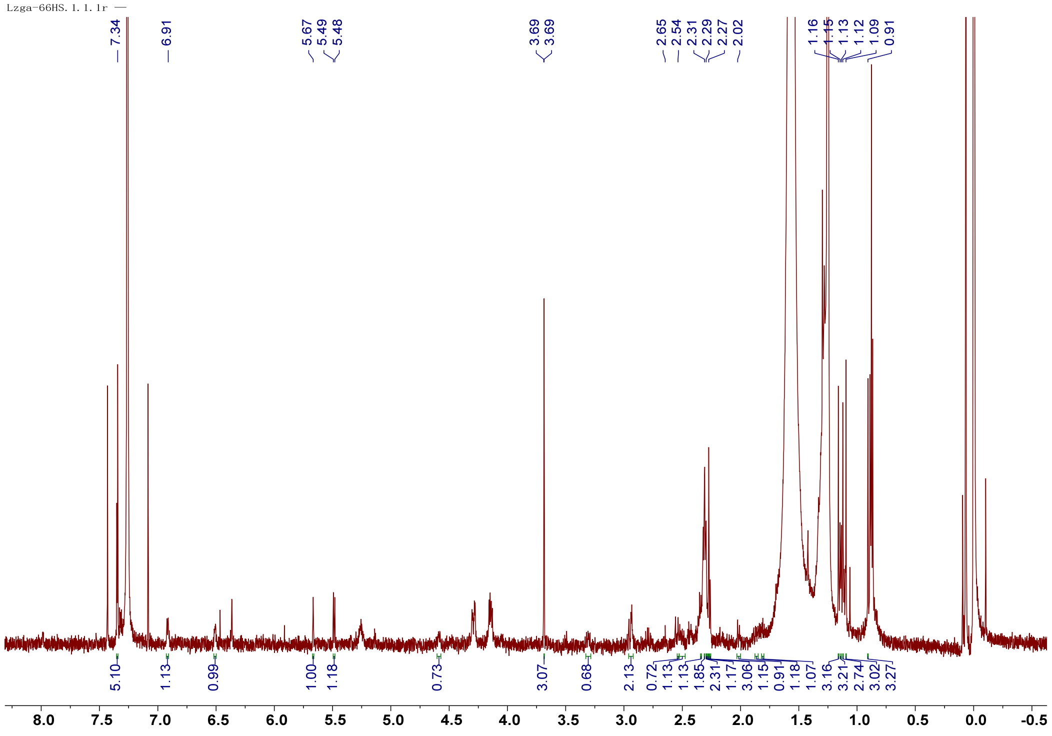

Figure S113. ^{1}H-^{1}H COSY spectrum of **6Ha**.

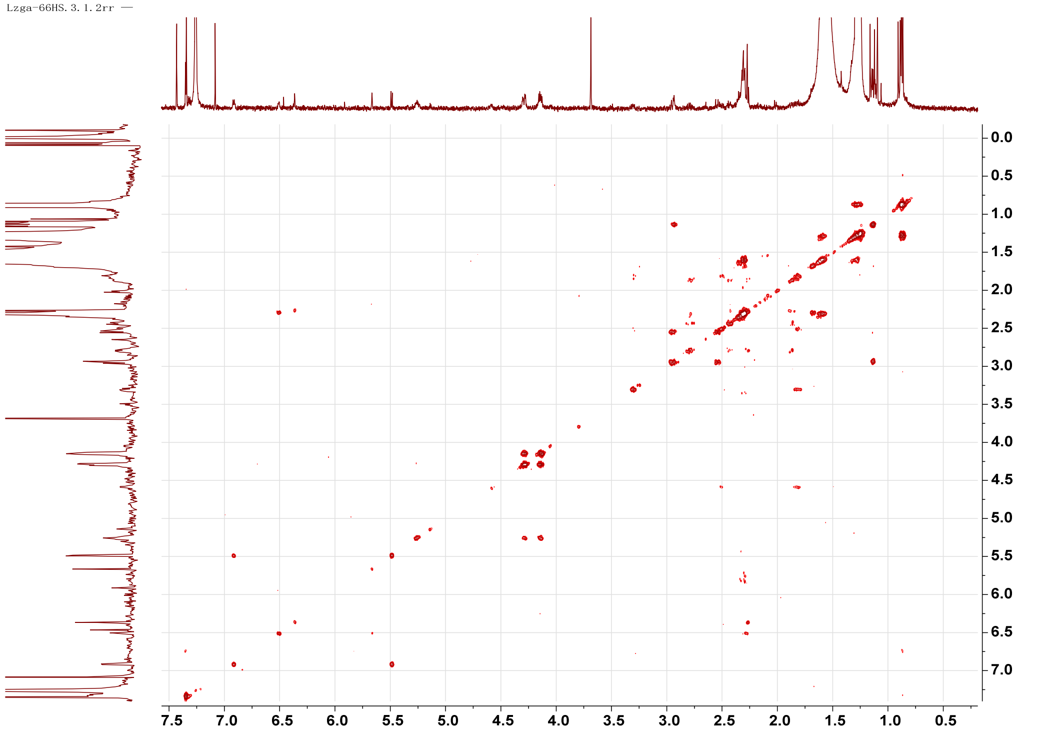

Figure S114. ^{1}H NMR spectrum of **6Hb** (600 MHz, $CDCl_3$).

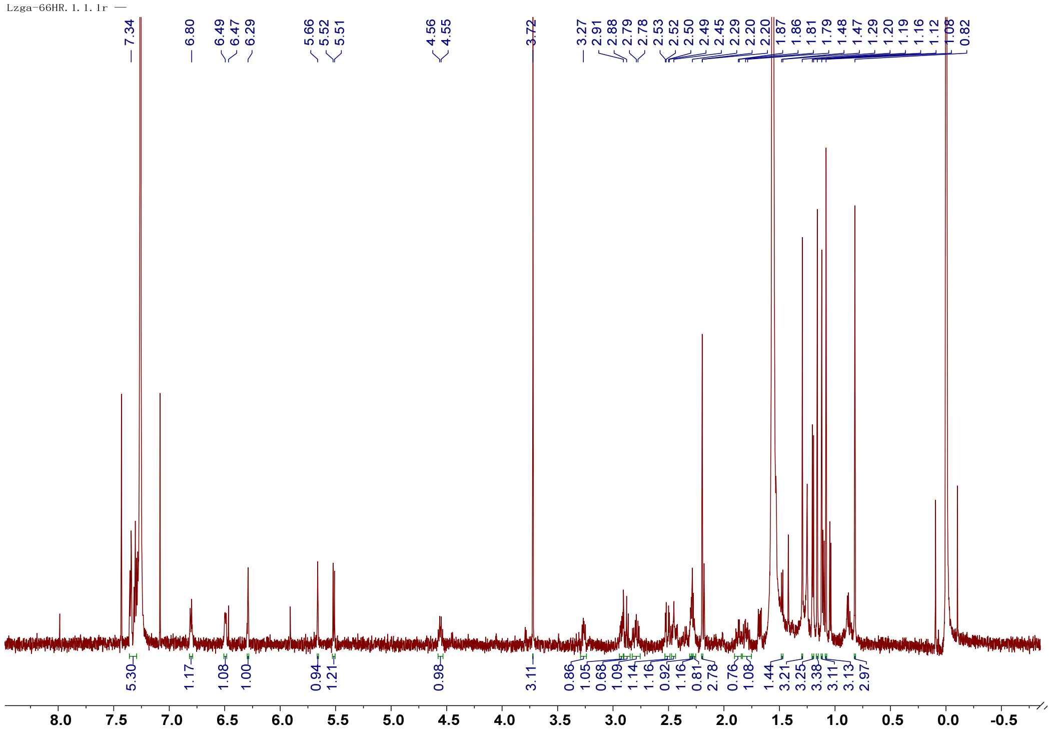

Figure S115. ^{1}H-^{1}H COSY spectrum of **6Hb**.

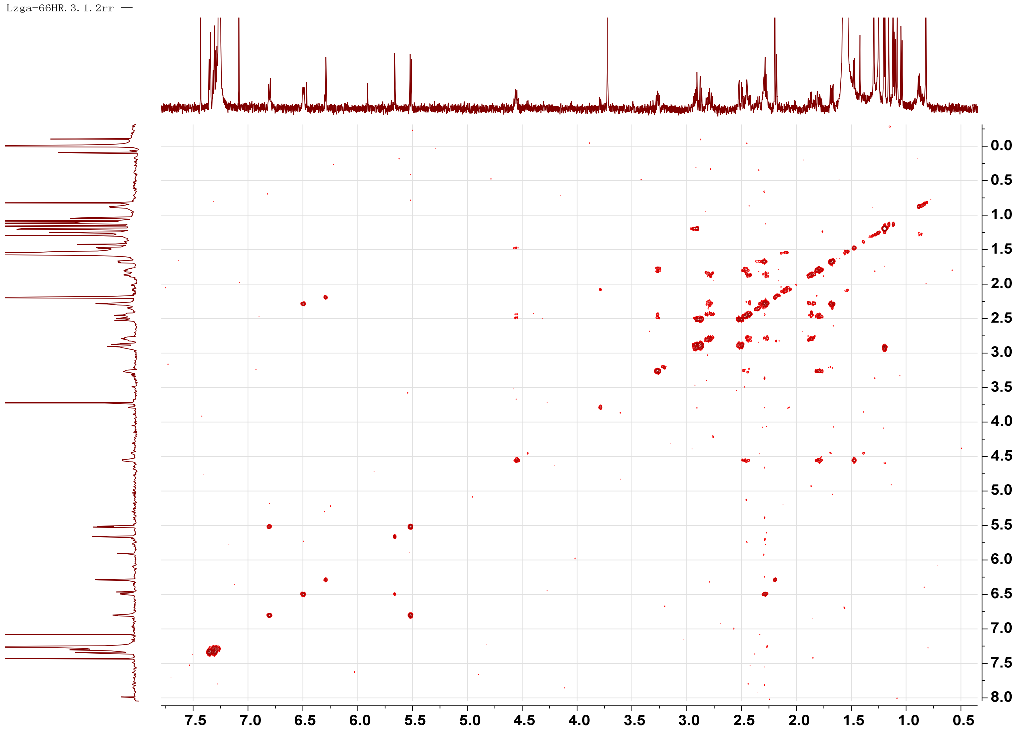

Figure S116. HRESIMS of **6Ha**.

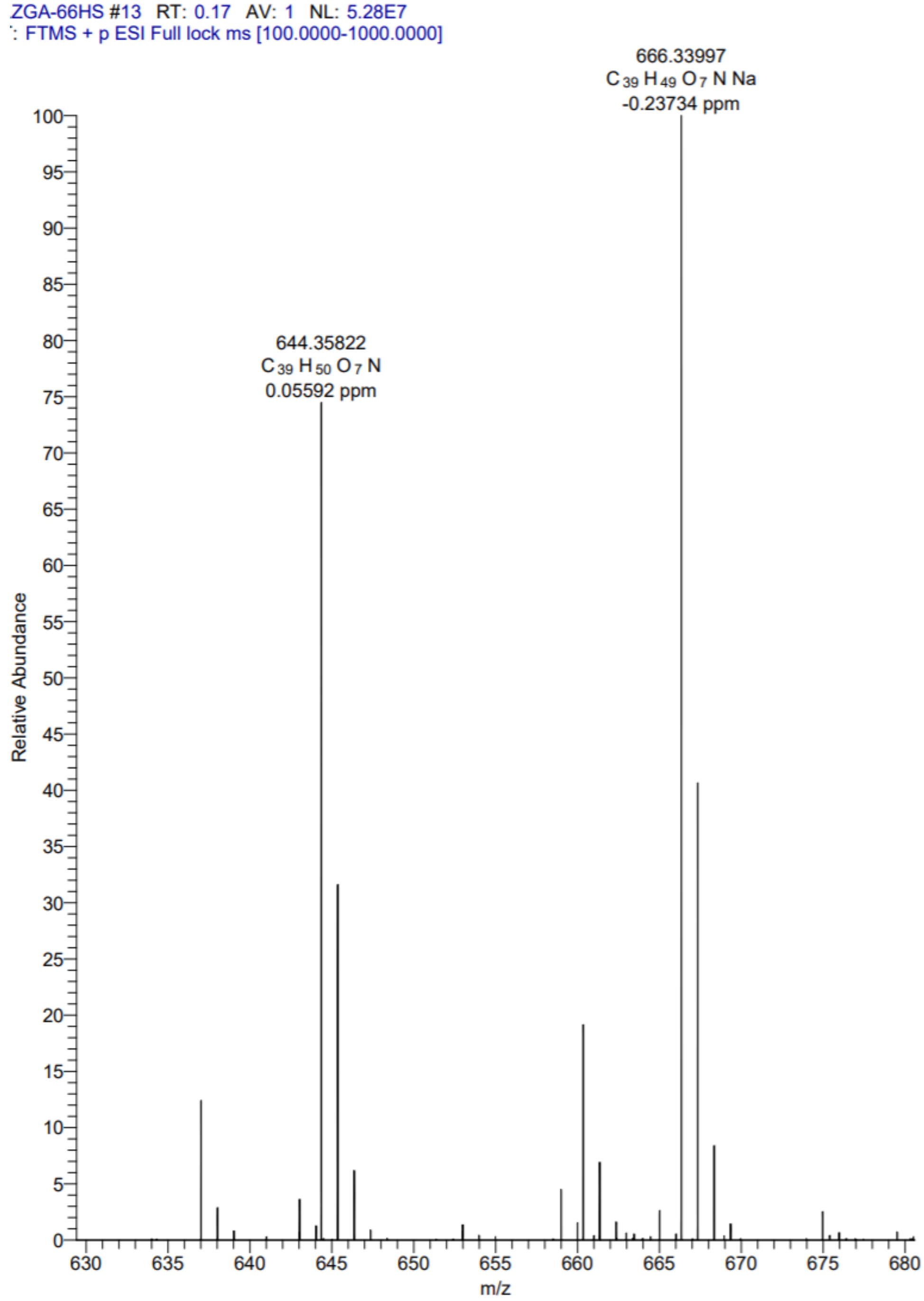

Figure S117. HRESIMS of **6Hb**.

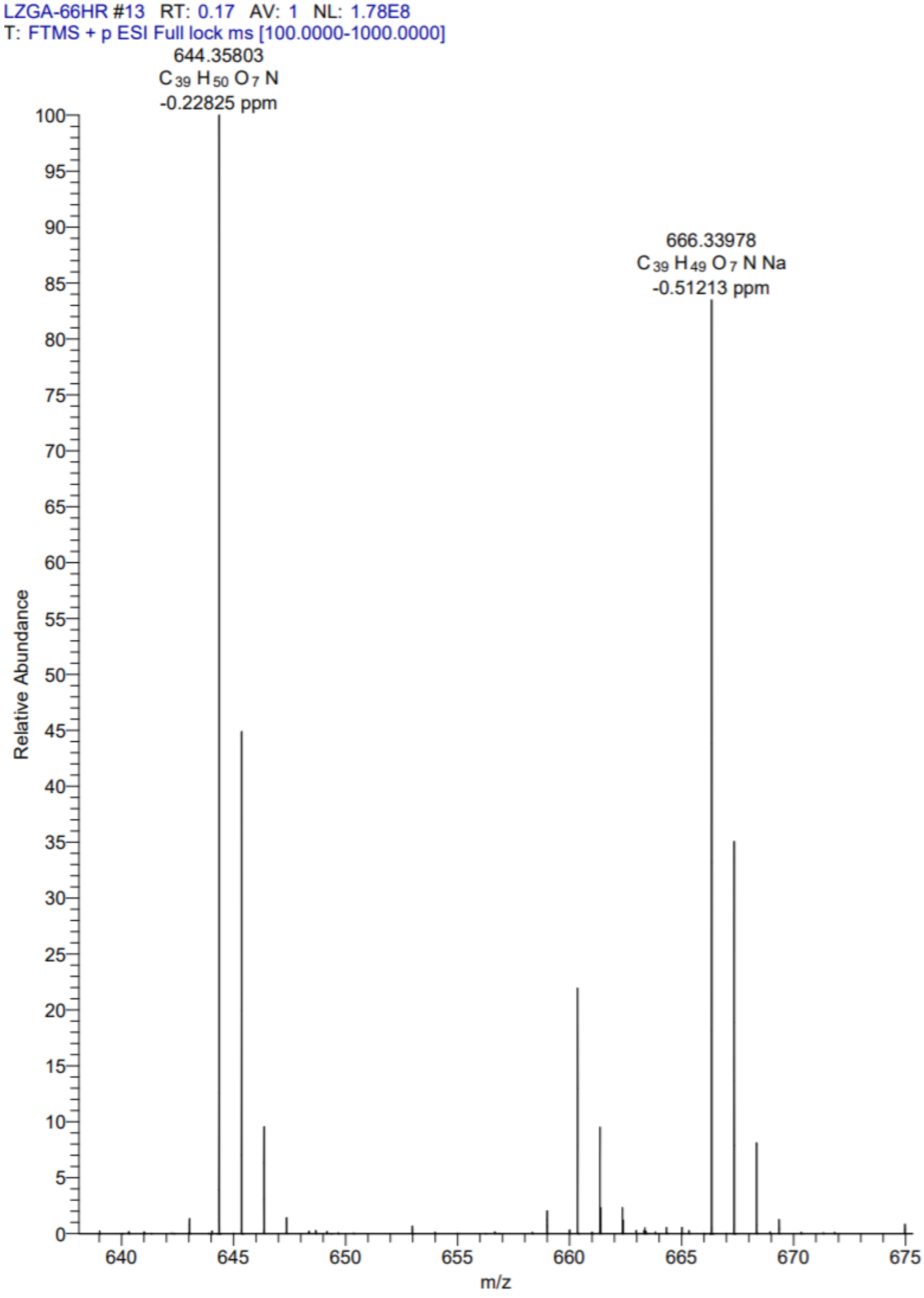

2. **Spectroscopic data of compound 6.**

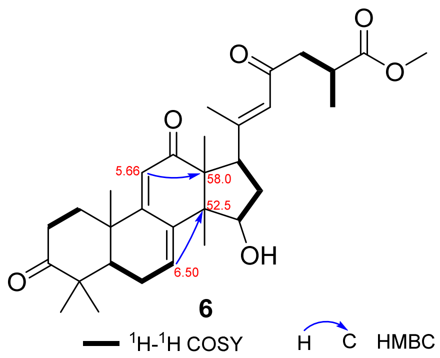

Figure S118 The key HMBC and ^{1}H-^{1}H COSY correlations of **6**.

Methyl gibbosate O (**6**): pale-yellow oil. $[\alpha]_D^{24}$ +51.4 (*c* 0.05, MeOH); UV (MeOH) λ_{max} (log ε) 245.0 (4.16); HRESIMS *m/z* 533.28693 $[M + Na]^+$ (calcd for $C_{31}H_{42}O_6Na$, 533.28791).

**No.**	**6** (600/150 MHz, $CDCl_3$)	
1	2.27, ddd (13.8, 6.1, 3.0)	35.9, CH_2
	1.87, td (13.8, 4.7)	
2	2.78, ddd (14.8, 13.8, 6.1)	34.5, CH_2
	2.43, ddd (14.8, 4.7, 3.0)	
3		215.0, C
4		47.4, C
5	1.68, dd (10.6, 5.6)	49.8, CH
6	2.29, overlapped, 2H	24.0, CH_2
7	6.50, m	131.1, CH
8		140.0, C
9		161.2, C
10		38.2, C
11	5.66, s	118.5, CH
12		203.0, C
13		58.0, C
14		52.5, C
15	4.58, t (8.4)	73.5, CH
16	2.51, dd (8.4, 7.2)	36.7, CH_2
	1.83, dd (10.9, 8.4)	
17	3.30, dd (10.9, 7.2)	45.8, CH
18	0.90, s	18.1, CH_3
19	1.29, s	21.6, CH_3
20		157.8, C
21	2.22, s	20.9, CH_3
22	6.37, s	126.0, CH
23		198.8, C
24	2.94, overlapped	47.9, CH_2
	2.55, dd (20.4, 8.6)	
25	2.95, overlapped	35.0, CH
26		176.8, C
27	1.18, d (7.0)	17.3, CH_3
28	1.11, s	25.5, CH_3
29	1.15, s	22.6, CH_3
30	1.08, s	18.4, CH_3
-O<u>CH</u>3	3.67, s	52.0, CH_3

3. Biological assays

3.1 Cytotoxicity assays

The cytotoxicity assay procedures were same as previously reported [24]. The following human tumor cell lines were used: HL-60, SMMC-7721, A-549, MCF-7, and SW-480. All cells were cultured in RPMI-1640 medium (Hyclone, Logan, UT, USA), supplemented with 10% fetal bovine serum (FBS, Hyclone) at 37 ºC in a humidified atmosphere with 5% CO_2. Cell viability was assessed by conducting colorimetric measurements of the amount of insoluble formazan formed in living cells based on the reduction of 3-(4,5-dimethylthiazol-2-yl)-2,5-diphenyltetrazolium bromide (MTT) (Sigma, St. Louis, MO, USA). Briefly, 100 μL of adherent cells was seeded into each well of a 96-well cell culture plate and allowed to adhere for 12 h before test compound addition, while suspended cells were seeded just before test compound addition, both with an initial density of 1 $\times 10^5$ cells/mL in 100 μL of medium. Each tumor cell line was exposed to the test compound at various concentrations in triplicate for 48 h, with *cis*-platin and paclitaxel (Sigma) as positive control. After the incubation, MTT (100 μg) was added to each well, and the incubation continued for 4 h at 37 oC. The cells were lysed with 100 μL of 20% SDS-50% DMF after removal of 100 μL of medium. The optical density of the lysate was measured at 595 nm in a 96-well microtiter plate reader (Bio-Rad 680). The IC_{50} value of each compound was calculated by Reed and Muench's method.

3.2 *α*-Glucosidase inhibition assays

The *α*-glucosidase inhibition assay procedures were same as previously reported [25]. A mixture containing 25 μL of different compounds (final concentrations of 0.78, 1.56, 3.13, 6.25, 12.50, 25.00, 50.00 μM), 25 μL of *α*-glucosidase (0.2 U/mL, from *Saccharomyces cerevisiae*, Sigma), and 175 μL of phosphate buffer (100 mM, pH 6.8) was pre-incubated for 10 min at room temperature. Then the reaction was started by the addition of 25 μL of 25 mM *p*-nitrophenyl-*α*-D-glucopyranoside (Sigma) and incubated for 15 min at 37 °C. The assay was conducted in a 96-well plate, and the absorbance was determined at 405 nm using a Spark™ 10 M microplate reader (Tecan). The control was prepared by adding phosphate buffer instead of the compound. The blank was prepared by adding phosphate buffer instead of *α*-glucosidase using the same method. The *α*-glucosidase inhibition rate (%) = $[(OD_{control} - OD_{control\ blank}) - (OD_{sample} - OD_{sample\ blank})] / (OD_{control} - OD_{control\ blank}) \times 100\%$. IC_{50} values were calculated through non-linear regression using GraphPad Prism5 Software. Acarbose was utilized as the positive control with an IC_{50} value of 793.79 ± 0.41 μM.

3.3 Protein tyrosine phosphate 1 β (PTP1B) inhibition assays

The PTP1B inhibition assay procedures were same as previously reported [26].

3.2 Dipeptidyl peptidase 4 (DDP4) inhibition assays

The DDP4 inhibition assay procedures were same as previously reported [27].

3.3 Angiotensin converting enzyme 2 (ACE2) inhibition assays

The ACE2 inhibition assay procedures were same as previously reported [28].
